# Total Synthesis of Jiadifenolide**

**DOI:** 10.1002/anie.201404224

**Published:** 2014-05-23

**Authors:** Ian Paterson, Mengyang Xuan, Stephen M Dalby

**Affiliations:** University Chemical Laboratory, University of CambridgeLensfield Road, Cambridge, CB2 1EW (UK)Homepage: http://www.paterson.ch.cam.ac.uk/; Department of Process Chemistry, Merck & CoPO Box 2000, Rahway, NJ 07065 (USA)

**Keywords:** cyclization, neurological agents, samarium, terpenoids, total synthesis

## Abstract

As a potent neurotrophic agent, the sesquiterpenoid jiadifenolide represents a valuable small-molecule lead for the potential therapeutic treatment of neurodegenerative diseases. A stereocontrolled total synthesis of this densely functionalized natural product is reported, central to which is an adventurous samarium-mediated cyclization reaction to establish the tricyclic core and the adjacent C5 and C6 quaternary stereocenters.

Jiadifenolide (**1**, Scheme 1) is an architecturally complex sesquiterpenoid first isolated from the pericarps of the Chinese plant *Illicium jiadifengpi* by Fukuyama and co-workers in 2009.^1^ Preliminary biological investigation revealed potent neurotrophic activity, promoting neurite outgrowth in primary cultured rat cortical neurons at concentrations as low as 10 nm. Given the important regulatory role of neurotrophins in the central nervous system, jiadifenolide represents a valuable small-molecule lead for the potential therapeutic treatment of neurodegenerative conditions such as Alzheimer’s disease.^2^ Its low natural abundance (1.5 mg kg^−1^ plant material) makes total synthesis of particular importance for the preparation of sufficient quantities for further biological evaluation and to enable access to a range of analogues for structure–activity relationship (SAR) profiling.^3^

**Scheme 1:**
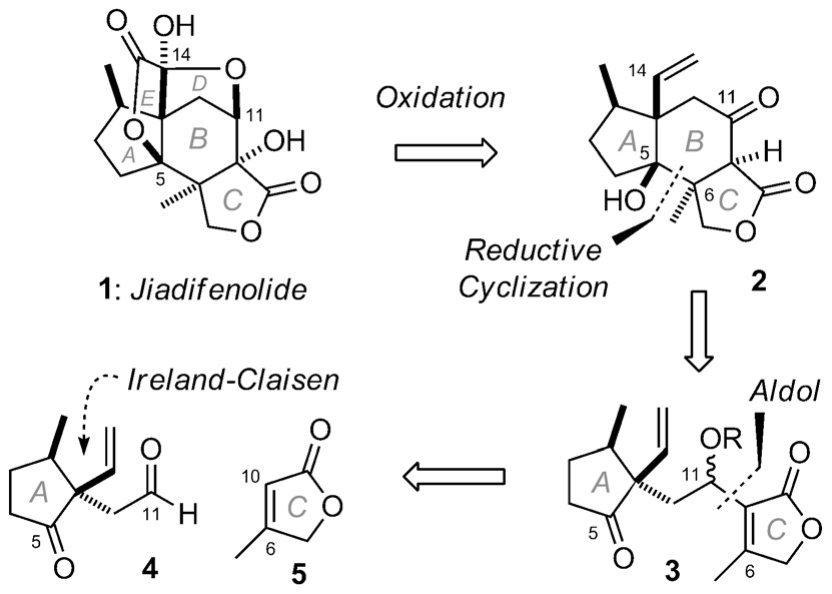
Retrosynthetic analysis of jiadifenolide (1).

Our interest in jiadifenolide derives not only from its potent bioactivity, but also from its complex *seco*-prezizaane skeleton, placing it amongst the *Illicium* family of sesquiterpenoids,^4^ which has garnered substantial synthetic attention.2a, 5 Jiadifenolide’s intricate and densely functionalized caged pentacyclic structure (**1**) comprises four rings emanating from a central, highly substituted B-ring cyclohexane. This poses particular synthetic challenges, most notably the controlled introduction of five contiguous quaternary stereocenters, which we aimed to address as part of a concise and efficient total synthesis,^6^ following that first reported by Theodorakis in 2011 and most recently by Sorensen (2014).^7^

Our conceptually unique synthetic solution sought to forge the central B-ring from a functionalized A,C-ring precursor. As outlined retrosynthetically in Scheme anie0053-7048-f1, we envisaged late-stage unveiling the D- and E-rings through oxidation of the C13-pendant vinyl group of ABC-tricycle **2**. A critical transformation would thus be the reductive cyclization of keto-butenolide **3** to establish the central B-ring and the adjacent C5 and C6 quaternary stereocenters in a single operation. Cyclization substrate **3** would be prepared through aldol coupling of butenolide **5** with aldehyde **4**. Notably, a single C5 stereocenter would template for all remaining stereochemistry under substrate-based control.

The preparation of aldehyde **4** commenced with Luche reduction of cyclopentenone **6** (Scheme anie0053-7048-f2).^8^ The ensuing allylic alcohol then underwent hydroxy-directed epoxidation with *m*-CPBA and TBS protection to deliver epoxide **7** in 65 % yield over the three steps. The C2 methyl-bearing stereocenter was then established through Lewis-acid mediated rearrangement of **7** to selectively deliver the 2,5-*syn*-configured cyclopentanone **8** (78 %, 19:1 d.r.).^9^, ^10^ Horner–Wadsworth–Emmons (HWE) homologation of **8** (73 %, >19:1 *E*:*Z*) followed by reduction and acylation of **10** then smoothly delivered allylic acetate **11** in readiness for an Ireland–Claisen rearrangement to install the key C13 quaternary stereocenter.^11^ In the event, heating the corresponding TBS ketene acetal (LDA, TBSCl) in anhydrous benzene led to the selective (10:1 d.r.) formation of a mixture of rearranged products arising from preferential reaction at the less hindered alkene π-face.^12^ This material was subjected directly to reduction with LiAlH_4_ to deliver alcohol **12** in 63 % yield from **11**. Finally, hydrolysis of the TBS ether and double oxidation under Swern conditions gave the targeted aldehyde **4** in 74 % yield (13 % overall from **6**).

**Scheme 2:**
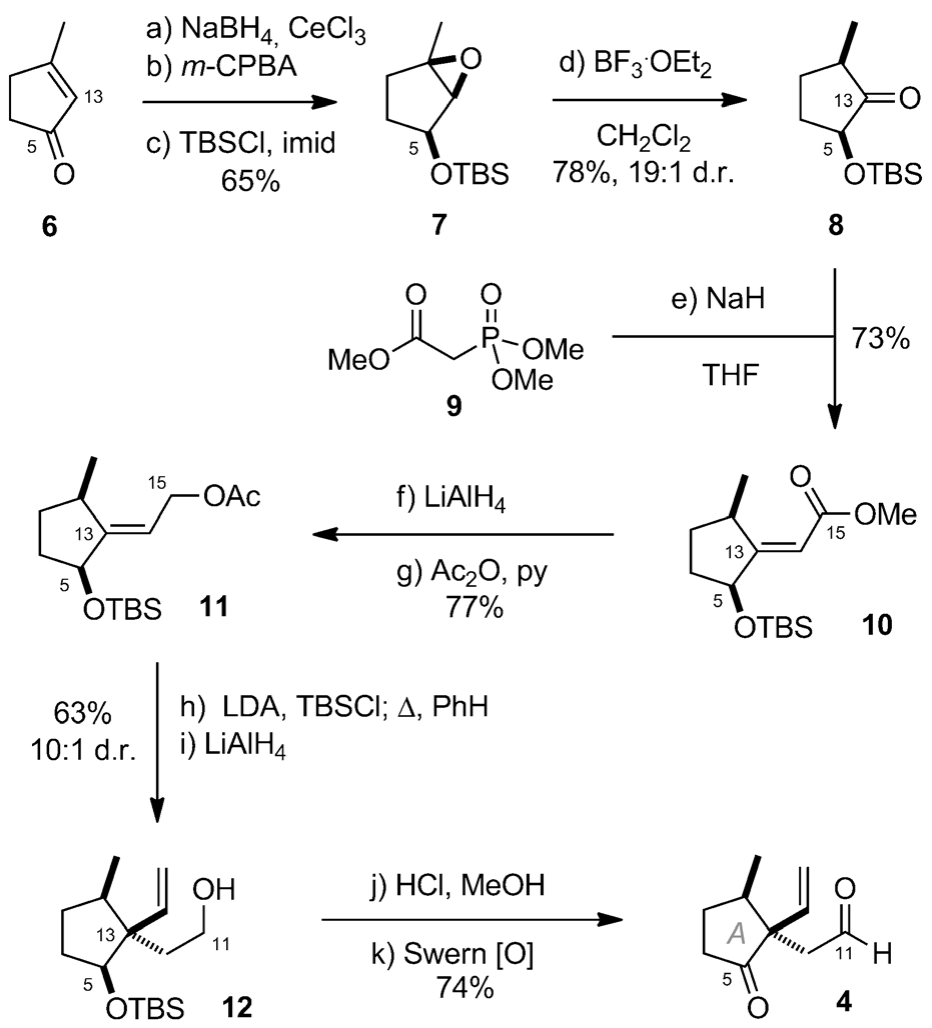
Preparation of A-ring keto-aldehyde 4. Reagents and conditions: a) NaBH_4_, CeCl_3_, MeOH, −78 °C to 0 °C; b) *m*-CPBA, CH_2_Cl_2_, 0 °C; c) TBSCl, imid, CH_2_Cl_2_, 0 °C to RT, 65 % over 3 steps; d) BF_3_⋅OEt_2_, CH_2_Cl_2_, −20 °C, 78 %, 19:1 d.r.; e) NaH, RT, 72 h, 73 %, >19:1 (*E*):(*Z*); f) LiAlH_4_, Et_2_O, 0 °C; g) Ac_2_O, py, DMAP, CH_2_Cl_2_, RT, 77 % over 2 steps; h) LDA, TBSCl, THF, −78 °C to RT; PhH, reflux, 16 h, 10:1 d.r.; i) LiAlH_4_, Et_2_O, 0 °C, 63 % over 2 steps; j) 3 n HCl, MeOH, (1:3), RT; k) DMSO, (COCl)_2_, Et_3_N, CH_2_Cl_2_, −78 °C to RT, 74 % over 2 steps. *m*-CPBA=3-chloroperoxybenzoic acid, DMAP=*N*,*N*-dimethyl-4-aminopyridine, DMSO=dimethylsulfoxide, imid=imidazole, LDA=lithium diisopropylamide, py=pyridine, TBS=*tert*-butyldimethylsilyl, THF= tetrahydrofuran.

Initial approaches at appending the C-ring butenolide through addition of 3-metallated furyl derivatives to **4** all met with failure, with optimally only trace amounts of adducts observed.^13^ By contrast (Scheme 3), it was found that butenolide **5**^14^ and aldehyde **4** could be smoothly coupled through a boron-mediated aldol reaction, which provided adducts **13** as a 2:1 mixture of diastereomeric alcohols in almost quantitative yield.^15^ At this stage, with a view to effecting the crucial reductive cyclization reaction to close the central B-ring, alcohols **13** were oxidized to provide tricarbonyl **14**. Disappointingly however, no productive cyclization of **14** could be induced under a range of conditions utilizing either SmI_2_^16^ or alternative reagents,^17^ returning only starting material or, under more forcing conditions, decomposition products. A similar situation was observed for alcohols **13**. The apparent instability of **14** with respect to potential reagents for cyclization led to the examination of TES ethers **15** and 11-*epi*-**15** as alternative substrates, which were chromatographically separable. Now, gratifyingly, it was found that addition of **15** to a freshly prepared solution of SmI_2_ (ca. 6 equiv) in THF and heating to 65 °C, led to the generation of a single cycloadduct (51 %), identified as **16** on the basis of NMR and computational analysis.^10^, ^18^ More rigorous structural proof was subsequently obtained through X-ray crystallographic analysis of alcohol **17**, formed upon acid-mediated desilylation of **16** (Figure 1 a).^19^ Subsequent oxidation with PCC then delivered the targeted ABC-ketone **2** (81 %)^19^ along with diol **18** (11 %), containing the requisite oxygenation at C10.^20^ Attempts to encourage complete oxidation to **18** were unsuccessful however.

**Figure 1:**
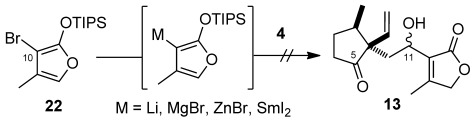
a) X-ray crystal structure of alcohol 17. b) Possible samarium-chelate transition structure leading to 16.

**Scheme 3:**
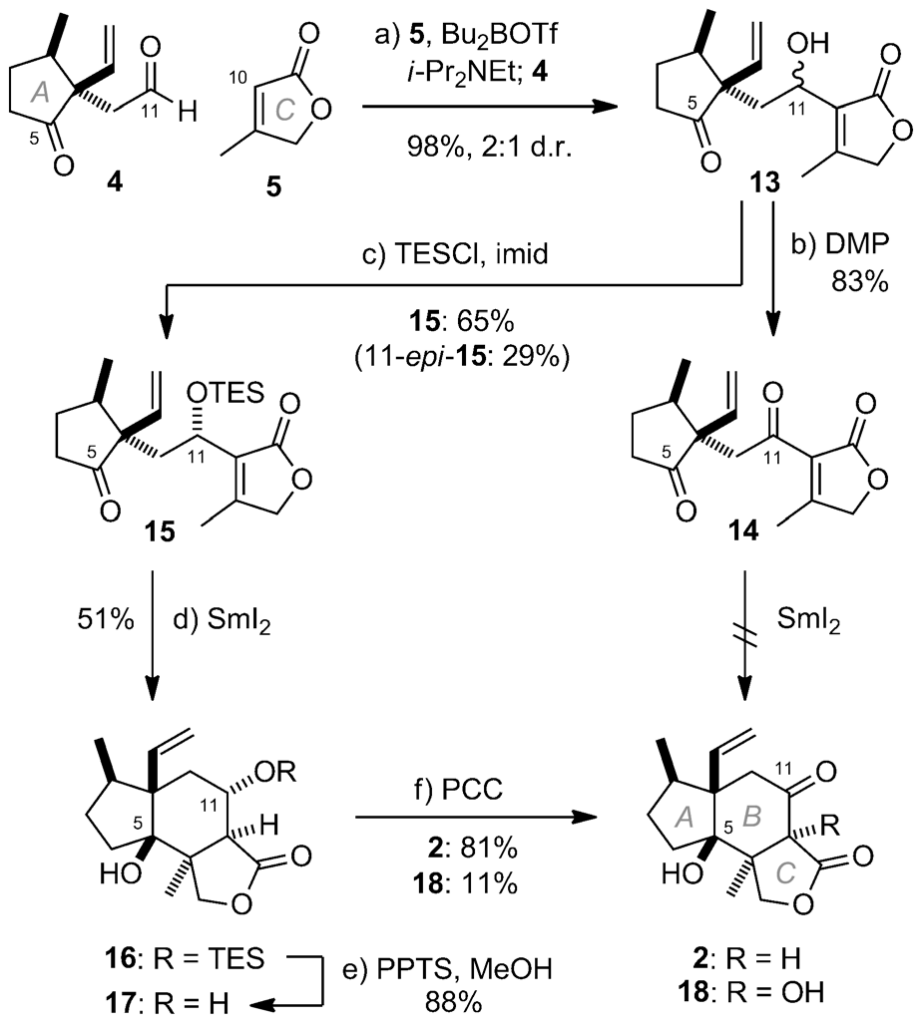
Preparation of ABC-tricycle 2. Reagents and conditions: a) Bu_2_BOTf, *i*Pr_2_NEt, THF, −78 °C to −20 °C, 98 %, 2:1 d.r.; b) DMP, NaHCO_3_, CH_2_Cl_2_, 83 %; c) TESCl, imid, DMF, RT, 15: 65 %, 11-*epi*-15: 29 %; d) SmI_2_, 65 °C, 2 h, 51 %; e) PPTS, MeOH, CH_2_Cl_2_, RT, 88 %; f) PCC, NaOAc, SiO_2_, CH_2_Cl_2_, 2: 81 %, 18: 11 %. DMF=*N*,*N*-dimethylformamide, DMP=Dess–Martin periodinane, PCC=pyridinium chlorochromate, PPTS=pyridinium *para*-toluenesulfonate, TES= triethylsilyl.

The desired stereochemical outcome of the SmI_2_-mediated reductive cyclization reaction of **15** is consistent with a chelated boat-type transition structure such as **19** (Figure 1 b), in which the C11 substituent is equatorially disposed.^21^ This may explain the failure of the epimeric C11 TES ether (natural configuration, pseudo-axially disposed) to undergo analogous cyclization, attesting, along with failed substrates **13** and **14**, to the challenge of this adventurous transformation.

With the requisite carbon skeleton of jiadifenolide secured, completion of the total synthesis was accomplished through controlled oxygenation at C10, C11, C14, and C15, with attendant cyclization to form the D,E-rings (Scheme 4). Accordingly, chemoselective dihydroxylation (OsO_4_, NMO) of the TMS-enol ether of **2** (TMSOTf, Et_3_N), cleanly provided the C10 tertiary alcohol (**18**, 99 %). This was then utilized to set the adjacent C11 alcohol stereocenter through hydroxy-directed reduction with Me_4_NBH(OAc)_3_ (87 %, >19:1 d.r.).^22^ TES protection proved necessary to facilitate the subsequent unveiling of the D,E-rings (**20**, 96 %). In the event, pyridine-accelerated dihydroxylation of the C13-pendant alkene of **20** provided a diol which then underwent smooth oxidative lactonization upon treatment with TPAP, NMO to form the E-ring (84 %).^23^ Finally, desilylation occurred with concomitant hemiacetalization to form the D-ring and provided synthetic (±)-jiadifenolide (**1**, 86 %), which exhibited spectral characteristics (^1^H/^13^C NMR, IR, HRMS) identical in all respects to that reported for the natural sample.^1^

**Scheme 4:**
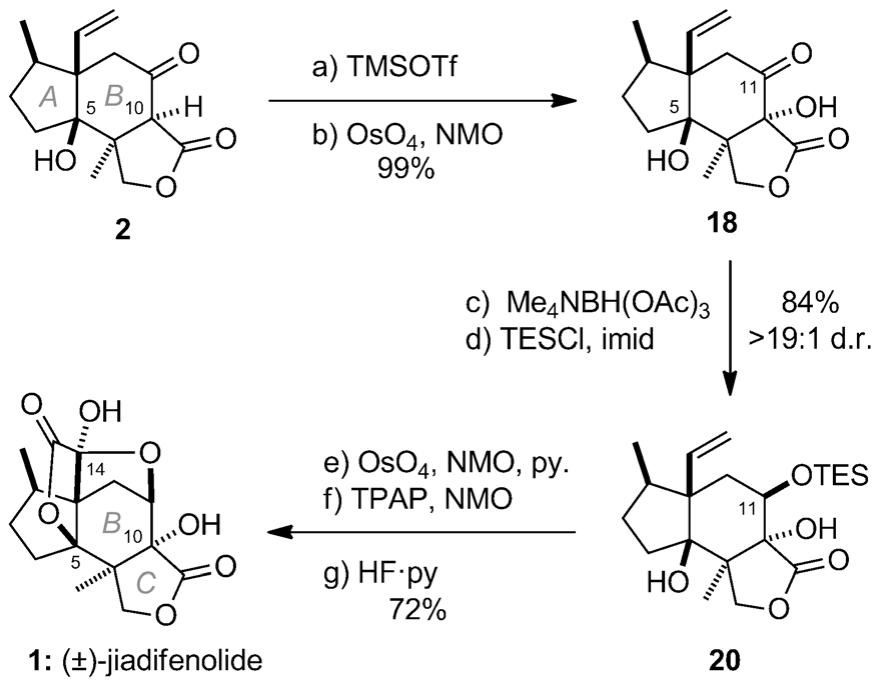
Total synthesis of jiadifenolide (1). Reagents and conditions: a) TMSOTf, Et_3_N, THF, −78 °C; b) OsO_4_, NMO, *t*BuOH/H_2_O, RT; 99 % over 2 steps; c) Me_4_NBH(OAc)_3_, AcOH, MeCN, −20 °C, 87 %, >19:1 d.r.; d) TESCl, imid, DMF, RT, 96 %; e) OsO_4_, NMO, py, *t*BuOH/H_2_O, RT; f) TPAP, NMO, 4 Å MS, CH_2_Cl_2_, RT, 84 % over 2 steps; g) HF⋅py, THF, RT, 86 %. NMO=*N*-methylmorpholine-*N*-oxide, TMS=trimethylsilyl, TPAP=tetrapropylammonium perruthenate.

In summary, we have completed the total synthesis of the neurotrophic agent jiadifenolide in 2.3 % yield over 23 steps, showcasing a pivotal SmI_2_-mediated reductive cyclization reaction to establish the tricyclic core. Notably, the synthesis demonstrates a high degree of solely substrate-based stereocontrol to establish this densely functionalized structure, wherein the full relative configuration is templated for by a single C5 alcohol stereocenter. Starting from known (*S*)-3-methyl-cyclopenten-1-ol should thus render this approach asymmetric.^24^ Work to prepare quantities of this valuable scaffold for further biological evaluation and analogue synthesis7b will be reported in due course.

Dedicated to Professor Richard J. K. Taylor on the occasion of his 65th birthday
