# Supplementary material for: Total Synthesis of Jiadifenolide
Source: Angew Chem Int Ed Engl. 2014 May 23;53(28):7286–9. doi: 10.1002/anie.201404224 (PMC4320761; doi:10.1002/anie.201404224)

Angewandte Chemie
Eine Zeitschrift der Gesellschaft Deutscher Chemiker

Supporting Information
© Wiley-VCH 2014
69451 Weinheim, Germany

Total Synthesis of Jiadifenolide**

Ian Paterson, Mengyang Xuan, and Stephen M. Dalby**

anie_201404224_sm_miscellaneous_information.pdf

Contents

1. General details S2
2. Experimental details S4
3. NMR comparison for natural and synthetic jiadifenolide (**1**) S24
4. ^{1}H and ^{13}C NMR spectra for new compounds S25

1. General details

^{1}H nuclear magnetic resonance (NMR) spectra were recorded using an internal deuterium lock for the residual protons in $CDCl_3$ (δ_H 7.26) and CD_3OD (δ_H 3.31)[1] at ambient probe temperatures on the following instruments: Bruker AVANCE BB500 or TCI500 (500 MHz). All data are presented as follows: chemical shift (in ppm relative to δ_{TMS} = 0 ppm), integration, multiplicity (s = singlet, d = doublet, t = triplet, q = quartet, qn = quintet, m = multiplet, *br* = broad, *app* = apparent), coupling constant (*J* in Hz) and assignment. Coupling constants were taken directly from the spectra. Assignments were determined either on the basis of unambiguous chemical shift or coupling patterns, COSY experiments or by analogy to fully interpreted spectra for related compounds. ^{13}C NMR spectra were recorded by broadband proton spin decoupling at ambient probe temperatures on the following instruments: Bruker AVANCE BB500 or TCI500 (125 MHz), using an internal deuterium lock for $CDCl_3$ (δ_C77.0), CD_3OD (δ_C49.0). All chemical shift values are recorded in ppm relative to δ_{TMS} = 0 ppm.

Infra-red spectra were recorded on a Perkin-Elmer Spectrum One FT-IR spectrometer fitted with a universal ATR sampling accessory. Wavelengths of maximum absorbance (ν_{max}) are quoted in cm^{-1}. High resolution mass spectra (HRMS) were recorded by the EPSRC Mass Spectrometry service (Swansea, UK) using chemical ionisation (CI), Department service (Cambridge University Chemical Laboratories, UK) using Electron Impact (EI), Fast Atom Bombardment (FAB) or Electrospray Ionisation (EI) techniques. The parent ion $[M+H]^+$, $[N+NH_4]^+$ or $[M+Na]^+$ is quoted.

Analytical thin layer chromatography (TLC) was carried out on Merck Kieselgel 60 F254 plates with visualisation by ultraviolet light (254 nm) and potassium permanganate or phosphomolybdic acid / $Ce_2(SO_4)_3$ dips. Flash chromatography was carried out on Merck Kieselgel 60 (240-400 mesh) silica under a positive pressure using distilled solvents. The procedure includes the subsequent evaporation of solvents *in vacuo*.

Reagents and solvents were purified by standard means.[2] Dichloromethane (CH_2Cl_2), acetonitrile

[1] H. E. Gottlieb, V. Kotlyar, A. Nudelman, *J. Org. Chem.* **1997**, *62*, 7512.
[2] D. A. Perrin, W. L. F. Armarego, *Purification of Laboratory Reagents*, Pergamon Press, Oxford, **1988**.

(MeCN), methanol (MeOH), toluene (PhMe), benzene (PhH) and hexane were distilled from calcium hydride and stored under an argon atmosphere. Tetrahydrofuran (THF) and diethyl ether (Et_2O) were distilled from sodium or potassium wire / benzophenone ketyl radical under an argon atmosphere. Triethylamine (Et_3N), pyridine, diisopropylamine, oxalyl chloride $(COCl)_2$ and dimethyl sulfoxide (DMSO) were distilled from and stored over calcium hydride. 4Å molecular sieves were activated by heating under high vacuum or in a microwave. All other chemicals were used as received, except where otherwise stated in the experimental text. Solvents used for all extractions in work-up were distilled. All solutions of sodium bicarbonate ($NaHCO_3$), ammonium chloride (NH_4Cl), sodium thiosulfate ($Na_2S_2O_3$) and sodium / potassium tartrate (Na^+ / K^+ tartrate) were saturated. The term “brine” describes a saturated aqueous solution of sodium chloride (NaCl).

All experiments were performed under anhydrous conditions at room temperature (RT) under an atmosphere of argon, except where stated or when water or aqueous solutions were used, using oven-dried glassware and employing standard techniques for handling air-sensitive materials.

The atom numbering system for jiadifenolide follows that proposed by Fukuyama and co-workers (Figure 1).[3]

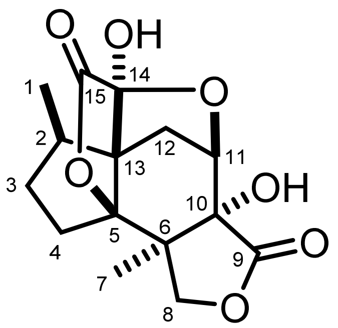

Figure 1. Atom numbering for jiadifenolide (**1**)

[3] M. Kubo, C. Okada, J.-M. Huang, K. Harada, H. Hioki, Y. Fukuyama, *Org. Lett.* **2009**, *11*, 5190.

2. Experimental details

Cyclopentenone 6 [4]

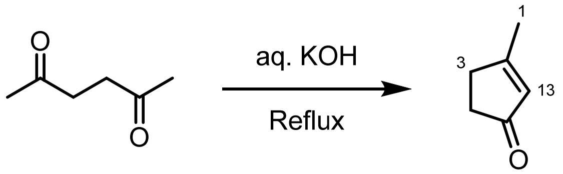

6

To a stirred solution of potassium hydroxide (5.25 g, 93.6 mmol) in H_2O (250 mL) at reflux was added hexane-2,5-dione (42.0 g, 368 mmol) over 10 min. The reaction mixture was refluxed for a further 40 min before being cooled rapidly to 0 °C, saturated with sodium chloride (45.0 g) and filtered through a pad of Celite. The solution was extracted with CH_2Cl_2 (3 × 200 mL) and the combined organic extracts were dried ($MgSO_4$) and concentrated *in vacuo*. Purification by distillation (64 °C, 9 mmHg) afforded cyclopentenone **6** (25.0 g, 71%) as a colorless oil.

^{1}H NMR (500 MHz, $CDCl_3$) δ_H 5.96 (1H, s, H13), 2.59 (2H, t, *J* = 4.5 Hz, H4), 2.43 (2H, t, *J* = 4.5 Hz, H3), 2.16 (3H, s, H1). The data was consistent with that reported in the literature.

Cyclopentenol 6a

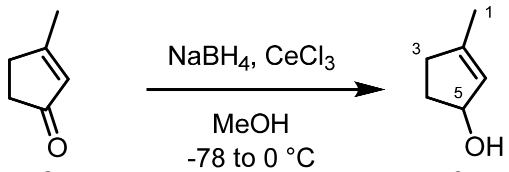

6 6a

To a stirred solution of cyclopentenone **6** (10.0 g, 104 mmol) in MeOH (250 mL) at −78 °C was added $CeCl_3{\cdot}7H_2O$ (46.6 g, 125 mmol). The reaction mixture was stirred for 15 min before $NaBH_4$ (4.73 g, 125 mmol) was added over a period of 15 min. The reaction was warmed to 0 °C and stirred for 1 h, before being warmed to RT and quenched with H_2O (20 mL). The solution was extracted with CH_2Cl_2 (3 × 100 mL) and the combined organic extracts were washed with brine (50 mL), dried ($MgSO_4$) and concentrated *in vacuo*. The crude product (9.10 g) was used without further purification.

[4] L. Bagnell, M. Bliese, T. Cablewski, C. R. Strauss, J. Tsanaktsidis, J. *Aust. J. Chem*. **1997**, *50*, 921.

^{1}H NMR (500 MHz, $CDCl_3$) δ_H 5.46 (1H, m, H13), 4.81 (1H, br s, OH), 2.44 (1H, m, H4a), 2.30 (1H, m, H4b), 2.16 (1H, m, H3a), 1.78 (3H, s, H1), 1.73 (1H, m, H3b), 1.41 (1H, d, *J* = 6.5 Hz, H5). The data was consistent with that reported in the literature.[5]

Epoxy-alcohol 6b

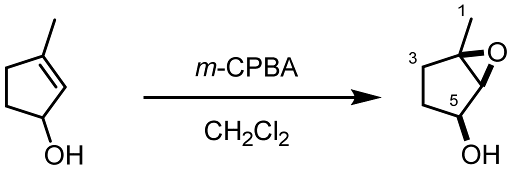

6a **6b**

To a stirred solution of crude cyclopentenol **6a** (9.10 g, 92.8 mmol) in CH_2Cl_2 (200 mL) at 0 °C was added *m*-CPBA (70 wt%, 28.3 g, 115 mmol) cautiously portionwise. The reaction mixture was stirred for 30 min at 0 °C before being quenched with $NaHCO_3$ (25 mL) and $Na_2S_2O_3$ (25 mL). The mixture was stirred vigorously for 30 min before the organic phase was separated. The aqueous phase was extracted with CH_2Cl_2 (2 × 75 mL) and the combined organic extracts were washed with $NaHCO_3$ (2 × 50 mL), dried ($MgSO_4$) and concentrated *in vacuo* afford crude epoxy-alcohol **6b** (9.75 g) as a yellow oil. The crude product was used without further purification.

^{1}H NMR (500 MHz, $CDCl_3$) δ_H 4.29 (1H, t, *J* = 7.6 Hz, H5), 3.31 (1H, s, H13), 2.00 (2H, m, H4), 1.73 (1H, br s, OH), 1.63 (1H, m, H3a), 1.46 (3H, s, H1), 1.33 (1H, m, H3b). The data was consistent with that reported in the literature.[6]

TBS-ether 7

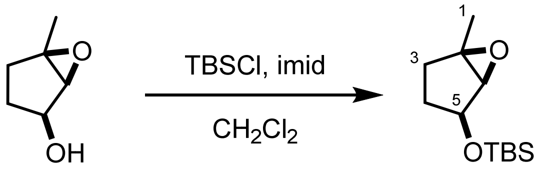

6b **7**

To a stirred solution of crude epoxy alcohol **6b** (9.75 g, 85.4 mmol) in CH_2Cl_2 (100 mL) at 0 °C was added imidazole (10.6 g, 156 mmol) and TBSCl 18.8 g, 124.9 mmol) over 5 min. The reaction was

[5] X. Zhou, P. J. De Clercq, J. Gawronski, *Tetrahedron: Asymmetry*, **1995**, *6*, 1551.
[6] D. Ye, F. Fringuelli, O. Piermatti, F. Pizzo, *J. Org. Chem.*, **1997**, *62*, 3748.

allowed to warm to RT and stirred for 1 h before being quenched with H_2O (20 mL). The mixture was extracted with CH_2Cl_2 (3 × 50 mL) and the combined organic extracts were dried ($MgSO_4$) and concentrated *in vacuo*. Purification by flash column chromatography (Et_2O / 40-60 PE, 1:19) afforded epoxy TBS ether **7** (15.4 g, 65% over 3 steps from **6**) as a colorless oil.

R_f 0.31 (Et_2O / 40-60 PE, 1:19); **v_{max}** (thin film) / cm^{-1} 2955, 2929, 1472, 1422, 1251, 1113, 1101, 886, 832, 775; **^1H NMR** (500 MHz, $CDCl_3$) δ_H 4.27 (1H, ddd, J = 8.2, 7.8, 1.2 Hz, H5), 3.16 (1H, s, H13), 1.92 (1H, dd, J = 13.8, 8.2 Hz, H3a), 1.73 (1H, dt, J = 12.2, 7.7 Hz, H4a), 1.52 (1H, m, H3b), 1.42 (1H, m, H4b), 1.40 (3H, s, H1), 0.90 (9H, s, $SiC(CH_3)_3$), 0.09 (3H, s, $SiCH_3$), 0.07 (3H, s, $SiCH_3$); **^{13}C NMR** (125 MHz, $CDCl_3$) δ_C 74.1, 65.2, 62.1, 30.0, 28.3, 25.9, 18.2, 18.0, −4.6, −4.7; **HRMS** (ES^+) calcd for $C_{12}H_{25}O_2Si$ $[M+H]^+$ 229.1618, found 229.1617.

Cyclopentanone 8

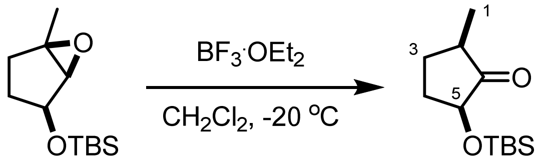

7 **8**

To a stirred solution of epoxy TBS-ether **7** (10.0 g, 43.7 mmol) in CH_2Cl_2 (200 mL) at −20 °C was added $BF_3 \cdot Et_2O$ (5.66 mL, 45.9 mmol). The reaction mixture was stirred at −20 °C for 10 min, before being quenched with $NaHCO_3$ (20 mL). The organic phase was separated, the aqueous phase extracted with CH_2Cl_2 (3 × 50 mL) and the combined organic extracts were dried ($MgSO_4$) and concentrated *in vacuo*. Purification by flash column chromatography (EtOAc / 40-60 PE, 1:40) afforded cyclopentanone **8** (15.6 g, 78%, 19:1 d.r.) as a colorless oil.

R_f 0.31 (Et_2O / 40-60 PE, 1:19); **v_{max}** (thin film) / cm^{-1} 2956, 2930, 1752, 1462, 1251, 1132, 1061, 939, 854, 776, 670; **^1H NMR** (500 MHz, $CDCl_3$) δ_H 4.07 (1H, m, H5), 2.21 (1H, m, H2), 2.05 (2H, m, H3a, H4a), 1.79 (1H, m, H4b), 1.64 (1H, m, H3b), 1.09 (3H, d, J = 7.7 Hz, H1), 0.87 (9H, s, $SiC(CH_3)_3$), 0.10 (3H, s, $SiCH_3$), 0.07 (3H, s, $SiCH_3$); **^{13}C NMR** (125 MHz, $CDCl_3$) δ_C 218.2, 75.5, 39.0, 29.9, 25.8, 25.7, 18.3, 16.1, −4.6, −5.1; **HRMS** (ES^+) calcd for $C_{12}H_{25}O_2Si$ $[M+H]^+$ 229.1618, found 229.1617.

The relative stereochemistry of **8** was determined by nOe analysis. Irradiation of H2 showed a 3% nOe enhancement to H5, indicative of a *syn*-facial relationship (Figure 2).

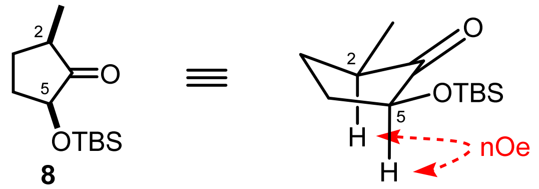

Figure 2. nOe analysis for cyclopentanone **8**.

Methyl ester 9

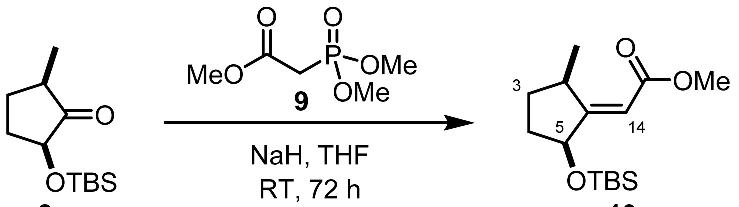

To a stirred suspension of NaH (60% in mineral oil, 4.26 g, 107 mmol) in THF (400 mL) at 0 °C was added phosphonate **9** (17.2 mL, 107 mmol). The mixture was stirred for 30 min before addition of a solution of cyclopentanone **8** (12.2 g, 53.3 mmol) in THF (10 mL). The reaction mixture was stirred at RT for 72 h before being cooled to 0 °C and quenched with NH_4Cl (20 mL). The organic phase was separated, the aqueous phase extracted with Et_2O (3 × 50 mL), and the combined organic extracts were dried ($MgSO_4$) and concentrated *in vacuo*. Purification by flash column chromatography (Et_2O / 40-60 PE, 1:24) afforded methyl ester **10** (11.6 g, 73%, >19:1 *E*:*Z*) as a colorless oil.

R_f 0.39 (Et_2O / 40-60 PE, 1:19); **ν_{max}** (thin film) / cm^{-1} 2954, 2858, 1721, 1662, 1435, 1253, 1205, 1143, 857, 836, 775; **^{1}H NMR** (500 MHz, $CDCl_3$) δ_H 5.83 (1H, m, H14), 4.40 (1H, m, H5), 3.70 (3H, s, OMe), 3.32 (1H, m, H2), 1.85 (1H, m, H4a), 1.73 (2H, m, H4b, H3a), 1.57 (1H, m, H3b), 1.22 (3H, d, *J* = 7.2 Hz, H1), 0.92 (9H, s, $SiC(CH_3)_3$), 0.11 (3H, s, $SiCH_3$), 0.10 (3H, s, $SiCH_3$); **^{13}C NMR** (125 MHz, $CDCl_3$) δ_C 172.5, 166.8, 112.8, 77.4, 50.8, 35.4, 32.3, 29.7, 25.7, 20.6, 18.0, -4.7, -4.8; **HRMS** (ES^+) calcd for $C_{15}H_{28}O_3Si$ $[M+H]^+$ 258.1878, found 258.1880.

Allylic alcohol 10a

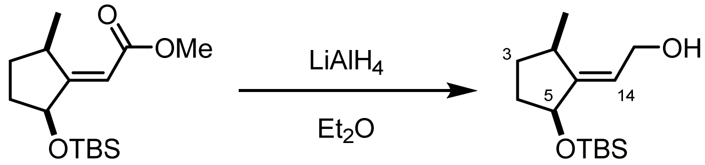

10 **10a**

To a stirred solution of methyl ester **10** (6.00 g, 20.1 mmol) in Et_2O (200 mL) at 0 °C was added $LiAlH_4$ (1.53 g, 40.3 mmol). The reaction mixture was stirred for 45 min at 0 °C before being quenched by cautious sequential addition of H_2O (1.6 mL), 15% aq. NaOH (1.6 mL) and H_2O (4.8 mL). The mixture was stirred vigorously for 1 h at RT until a fine slurry had developed. The mixture was dried ($MgSO_4$), and concentrated *in vacuo*. Purification by flash column chromatography (Et_2O / 40-60 PE, 1:4 to 1:2) afforded allylic alcohol **10a** (4.02 g, 78%) as a colorless oil.

$\mathbf{R_f}$ 0.13 (Et_2O / 40-60 PE, 1:9); $\mathbf{v_{max}}$ (thin film) / cm^{-1} 3306, 2956, 2929, 1462, 1251, 1104, 1046, 921, 834, 773; **^{1}H NMR** (500 MHz, $CDCl_3$) δ_H 5.59 (1H, t, *J* = 6.8 Hz, H14), 4.36 (1H, dd, *J* = 6.0, 5.7 Hz, H5), 4.22 (2H, d, *J* = 6.5 Hz, H15), 2.70 (1H, s, H2), 1.8-1.6 (3H, m, H3a, H4), 1.52 (2H, m, H4b, O<u>H</u>), 1.12 (3H, d, *J* = 7.2 Hz, H1), 0.91 (9H, s, $SiC(CH_3)_3$), 0.09 (6H, s, 2 × $SiCH_3$); **^{13}C NMR** (125 MHz, $CDCl_3$) δ_C 152.9, 122.0, 76.9, 60.1, 33.7, 33.3, 30.5, 25.8, 22.6, 18.0, −4.5, −4.7; **HRMS** (ES^+) calcd for $C_{14}H_{32}O_2NSi$ $[M+NH_4]^+$ 274.2197, found 274.2198.

Allylic acetate 11

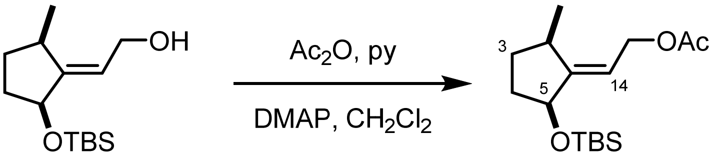

10a **11**

To a stirred solution of allylic alcohol **10a** (7.30 g, 28.5 mmol), pyridine (4.59 mL, 57.0 mmol) and DMAP (174 mg, 1.43 mmol) in CH_2Cl_2 (250 mL) at 0 °C was added Ac_2O (4.03 mL, 42.8 mmol). The reaction was allowed to warm to RT and stirred for 40 min before being quenched with $NaHCO_3$ (20 mL). The organic phase was separated, the aqueous phase extracted with CH_2Cl_2 (2 × 50 mL), and the combined organic extracts were dried ($MgSO_4$) and concentrated *in vacuo*. Purification by flash column chromatography (Et_2O / 40-60 PE, 1:9) afforded allylic acetate **11** (8.40 g, 99%) as a colorless oil.

R_f 0.39 (Et_2O / 40-60 PE, 1:9); **v_{max}** (thin film) / cm^{-1} 2957, 2930, 1743, 1363, 1228, 1111, 1048, 836, 775; **^{1}H NMR** (500 MHz, $CDCl_3$) δ_H 5.52 (1H, m, H14), 4.67 (1H, dd, *J* = 12.6, 8.2 Hz, H15a), 4.59 (1H, dd, *J* = 12.6, 6.0 Hz, H15b), 4.35 (1H, dd, *J* = 6.0, 5.8 Hz, H5), 2.74 (1H, m, H2), 2.05 (3H, s, C(O)Me), 1.80 (1H, m, H3a), 1.70 (2H, m, H4), 1.53 (1H, m, H3b), 1.12 (3H, d, *J* = 7.2 Hz, H1), 0.90 (9H, s, $SiC(CH_3)_3$), 0.07 (6H, s, 2 × $SiCH_3$); **^{13}C NMR** (125 MHz, $CDCl_3$) δ_C 170.8, 155.2, 117.0, 76.8, 61.8, 33.6, 33.3, 30.5, 25.7, 22.4, 20.8, 18.0, −4.6, −4.7; **HRMS** (ES+) calcd for $C_{16}H_{34}O_3NSi$ $[M+NH_4]^+$316.2302, found 316.2303.

Carboxylic Acid 11c

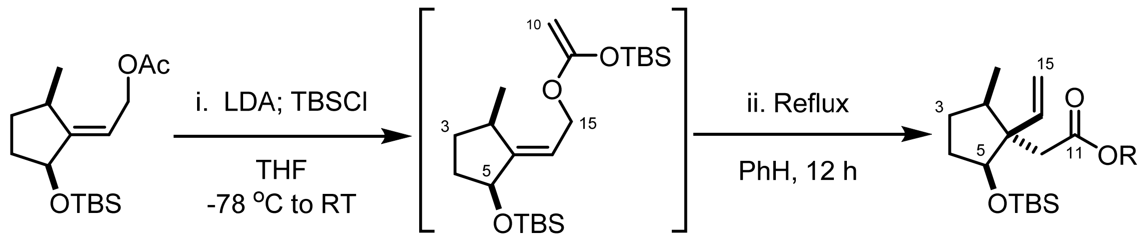

11 **11a** **11b**: R = TBS
11c: R = H

To a stirred solution of diisopropylamine (2.35 mL, 16.8 mmol) in THF (100 mL) at 0 °C was added *n*-BuLi (1.6 M in hexane, 10.0 mL, 16.0 mmol). The mixture was stirred for 30 min at 0 °C before being cooled to −78 °C and a solution of allylic acetate **11** (4.00 g, 13.4 mmol) in THF (60 mL) added. The reaction mixture was stirred for 30 min, then a solution of TBSCl (2.63 g, 17.5 mmol) in THF (5 mL) was added and stirring was continued for a further 30 min at −78 °C. The reaction mixture was then allowed to warm to RT over 1 h and stirred for a further 30 min. The reaction was diluted with hexane (50 mL), washed with H_2O (2 × 40 mL), dried ($MgSO_4$) and concentrated *in vacuo* to afford the crude silyl ketene acetal **11a** as a light yellow oil which was used without further purification.

^{1}H NMR (500 MHz, C_6D_6) 5.87 (1H, t, *J* = 6.7 Hz, H14), 4.39 (3H, m, H5, 2 × H15), 3.73 (1H, d, *J* = 2.4 Hz, H10a), 3.40 (1H, d, *J* = 2.4 Hz, H10b), 2.55 (1H, m, H2), 1.83-1.36 (4H, m, 2 × H3, 2 × H4), 1.16 (3H, d, *J* = 7.2 Hz, H1), 1.13 (9H, s, $SiC(CH_3)_3$), 1.11 (9H, s, $SiC(CH_3)_3$), 0.22 (6H, s, 2 × $SiCH_3$), 0.19 (6H, s, 2 × $SiCH_3$).

The crude silyl ketene acetal **11a** was taken up in benzene (100 mL) and heated to reflux for 12 h. The mixture was cooled to RT and concentrated *in vacuo* to afford a mixture of carboxylic acid **11b** and silyl ester **11c** which was used without further purification.

For the purposes of characterization, a small sample of an analogous reaction mixture was hydrolyzed (K_2CO_3, THF / MeOH / H_2O) and chromatographically purified (SiO_2, EtOAc / 40−60 PE, 1:20) to provide carboxylic acid **11c** (104 mg, 353 μmol) as a colorless oil.

R_f 0.19 (EtOAc / 40-60 PE, 1:10); **v_{max}** (thin film) / cm^{-1} 3300-2500 br, 2957, 1708, 1463, 1256, 1137, 1106, 871, 836, 775, 670; **^{1}H NMR** (500 MHz, $CDCl_3$) δ_H 10.90 (1H, br s, COOH), 5.83 (1H, dd, *J* = 17.8, 11.3 Hz, H14), 5.30 (1H, d, *J* = 11.3 Hz, H15a), 5.10 (1H, d, *J* = 17.8 Hz, H15b), 4.25 (1H, t, *J* = 8.6 Hz, H5), 2.82 (1H, d, *J* = 15.4 Hz, H12a), 2.47 (1H, d, *J* = 15.4 Hz, H12b), 2.05 (1H, m, H4a), 1.82 (2H, m, H2, H4b), 1.69 (1H, m, H3a), 1.40 (1H, m, H3b), 0.91 (9H, s, $SiC(CH_3)_3$), 0.86 (3H, d, *J* = 6.4 Hz, H1), 0.12 (3H, s, $SiCH_3$), 0.10 (3H, s, $SiCH_3$); **^{13}C NMR** (125 MHz, $CDCl_3$) δ_C 175.7, 135.3, 116.9, 80.3, 52.3, 40.1, 38.7, 30.3, 28.5, 25.8, 18.1, 14.7, −3.9, −4.8; **HRMS** (ES^+) calcd for $C_{16}H_{31}O_3Si$ $[M+H]^+$ 299.2042, found 299.2052.

The relative stereochemistry of carboxylic acid **11c** was determined by nOe analysis. Irradiation of H5 showed a 2% nOe enhancement to H12b, whilst irradiation of H14 showed a 1.3% nOe enhancement to H1. Together this indicated the stereostructure for **11c** to be that shown in Figure 3.

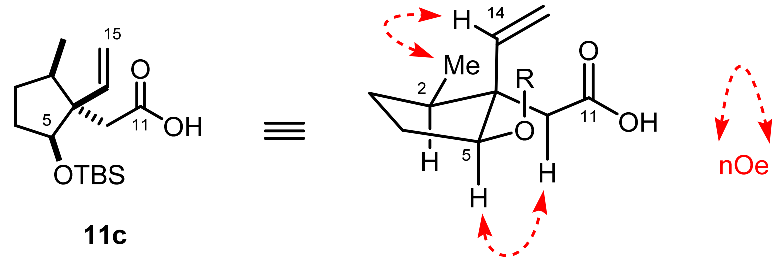

11c

Figure 3. nOe analysis for carboxylic acid **11c**.

Alcohol 11

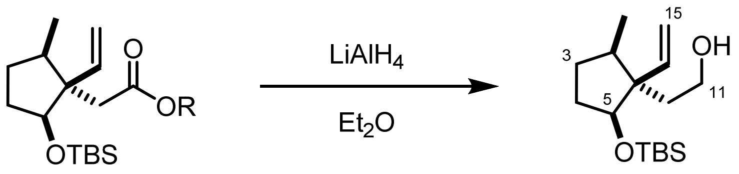

11b: R = TBS **12**
11c: R = H

To a stirred solution of the crude mixture of carboxylic acid **11b** and silyl ester **11c** in Et_2O (150 mL) at 0 °C was added $LiAlH_4$ (1.38 g, 36.3 mmol) cautiously portionwise. The reaction mixture was stirred at 0 °C for 1 h before being quenched by sequential addition of H_2O (1.38 mL), 15% aq. NaOH (1.38 mL) and H_2O (4.14 mL). The mixture was stirred vigorously at RT for 1 h until a suspension was formed. The mixture was then dried ($MgSO_4$) and concentrated *in vacuo*. Purification by flash column chromatography (Et_2O / 40-60 PE, 1:3) afforded alcohol **12** (3.58 g, 63% over 3 steps from **11**) as a colorless oil.

R_f 0.48 (Et_2O / 40-60 PE, 1:1); **v_{max}** (thin film) / cm^{-1} 3310, 2956, 2859, 1472, 1256, 1135, 1029, 911, 837, 774; **^{1}H NMR** (500 MHz, $CDCl_3$) δ_H 5.75 (1H, dd, *J* = 18.0, 11.5 Hz, H14), 5.29 (1H, dd, *J* = 11.5, 1.7 Hz, H15a), 5.15 (1H, dd, *J* = 18.0, 1.7 Hz, H15b), 4.06 (1H, t, *J* = 8.5 Hz, H5), 3.80 (1H, m, H11a), 3.74 (1H, m, H11b), 2.33 (1H, t, *J* = 6.7 Hz, OH), 2.03 (2H, m, H4a, H12a), 1.72-1.61 (3H, m, H4b, H3a, H2), 1.52 (1H, m, H12b), 1.34 (1H, m, H3b), 0.91 (9H, s, $SiC(CH_3)_3$), 0.85 (3H, d, *J* = 6.7 Hz, H1), 0.10 (3H, s, $SiCH_3$), 0.09 (3H, s, $SiCH_3$); **^{13}C NMR** (125 MHz, $CDCl_3$) δ_C 136.4, 116.4, 80.8, 59.7, 52.7, 40.9, 38.8, 30.8, 28.9, 25.9, 18.0, 14.8, −3.4, −4.8; **HRMS** (ES^+) calcd for $C_{16}H_{33}O_2Si$ $[M+H]^+$ 285.2244, found 285.2246.

Diol 12a

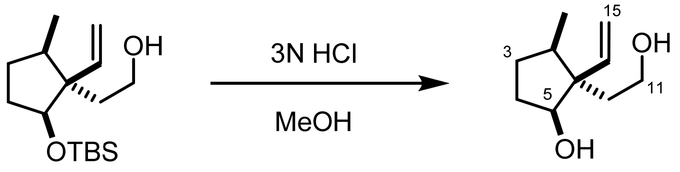

12 **12a**

To a stirred solution of alcohol **12** (3.58 g, 12.6 mmol) in MeOH (80 mL) at 0 °C was added 3N HCl (10 mL). The reaction mixture was stirred at RT for 1 h before being partitioned between EtOAc (3 × 50 mL) and H_2O (50 mL). The combined organic extracts were washed with brine (50 mL), dried

($MgSO_4$) and concentrated *in vacuo*. Purification by flash chromatography (EtOAc / 40-60 PE, 1:1) afforded diol **12a** (2.12 g, 99%) as a colorless oil.

R$_f$ 0.31 (EtOAc / 40-60 PE, 1:1); **v$_{max}$** (thin film) / cm^{-1} 3315, 2954, 2873, 1636, 1455, 1337, 1119, 1054, 1013, 914; **^{1}H NMR** (500 MHz, $CDCl_3$) δ_H 5.74 (1H, dd, *J* = 18.1, 11.5 Hz, H14), 5.41 (1H, dd, *J* = 11.5, 1.5 Hz, H15a), 5.09 (1H, dd, *J* = 18.1, 1.5 Hz, H15b), 4.03 (1H, t, *J* = 9.1 Hz, H5), 3.92 (1H, td, *J* = 11.5, 2.3 Hz, H11a), 3.80 (1H, m, H11b), 3.65 (1H, br s, OH), 3.00 (1H, br s, OH), 2.13 (1H, m, H4a), 2.07 (1H, ddd, *J* = 14.9, 4.3, 2.8 Hz, H12a), 1.75 (1H, m, H3a), 1.66 (2H, m, H4b, H2), 1.44 (1H, m, H12b), 1.33 (1H, m, H3b), 0.83 (3H, d, *J* = 6.7 Hz, H1); **^{13}C NMR** (125 MHz, $CDCl_3$) δ_C 135.2, 118.0, 79.9, 59.6, 52.8, 42.3, 38.5, 29.4, 28.6, 14.6; **HRMS** (ES$^+$) calcd for $C_{10}H_{19}O_2$ [M+H]$^+$ 171.1380, found 171.1377.

Keto-aldehyde 4

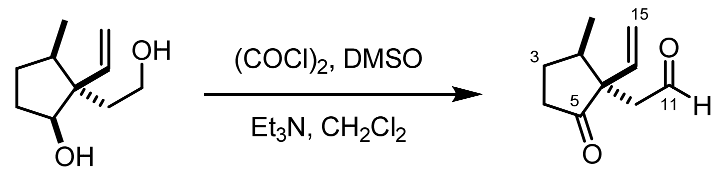

12a **4**

To a stirred solution of $(COCl)_2$ (2.45 mL, 3.62 mmol) in CH_2Cl_2 (30 mL) at −78 °C was added DMSO (4.05 mL, 4.46 mmol). The mixture was stirred for 30 min before addition of a solution of diol **12a** (1.62 g, 9.52 mmol) in CH_2Cl_2 (20 mL). The mixture was stirred at −78 °C for 30 min, then Et_3N (13.4 mL, 9.63 mmol) was added and the mixture was stirred for a further 1 h before being allowed to warm to RT over 30 min. The reaction was quenched with NH_4Cl (10 mL) and the mixture was partitioned between CH_2Cl_2 (3 × 50 mL) and H_2O (50 mL). The combined organic extracts were dried ($MgSO_4$) and concentrated *in vacuo*. Purification by flash chromatography (EtOAc / 40-60 PE, 3:7) afforded keto-aldehyde **4** (1.59 g, 75%) as a yellow oil.

R$_f$ 0.62 (EtOAc / 40-60 PE, 1:1); **v$_{max}$** (thin film) / cm^{-1} 2964, 2878, 1737, 1720, 1632, 1461, 1403, 1179, 1071, 997, 925; **^{1}H NMR** (500 MHz, $CDCl_3$) δ_H 9.72 (1H, dd, *J* = 2.1, 1.2 Hz, H11), 5.74 (1H, dd, *J* = 17.6, 10.9 Hz, H14), 5.28 (1H, dd, *J* = 10.9, 1.5 Hz, H15a), 5.02 (1H, dd, *J* = 17.6, 1.5 Hz, H15b), 2.81 (1H, dd, *J* = 17.6, 1.2 Hz, H12a), 2.63 (1H, dd, *J* = 17.6, 2.1 Hz, H12b), 2.41 (2H, m, 2 × H4), 2.26 (1H, m, H2), 2.08 (1H, m, H3a), 1.60 (1H, m, H3b), 1.04 (3H, d, *J* = 6.9 Hz, H1); **^{13}C NMR**

(125 MHz, $CDCl_3$) δ_C 218.9, 200.7, 133.4, 118.0, 56.7, 47.0, 39.6, 37.0, 27.6, 14.3; **HRMS** (ES^+) calcd for $C_{10}H_{13}O_2$ $[M+H]^+$ 165.0910, found 165.0907.

Butenolide 5 [7]

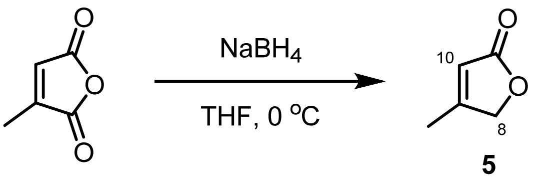

5

To a stirred solution of citraconic anhydride (5.00 g, 44.6 mmol) in THF (100 mL) at 0 °C was added $NaBH_4$ (4.26 g, 112 mmol) portionwise. The reaction mixture was stirred for 2 h before being quenched with H_2O (30 mL). The mixture was acidified (pH 1) with HCl (3 N) and the organic phase separated. The aqueous phase was extracted with EtOAc (3 × 50 mL) and the combined organic extracts were dried ($MgSO_4$) and concentrated *in vacuo*. Purification by flash column chromatography (EtOAc / 40-60 PE, 7:3) afforded butenolide **5** (3.08 g, 70%) as a colorless oil; **^{1}H NMR** (500 MHz, $CDCl_3$) δ_H 5.84 (1H, s, H10), 4.71 (2H, s, 2 × H8), 2.12 (3H, s, H7). The data was consistent with that reported in the literature.

Aldol Adducts 13

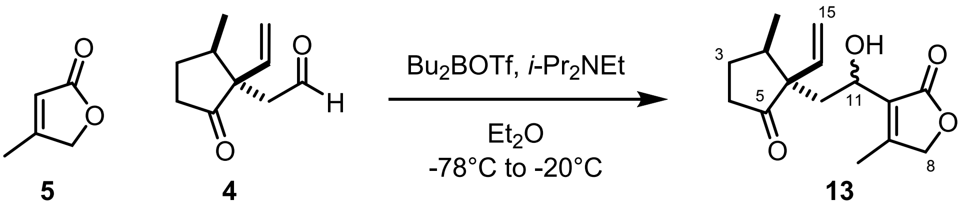

5 4 13

To a stirred solution of butenolide **5** (903 mg, 9.21 mmol) and *i*-Pr_2NEt (2.14 mL, 12.3 mmol) in THF (20 mL) at −78 °C was added dropwise a solution of Bu_2BOTf (2.52 g, 9.21 mmol) in CH_2Cl_2 (2.5 mL). The reaction mixture was stirred at −78 °C for 1 h before addition of a solution of aldehyde **4** (1.02 g, 6.14 mmol) in THF (16 mL). The reaction mixture was stirred at -20 °C for 1 h before being quenched with H_2O (30 mL). The mixture was extracted with EtOAc (3 × 50 mL) and the combined organic extracts were washed with brine (30 mL), dried ($MgSO_4$) and concentrated *in vacuo*. Purification by flash chromatography (EtOAc / 40-60 PE, 1:1) afforded aldol adducts **13** as an inseparable 2:1 mixture of alcohol diastereomers (2.39 g, 98%).

[7] S. Gogoi, N. P. Argade, *Tetrahedron* **2006**, *62*, 2715.

R$_f$ 0.26 (EtOAc / 40-60 PE, 1:1); **v$_{max}$** (thin film) / cm^{-1} 3399, 2925, 1728, 1673, 1439, 1340, 1101, 1015, 992, 914, 729; **^{1}H NMR** (500 MHz, $CDCl_3$) δ_H 5.88 (0.7H, dd, *J* = 17.5, 11.0 Hz, H14), 5.85 (0.7H, s, OH), 5.75 (0.3H, dd, *J* = 17.9, 11.0 Hz, H14), 5.33 (0.3H, d, *J* = 11.0 Hz, H15a), 5.23 (0.7H, d, *J* = 11.0 Hz, H15a), 5.15 (0.3H, m, H11), 5.09 (0.7H, dd, *J* = 9.6, 7.5 Hz, H11), 4.98 (0.7H, d, *J* = 17.5 Hz, H15b), 4.90 (0.3H, d, *J* = 17.9 Hz, H15b), 4.71 (1 H, m, H8a), 4.68 (1H, s, H8b), 4.59 (0.3H, s, OH), 2.52-1.35 (5H, m, H2, 2 × H3, 2 × H4), 2.18 (1H, s, H7), 2.13 (2H, s, 2 × H7), 1.07 (1H, d, *J* = 6.7 Hz, H1), 0.88 (2H, d, *J* = 6.8 Hz, 2 × H1). **^{13}C NMR** (125 MHz, $CDCl_3$) δ_C 224.3, 220.5, 174.5, 173.7, 160.9, 158.6, 135.4, 133.6, 128.2, 125.8, 117.1, 115.5, 72.6, 69.1, 64.2, 62.6, 59.7, 41.8, 39.7, 39.4, 37.2, 34.1, 32.9, 29.7, 29.5, 27.3, 15.5, 13.7, 12.4, 12.1; **HRMS** (ES$^+$) calcd for $C_{15}H_{21}O_4$ [M+H]$^+$ 265.1440, found 265.1447.

Tricarbonyl 15

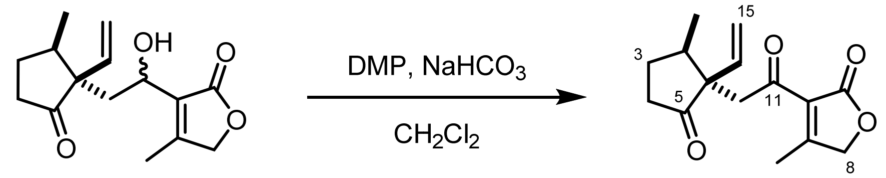

13 **14**

To a stirred solution of a mixture of aldol adducts **13** (175 mg, 0.662 mmol) in CH_2Cl_2 (10 mL) was added $NaHCO_3$ (509 mg, 5.92 mmol) and DMP (835 mg, 1.97 mmol). The mixture was stirred for 16 h before being quenched with $NaHCO_3$ / $Na_2S_2O_3$ (1:1, 5 mL). The mixture was stirred vigorously for 30 min before being diluted with H_2O (5 mL) and extracted with CH_2Cl_2 (2 × 20 mL). The combined organic extracts were dried ($MgSO_4$) and concentrated *in vacuo*. Purification by flash chromatography (EtOAc / 40-60 PE, 7:3) afforded tricarbonyl **14** (144 mg, 83%) as a colorless oil.

R$_f$ 0.51 (EtOAc / 40-60 PE, 7:3); **v$_{max}$** (thin film) / cm^{-1} 2962, 1741, 1688, 1627, 1381, 1308, 1172, 1052, 1020, 979, 923, 690; **^{1}H NMR** (500 MHz, $CDCl_3$) δ_H 5.71 (1H, dd, *J* = 17.6, 10.9 Hz, H14), 5.21 (1H, d, *J* = 10.9 Hz, H15a), 5.02 (1H, d, *J* = 17.7 Hz, H15b), 4.76 (2H, s, 2 × H8), 3.52 (1H, d, *J* = 19.4 Hz, H12a), 3.31 (1H, d, *J* = 19.4, H12b), 2.59 (1H, m, H4a), 2.50 (1H, m, H2), 2.39 (3H, s, H7), 2.38 (1H, m, H4b), 2.09 (1H, m, H3a), 1.56 (1H, m, H3b), 1.00 (3H, d, *J* = 6.9 Hz, H1), **^{13}C NMR** (125 MHz, $CDCl_3$) δ_C 219.6, 194.0, 174.7, 170.9, 133.8, 125.0, 116.9, 72.5, 56.5, 45.0, 37.7, 37.3, 27.6, 14.7, 14.5; **HRMS** (ES$^+$) calcd for $C_{15}H_{18}O_4Na$ [M+Na]$^+$ 285.1102, found 285.1100.

TES ethers 15 & **11-*epi*-15**

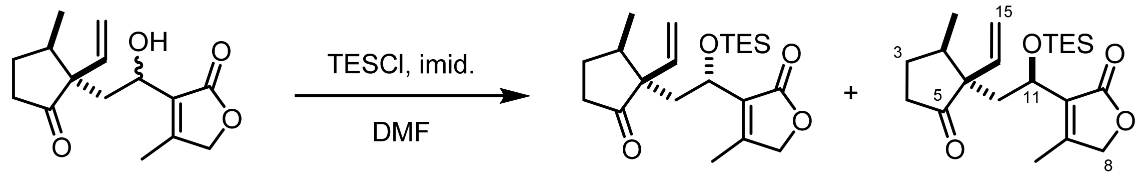

13 **15** 11-*epi*-**15**

To a stirred solution of a mixture of aldol adducts **13** (1.60 g, 6.02 mmol, 2:1 d.r.) in DMF (20 mL) at 0 °C was added imidazole (820 mg, 12.0 mmol) and TESCl (998 mg, 6.62 mmol). The reaction mixture stirred for 16 h at RT before being quenched with H_2O (20 mL). The mixture was extracted with Et_2O (2 × 50 mL) and the combined organic extracts were washed with H_2O (50 mL), dried ($MgSO_4$) and concentrated *in vacuo*. Purification by flash chromatography (EtOAc / 40-60 PE, 1:5 to 1:1) afforded TES ethers **15** (1.48 g, 65%) and 11-*epi*-**15** (680 mg, 29%) as colorless oils.

15: $\mathbf{R_f}$ 0.63 (EtOAc / 40-60 PE, 1:1); $\mathbf{v_{max}}$ (thin film) / cm^{-1} 3431, 2955, 2877, 1751, 1673, 1459, 1413, 1239, 1035, 1004, 746; **^{1}H NMR** (500 MHz, $CDCl_3$) δ_H 5.71 (1H, dd, *J* = 17.7, 10.9 Hz, H14), 5.16 (1H, d, *J* = 10.9 Hz, H15a), 4.90 (1H, d, *J* = 17.7 Hz, H15b), 4.76 (1H, dd, *J* = 8.6, 4.8 Hz, H11), 4.59 (2H, s, 2 × H8), 2.32 (2H, m, H12a, H4a), 2.23 (5H, m, H7, H4b, H2), 2.00 (1H, m, H3a), 1.80 (1H, dd, *J* = 14.6, 4.8 Hz, H12b), 1.52 (1H, m, H3b), 1.06 (3H, d, *J* = 7.0 Hz, H1), 0.91 (9H, t, *J* = 7.9 Hz, $SiCH_2C\underline{H}_3$), 0.52 (6H, q, *J* = 7.9 Hz, $SiC\underline{H}_2CH_3$); **^{13}C NMR** (125 MHz, $CDCl_3$) δ_C 218.8, 173.3, 160.0, 135.3, 129.9, 116.8, 72.4, 63.3, 57.2, 39.8, 37.8, 37.1, 27.0, 14.3, 13.0, 6.7, 4.7; **HRMS** (ES+) calcd for $C_{21}H_{35}O_4Si$ $[M+H]^+$ 379.2299, found 379.2301.

11-*epi*-**15**: $\mathbf{R_f}$ 0.67 (EtOAc / 40-60 PE, 1:1); $\mathbf{v_{max}}$ (thin film) / cm^{-1} 2956, 2877, 1741, 1675, 1458, 1405, 1241, 1066, 1038, 993, 956, 927, 836, 778, 745; **^{1}H NMR** (500 MHz, $CDCl_3$) δ_H 5.66 (1H, dd, *J* = 17.7, 10.9 Hz, H14), 5.10 (1H, d, *J* = 10.9 Hz, H15a), 4.83 (1H, d, *J* = 17.7 Hz, H15b), 4.75 (1H, dd, *J* = 8.9, 5.0 Hz, H11), 4.59 (1H, d, *J* = 17.2 Hz, H8a), 4.52 (1H, d, *J* = 17.2 Hz, H8b), 2.47 (1H, m, H2), 2.39 (1H, dd, *J* = 14.2, 8.9 Hz, H12a), 2.24 (1H, dd, *J* = 19.3, 8.5 Hz, H4a), 2.18 (3H, s, H7), 2.10 (1H, dd, *J* = 19.3, 8.5 Hz, H4b), 2.01 (1H, dd, *J* = 14.2, 5.0 Hz, H12b), 1.99 (1H, m, H3a), 1.48 (1H, m, H3b), 1.07 (3H, d, *J* = 6.9 Hz, H1), 0.91 (9H, t, *J* = 8.0 Hz, $SiCH_2C\underline{H}_3$), 0.55 (6H, m, $Si\underline{C}H_2CH_3$); **^{13}C NMR** (125 MHz, $CDCl_3$) δ_C 219.9, 173.4, 161.0, 135.6, 128.8, 116.2, 72.4, 63.0, 57.8, 39.7, 38.0, 37.4, 26.8, 14.0, 12.9, 6.7, 4.6; **HRMS** (ES^+) calcd for $C_{21}H_{35}O_4Si$ $[M+H]^+$ 379.2299, found 379.2299.

TES-Tricycle 16

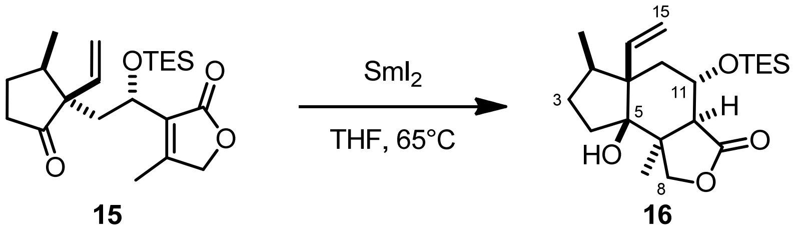

A solution of I_2 (470 mg, 1.85 mmol) in THF (4 mL) was added to a flask containing Sm metal (40 mesh, 476 mg, 3.17 mmol). The mixture was heated to reflux for 2 h (deep blue solution formed) before being cooled to RT. A solution of TES ether **15** (200 mg, 0.538 mmol) in THF (10 mL) was then added and the mixture was stirred at RT for 30 min. The reaction mixture was warmed to 65 °C over 1 h, stirred for a further 1 h before being cooled to 0 °C and quenched with NH_4Cl (10 mL). The mixture was extracted with CH_2Cl_2 (3 × 20 mL) and the combined organic extracts were dried ($MgSO_4$) and concentrated *in vacuo*. Purification by flash column chromatography (EtOAc / 40-60 PE, 3:7) afforded TES-tricycle **16** (103 mg, 51%) as a colorless oil.

$\mathbf{R_f}$ 0.43 (EtOAc / 40-60 PE, 3:7); $\mathbf{v_{max}}$ (thin film) / cm^{-1} 3500, 2957, 2877, 1761, 1459, 1375, 1239, 1173, 1109, 1007, 911, 829, 767, 746; **^{1}H NMR** (500 MHz, $CDCl_3$) δ_H 6.28 (1H, dd, *J* = 18.2, 11.3 Hz, H14), 5.32 (1H, d, *J* = 11.3 Hz, H15a), 5.13 (1H, d, *J* = 18.2 Hz, H15b), 4.44 (1H, d, *J* = 9.2 Hz, H8a), 4.00 (1H, ddd, *J* = 11.5, 9.3, 4.3 Hz, H11), 3.77 (1H, d, *J* = 9.2 Hz, H8b), 2.16 (1H, d, *J* = 9.3 Hz, H10), 2.13-2.06 (3H, m, H12a, H3a, H4a), 1.86 (1H, m, H2), 1.77 (1H, m, H4b), 1.49 (2H, m, H12b, H3b), 1.18 (3H, s, H7), 0.97 (3H, d, *J* = 7.2 Hz, H1), 0.96 (9H, t, *J* = 7.9 Hz, $SiCH_2C\underline{H}_3$), 0.61 (6H, q, *J* = 7.9 Hz, $Si\underline{C}H_2CH_3$); **^{13}C NMR** (125 MHz, $CDCl_3$) δ_C 176.7, 139.2, 113.6, 85.0, 74.5, 65.7, 55.9, 52.7, 47.2, 47.0, 41.9, 37.6, 29.0, 22.5, 20.7, 6.8, 5.0; **HRMS** (ES^+) calcd for $C_{21}H_{40}O_4NSi$ $[M+NH_4]^+$ 398.2722, found 398.2723.

A combination of nOe and *J*-based NMR analysis was used to determine the relative stereochemistry between C6, C10 and C11 for **16**. Irradiation of H11 showed nOe contacts with H14 and H8a, while irradiation of H7 showed nOe contact with H8b. Combined with the large vicinal coupling constant ^3J(H10-H11), the arrangement shown in Figure 4 was inferred.

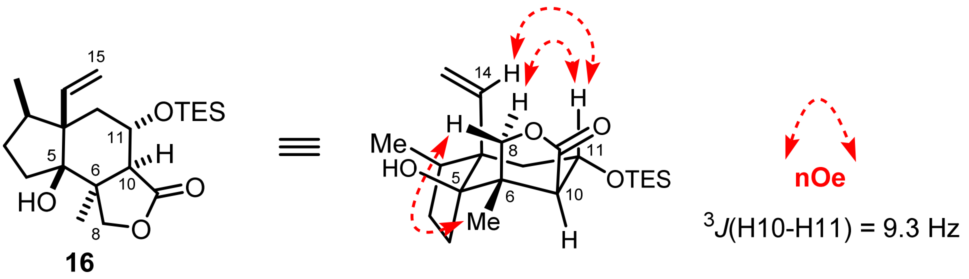

Figure 4. Configurational analysis for TES-tricycle **16**.

Diol 17

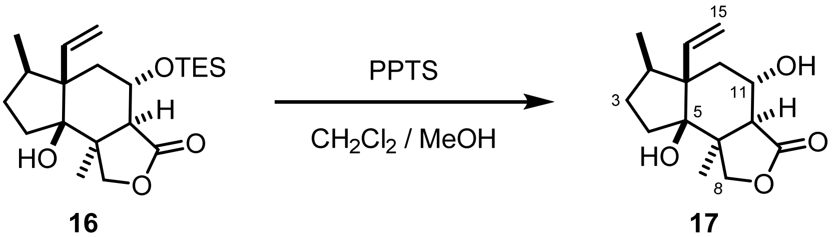

To a stirred solution of TES-tricycle **16** (151 mg, 397 mmol) in CH_2Cl_2 / MeOH (1:2, 6 mL) was added PPTS (10.0 mg, 39.7 mmol). The reaction mixture was stirred for 2 h before being quenched with $NaHCO_3$ (5 mL) and H_2O (5 mL). The reaction mixture was extracted with CH_2Cl_2 (2 × 25 mL), and the combined organic extracts were dried ($MgSO_4$) and concentrated *in vacuo*. Purification by flash column chromatography (EtOAc / 40-60 PE, 1:1) afforded diol **17** (93.0 mg, 88%) as a white solid, which was recrystallized (Et_2O) to provide colorless needles.

R_f 0.31 (EtOAc / 40-60 PE, 1:1); **m.p.** 173-177 °C; **v_{max}** (thin film) / cm^{-1} 3447, 2961, 1760, 1461, 1362, 1173, 1108, 1035, 990, 914, 705; **^{1}H NMR** (500 MHz, $CDCl_3$) δ_H 6.25 (1H, dd, J = 18.3, 11.3 Hz, H14), 5.35 (1H, d, J = 11.3 Hz, H15a), 5.21 (1H, d, J = 18.3 Hz, H15b), 4.51 (1H, d, J = 9.3 Hz, H8a), 4.07 (1H, m, H11), 3.87 (1H, d, J = 9.3 Hz, H8b), 2.56 (1H, br s, OH), 2.29 (1H, dd, J = 13.9, 4.0 Hz, H12a), 2.13-2.08 (3H, m, H12b, H3a, H4a), 1.91 (1H, m, H2), 1.82 (1H, m, H4b), 1.51 (1H, m, H3b), 1.26 (3H, s, H7), 1.01 (3H, d, J = 7.3 Hz, H1); **^{13}C NMR** (125 MHz, $CDCl_3$) δ_C 178.8, 138.2, 114.6, 85.3, 75.8, 65.3, 55.9, 52.7, 47.1, 46.9, 39.3, 37.4, 29.0, 23.0, 20.6; **HRMS** (ES^+) calcd for $C_{15}H_{23}O_4$ $[M+H]^+$ 267.1591, found 267.1594.

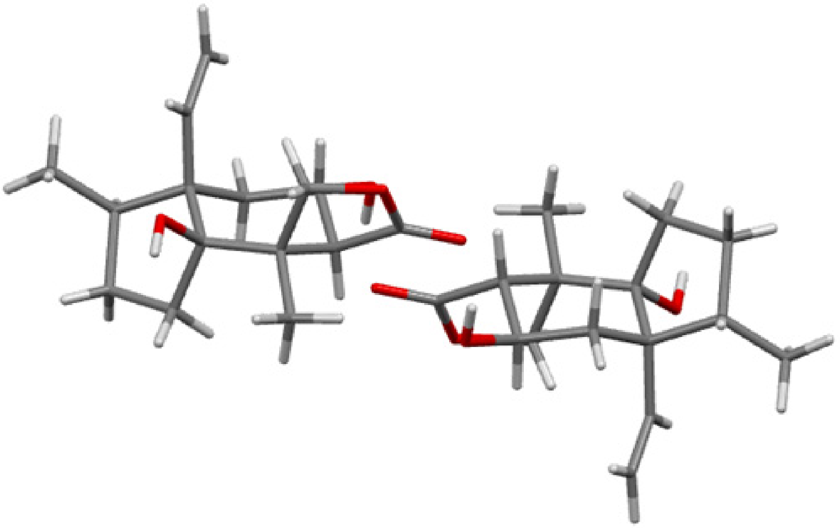

Figure 5. X-ray crystal structure for **17**.[8]

Ketone 2 & Hydroxy-ketone 18

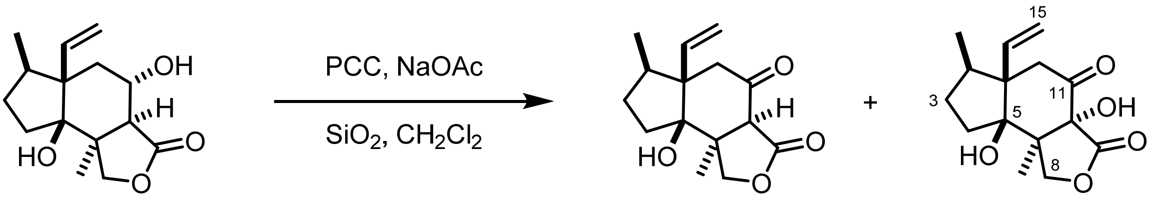

17 **2** **18**

To a stirred solution of alcohol **17** (40.0 mg, 150 µmol) in CH_2Cl_2 (5 mL) was added silica gel (50.0 mg), NaOAc (24.6 mg, 300 µmol) and PCC (64.8 mg, 300 µmol). The reaction mixture was stirred for 16 h before being filtered through a pad of silica (wash with EtOAc, 20 mL) and concentrated *in vacuo*. Purification by flash column chromatography (EtOAc / 40-60 PE, 1:3 → 1:1) afforded ketone **2** (32.4 mg, 81%) as colorless needles together with hydroxy-ketone **18** (4.3 mg, 11%).

18: $\mathbf{R_f}$ 0.30 (EtOAc / 40-60 PE, 1:1); **m.p.** 168-172 °C; $\mathbf{v_{max}}$ (thin film) / cm^{-1} 3513, 2919, 2850, 1783, 1713, 1457, 1369, 1278, 1164, 1126, 1007, 925; **^{1}H NMR** (500 MHz, $CDCl_3$) δ_H 6.20 (1H, dd, *J* = 17.9, 11.1 Hz, H14), 5.37 (1H, d, *J* = 11.1 Hz, H15a), 5.18 (1H, d, *J* = 17.9 Hz, H15b), 4.68 (1H, d, *J* = 9.7 Hz, H8a), 3.98 (1H, d, *J* = 9.7 Hz, H8b), 3.02 (1H, s, H10), 2.91 (1H, d, *J* = 13.8 Hz, H12a), 2.37 (1H, d, *J* = 13.8 Hz, H12b), 2.03-1.96 (3H, m, H3a, 2 × H4), 1.85 (1H, m, H2), 1.59 (1H, m, H3b), 1.36 (3H, s, H7), 0.96 (3H, d, *J* = 7.1 Hz, H1); **^{13}C NMR** (125 MHz, $CDCl_3$) δ_C 200.4, 170.7,

[8] CCDC 993662 contains the supplementary crystallographic data for alcohol **17**. This data can be obtained free of charge from The Cambridge Crystallographic Data Centre via www.ccdc.cam.ac.uk/data_request/cif.

137.8, 116.0, 84.2, 75.5, 61.2, 55.6, 50.0, 46.5, 46.0, 38.4, 29.3, 23.4, 17.4; **HRMS** (ES^+) calcd for $C_{15}H_{21}O_4$ $[M+H]^+$ 265.1434, found 265.1435.

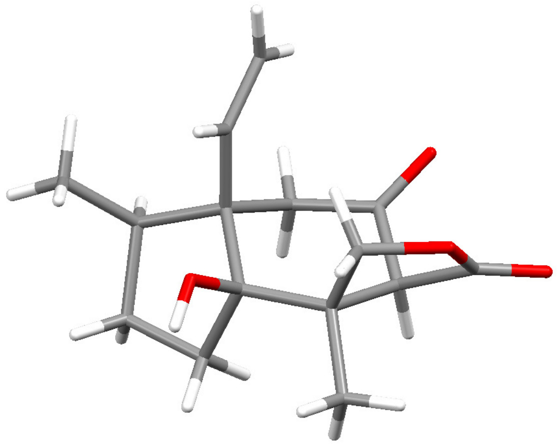

Figure 6. X-ray crystal structure of ketone **2**.[9]

Hydroxy-ketone 18

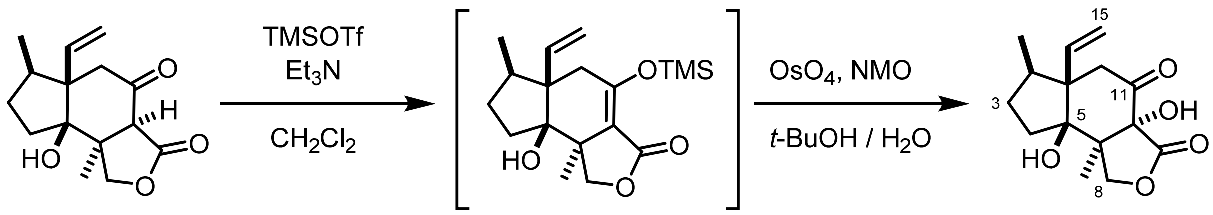

To a stirred solution of ketone **2** (32.4 mg, 123 μmol) and Et_3N (300 μL, 2.15 mmol) in THF (5 mL) at −78 °C was added TMSOTf (300 μL, 1.66 mmol). The reaction mixture was stirred for 1 h before being quenched with $NaHCO_3$ (3 mL) and warmed to RT. The reaction mixture was extracted with CH_2Cl_2 (2 × 15 mL) and the combined organic extracts were dried ($MgSO_4$) and concentrated *in vacuo* to afford crude TMS enol ether **2a** as a yellow oil which was used immediately without further purification.

^{1}H NMR (500 MHz, $CDCl_3$) δ_H 6.20 (1H, dd, J = 17.9, 11.1 Hz, H14), 5.27 (1H, d, J = 11.1 Hz, H15a), 5.10 (1H, d, J = 17.9 Hz, H15b), 4.62 (1H, d, J = 9.7 Hz, H8a), 3.91 (1H, d, J = 9.7 Hz, H8b), 3.02 (1H, s, OH), 2.83 (1H, d, J = 13.8 Hz, H12a), 2.36 (1H, d, J = 13.8 Hz, H12b), 2.12-1.54 (5H, m, H2, 2 × H3, 2 × H4), 1.23 (3H, s, H7), 0.93 (3H, d, J = 7.1 Hz, H1), 0.12 (9H, s, Si(C<u>H</u>$_3$)$_3$).

[9] CCDC 993663 contains the supplementary crystallographic data for ketone **2**. This data can be obtained free of charge from The Cambridge Crystallographic Data Centre via www.ccdc.cam.ac.uk/data_request/cif.

The crude TMS enol ether **2a** was taken up in *t*-BuOH / H_2O (1:1, 2 mL) and NMO (50 wt% in H_2O, 200 µL, 100 µmol) and OsO_4 (4 wt% in H_2O, 200 µL, 5.0 µmol) was added. The reaction mixture was stirred for 1 h before being quenched with $Na_2S_2O_3$ (2 mL). The mixture was stirred for 1 h before being diluted with H_2O (5 mL) and extracted with EtOAc (3 × 10 mL). The combined organic extracts were dried ($MgSO_4$) and concentrated *in vacuo*. Purification by flash chromatography (EtOAc / 40-60 PE, 3:7) afforded hydroxy-ketone **18** (36.6 mg, 99% over 2 steps) as a colorless oil.

R_f 0.65 (EtOAc / 40-60 PE, 1:1); **v_{max}** (thin film) / cm^{-1} 3449, 2964, 1780, 1721, 1373, 1180, 1110, 1014; **^{1}H NMR** (500 MHz, $CDCl_3$) δ_H 6.20 (1H, dd, *J* = 17.6, 11.0 Hz, H14), 5.36 (1H, d, *J* = 11.0 Hz, H15a), 5.13 (1H, d, *J* = 17.6 Hz, H15b), 4.72 (1H, d, *J* = 9.9 Hz, H8a), 4.24 (1H, br s, OH), 4.12 (1H, d, *J* = 9.9 Hz, H8b), 2.95 (1H, d, *J* = 13.0 Hz, H12a), 2.46 (1H, d, *J* = 13.0 Hz, H12b), 1.86-1.59 (5H, m, H2, 2 × H3, 2 × H4), 1.35 (3H, s, H7), 0.85 (3H, d, *J* = 6.8 Hz, H1); **^{13}C NMR** (125 MHz, $CDCl_3$) δ_C 203.9, 172.4, 138.3, 114.1, 84.5, 82.7, 75.0, 56.4, 53.7, 45.1, 41.7, 38.3, 29.9, 17.9, 14.9; **HRMS** (ES^+) calcd for $C_{15}H_{21}O_5$ $[M+H]^+$ 281.1384, found 281.1384.

Triol 18a

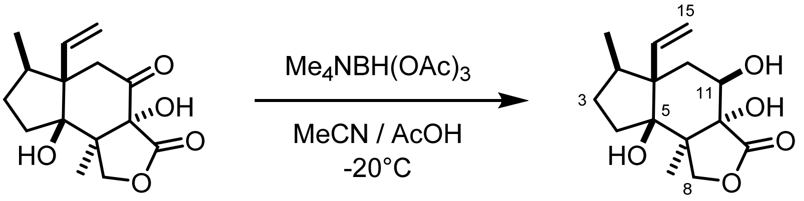

18 **18a**

To a stirred solution of $Me_4NBH(OAc)_3$ (93.8 mg, 356 µmol) in MeCN / AcOH (3:1, 4 mL) at −20 °C was added a solution of hydroxy-ketone **18** (10.0 mg, 35.6 µmol) in MeCN (2 mL). The reaction mixture was stirred for 72 h before being decanted into a stirred solution of $NaHCO_3$ (10 mL) and Na^+ / K^+ tartrate (10 mL) at 0 °C. The mixture was stirred for 1 h at 0 °C before being extracted with EtOAc (3 × 10 mL). The combined organic extracts were dried ($MgSO_4$) and concentrated *in vacuo*. Purification by flash chromatography (EtOAc / 40-60 PE, 1:1) afforded triol **18a** (8.8 mg, 87%) as a colorless oil.

R_f 0.44 (EtOAc / 40-60 PE, 1:1); **v_{max}** (thin film) / cm^{-1} 3474, 2931, 1762, 1456, 1377, 1261, 1180, 1093, 1015, 974, 911, 814; **^{1}H NMR** (500 MHz, $CDCl_3$) δ_H 6.38 (1H, dd, *J* = 18.0, 11.0 Hz, H14), 5.38 (1H, d, *J* = 11.0 Hz, H15a), 5.25 (1H, d, *J* = 18.0 Hz, H15b), 4.49 (1H, d, *J* = 9.5 Hz, H8a), 4.03 (1H, m, H11), 3.92 (1H, d, *J* = 9.5 Hz, H8b), 3.03 (1H, br s, OH), 2.81 (1H, d, *J* = 6.3 Hz, OH), 2.14-1.93 (4H, m, H2, H3a, H4a, H12a), 1.81 (1H, dd, *J* = 14.7, 3.6 Hz, H12b), 1.76 (1H, m, H4b), 1.53 (1H, m, H3b), 1.27 (3H, s, H7), 0.92 (3H, d, *J* = 7.1 Hz, H1); **^{13}C NMR** (125 MHz, $CDCl_3$) δ_C 178.2, 141.1, 114.8, 84.7, 79.5, 75.0, 72.9, 52.0, 49.9, 45.4, 38.4, 33.9, 29.8, 17.8, 17.4; **HRMS** (ES^+) calcd for $C_{15}H_{26}O_5N$ $[M+NH_4]^+$ 300.1805, found 300.1810.

TES ether 20

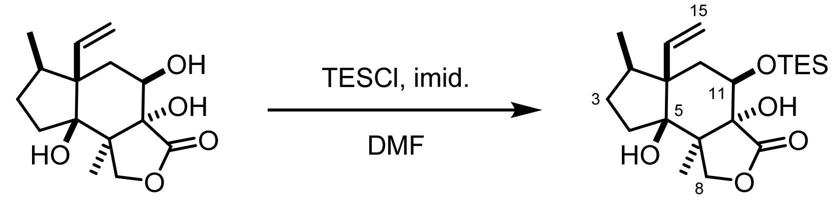

18a **20**

To a stirred solution of triol **18a** (7.0 mg, 25 µmol) and imidazole (3.4 mg, 50 µmol) in DMF (1 mL) at 0 °C was added TESCl (10.0 µl, 59.6 µmol). The reaction mixture was stirred at RT for 1.5 h before being quenched with H_2O (5 mL). The mixture was extracted with Et_2O (2 × 10 mL) and the combined organic extracts were washed with H_2O (2 x 5 mL), dried ($MgSO_4$) and concentrated *in vacuo*. Purification by flash chromatography (EtOAc / 40-60 PE, 1:4 → 2:3) afforded TES ether **20** (9.4 mg, 96%) as a colorless oil.

R_f 0.24 (EtOAc / 40-60 PE, 1:4); **v_{max}** (thin film) / cm^{-1} 3479, 2960, 1744, 1183, 1135, 1086, 992, 974, 903, 869, 739; **^{1}H NMR** (500 MHz, $CDCl_3$) δ_H 6.15 (1H, dd, *J* = 17.9, 11.2 Hz, H14), 5.27 (1H, d, *J* = 11.2 Hz, H15a), 5.16 (1H, d, *J* = 17.9 Hz, H15b), 4.51 (1H, d, *J* = 9.5 Hz, H8a), 4.07 (1H, dd,*J* = 10.5, 3.8 Hz, H11), 3.81 (1H, d, *J* = 9.5 Hz, H8b), 2.97 (1H, br s, OH), 2.01 (2H, m, H12a, H2), 1.89 (2H, m, H3a, H4a), 1.75 (1H, dd, *J* = 13.0, 6.7 Hz, H4b), 1.62 (1H, br s, OH), 1.59 (1H, dd, *J* = 14.5, 3.8 Hz, H12b), 1.53 (1H, m, H3b), 1.21 (3H, s, H7), 0.99 (9H, t, *J* = 8.0 Hz, $SiCH_2C\underline{H}_3$), 0.90 (3H, d, *J* = 7.0 Hz, H1), 0.64 (6H, q, *J* = 8.0 Hz, $SiC\underline{H}_2CH_3$); **^{13}C NMR** (125 MHz, $CDCl_3$) δ_C 176.9, 139.7, 114.2, 84.7, 80.1, 74.5, 73.8, 51.9, 50.6, 45.3, 38.8, 35.8, 30.1, 17.9, 16.5, 6.8, 4.8; **HRMS** (ES^+) calcd for $C_{21}H_{37}O_5Si$ $[M+H]^+$ 397.2405, found 397.2405.

Tetra-ol 20a

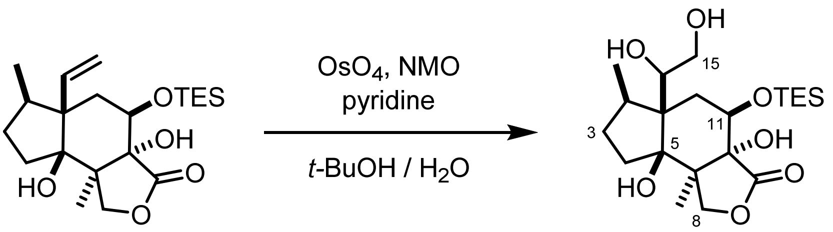

20 **20a**

To a stirred solution of TES ether **20** (9.0 mg, 23 µmol) in *t*-BuOH / H_2O (1:1, 2 mL) was added NMO (50 wt% in H_2O, 500 µL, 250 µmol), OsO_4 (4 wt% in H_2O, 250 µL, 10.0 µmol) and pyridine (100 µL, 1.24 mmol). The mixture was stirred for 5 h before being quenched with $Na_2S_2O_3$ (5 mL) and diluted with CH_2Cl_2 (5 mL). The mixture was stirred vigorously for 2 h before being extracted with CH_2Cl_2 (2 × 10 mL). The combined organic extracts were then dried ($MgSO_4$) and concentrated *in vacuo*. Purification by flash chromatography (EtOAc / 40-60 PE, 1:1 → 7:3) afforded tetra-ol **20a** (9.3 mg, 95%, >19:1 d.r., undefined configuration at C14) as a colorless oil.

R_f 0.26 (EtOAc / 40-60 PE, 1:1); **v_{max}** (thin film) / cm^{-1} 3438, 2955, 2877, 1769, 1458, 1241, 1181, 1108, 1010, 978, 891, 817, 746; **^{1}H NMR** (500 MHz, $CDCl_3$) δ_H 4.76 (1H, d, *J* = 9.9 Hz, H8a), 4.26 (1H, br s, OH), 4.08 (1H, dd, *J* = 12.1, 4.7 Hz, H11), 3.96 (1H, d, *J* = 7.4 Hz, H14), 3.91 (1H, d, *J* = 9.9 Hz, H8b), 3.86 (2H, m, 2 × H15), 3.08 (1H, br s, OH), 2.07 (1H, m, H2), 1.94-1.62 (5H, m, 2 × H3, 2 × H4, H12a), 1.42 (1H, dd, *J* = 14.3, 4.7 Hz, H12b), 1.22 (3H, s, H7), 1.19 (3H, d, *J* = 7.0 Hz, H1), 0.98 (9H, t, *J* = 7.9 Hz, $SiCH_2C\underline{H}_3$), 0.64 (6H, m, $SiC\underline{H}_2CH_3$); **^{13}C NMR** (125 MHz, $CDCl_3$) δ_C 176.5, 86.2, 79.8, 75.5, 75.2, 74.4, 63.7, 51.2, 50.9, 44.7, 39.5, 34.3, 30.9, 17.8, 15.3, 6.6, 4.7; **HRMS** (ES^+) calcd for $C_{21}H_{39}O_7Si$ $[M+H]^+$ 431.2460, found 431.2460.

Keto-Lactone 20b

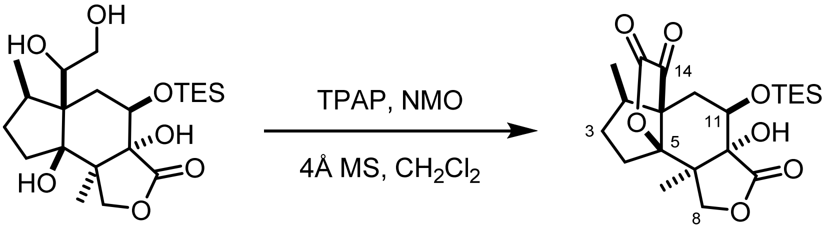

20a **20b**

To a stirred solution of tetraol **20a** (9.3 mg, 22 µmol) in CH_2Cl_2 (2 mL) over 4Å molecular sieves (*ca.* 10 mg) was added TPAP (7.6 mg, 22 µmol) and NMO (50 wt% in H_2O, 200 µL, 100 µmol). The reaction mixture was stirred for 1 h before being filtered through a pad of silica gel (wash with EtOAc,

10 mL) and concentrated *in vacuo*. Purification by flash chromatography (EtOAc / 40-60 PE, 1:4 to 3:7) afforded keto-lactone **20b** (8.1 mg, 88%) as a colorless oil.

R$_f$ 0.54 (EtOAc / 40-60 PE, 1:1); **v$_{max}$** (thin film) / cm^{-1} 3445, 2957, 2879, 1784, 1458, 1246, 1174, 1087, 1019, 962, 811, 734; **^{1}H NMR** (500 MHz, $CDCl_3$) δ_H 4.23 (1H, d, *J* = 9.0 Hz, H8a), 3.93 (1H, d, *J* = 9.0 Hz, H8b), 3.91 (1H, dd, *J* = 4.7, 2.3 Hz, H11), 2.83 (1H, br s, OH), 2.59 (1H, dd, *J* = 14.5, 4.7 Hz, H12a), 2.39 (1H, m, H4a), 2.34 (1H, m, H2), 2.09 (1H, dd, *J* = 14.5, 2.3 Hz, H12b), 2.02 (2H, m, H3a, H4b), 1.39 (3H, s, H7), 1.29 (1H, m, H3b), 0.95 (9H, t, *J* = 7.9 Hz, $SiCH_2C\underline{H}_3$), 0.94 (3H, m, H1), 0.63 (6H, m, $SiC\underline{H}_2CH_3$); **^{13}C NMR** (125 MHz, $CDCl_3$) δ_C 195.0, 177.7, 160.3, 95.4, 78.6, 74.1, 72.0, 55.2, 49.4, 47.8, 36.6, 36.2, 30.8, 19.4, 15.8, 6.5, 4.1; **HRMS** (ES$^+$) calcd for $C_{21}H_{36}O_7NSi$ $[M+NH_4]^+$ 442.2256, found 442.2255.

(±)-Jiadifenolide (1)

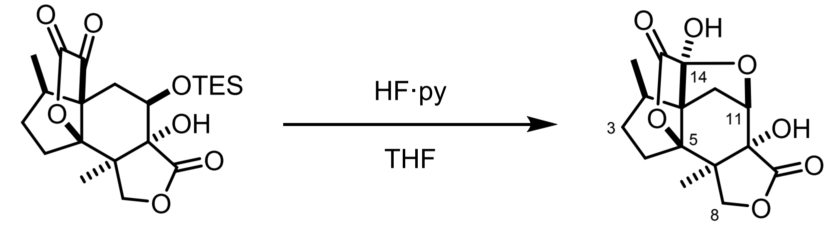

20b — **1**: *Jiadifenolide*

To a stirred solution of keto-lactone **20b** (8.0 mg, 19 µmol) in THF (2 mL) was added HF·py (100 µL). The reaction mixture was stirred for 1 h before being cooled to 0 °C and quenched with $NaHCO_3$ (2 mL). The mixture was extracted with CH_2Cl_2 (2 × 10 mL) and the combined organic extracts were dried ($MgSO_4$) and concentrated *in vacuo*. Purification by flash chromatography (EtOAc / 40-60 PE, 1:1 to 4:1) afforded (±)-jiadifenolide **1** (5.0 mg, 86%) as an amorphous white solid.

R$_f$ 0.12 (EtOAc / 40-60 PE, 1:1); **m.p.** 238-245 °C; **v$_{max}$** (thin film) / cm^{-1} 3398, 2957, 1772, 1473, 1404, 1382, 1245, 1206, 1161, 1085, 985, 746; **^{1}H NMR** (500 MHz, CD_3OD) δ_H 4.61 (1H, d, *J* = 9.4 Hz, H8a), 4.42 (1H, d, *J* = 6.0 Hz, H11), 3.80 (1H, d, *J* = 9.4 Hz, H8b), 2.46 (1H, dd, *J* = 12.9, 6.0 Hz, H12a), 2.22 (1H, m, H2), 2.10 (1H, m, H4a), 2.09 (1H, d, *J* = 12.9 Hz, H12b), 2.01 (1H, ddd, *J* = 13.7, 5.6, 1.5 Hz, H4b), 1.89 (1H, m, H3a), 1.28 (1H, m, H3b), 1.23 (3H, s, H7), 1.21 (3H, d, *J* = 7.3 Hz, H1); **^{13}C NMR** (125 MHz, CD_3OD) δ_C 178.2, 173.6, 101.9, 97.4, 79.1, 77.6, 74.6, 58.8, 48.1, 41.6, 35.2, 33.0, 32.1, 19.9, 14.7; **HRMS** (ES$^+$) calcd for $C_{15}H_{22}O_7N$ $[M+NH_4]^+$ 328.1391, found 328.1394.

3. NMR comparison for natural and synthetic jiadifenolide

Table 1. *Tabulated comparison of NMR data for natural and synthetic jiadifenolide*

Atom #	Natural jiadifenolide[a]		Synthetic jiadifenolide	
	^{13}C NMR	^{1}H NMR	^{13}C NMR[b]	^{1}H NMR[c]
**1**	14.7	1.20 (3H, d, 7.1)	14.7	1.21 (3H, d, 7.3)
**2**	41.6	2.21 (1H, qdd, 12.3, 12.3, 7.1)	41.6	2.22 (1H, m)
**3a**	33.0	1.88 (1H, dddd, 12.3, 12.3, 12.3, 4.1)	33.0	1.89 (1H, m)
**3b**		1.27 (1H, dddd, 12.3, 12.3, 12.3, 6.0)		1.28 (1H, m)
**4a**	35.2	2.08 (1H, ddd, 12.9, 12.3, 6.0)	35.2	2.10 (1H, m)
**4b**		2.00 (1H, brdd, 12.9, 4.1)		2.01 (1H, ddd, 14.7, 5.6, 1.5)
**5**	97.4		97.4	
**6**	48.1		48.1	
**7**	19.9	1.22 (3H, s)	19.9	1.23 (3H, s)
**8a**	74.6	4.60 (1H, d, 9.3)	74.6	4.61 (1H, d, 9.4)
**8b**		3.79 (1H, d, 9.3)		3.80 (1H, d, 9.4)
**9**	178.3		178.2	
**10**	77.6		77.6	
**11**	79.1	4.41 (1H, d, 5.8)	79.1	4.42 (1H, d, 6.0)
**12a**	32.1	2.46 (1H, dd, 12.9, 5.8)	32.1	2.46 (1H, dd, 12.9, 6.0)
**12b**		2.09 (1H, d, 12.9)		2.09 (1H, d, 12.9)
**13**	58.8		58.8	
**14**	101.9		101.9	
**15**	173.6		173.6	

[a] M. Kubo, C. Okada, J.-M. Huang, K. Harada, H. Hioki, Y. Fukuyama, *Org. Lett.* **2009**, *11*, 5190.

[b] ^{13}C NMR (125 MHz, CD_3OD): chemical shift (δ_C in ppm). ^{13}C assignments were made using a combination of HMQC / HMBC NMR experiments.

[c] ^{1}H NMR (500 MHz, CD_3OD): chemical shift (δ_H in ppm), multiplicity, (J / Hz). ^{1}H assignments were made using a combination of COSY and HMBC NMR experiments.

4. ^{1}H and ^{13}C NMR Spectra for new compounds

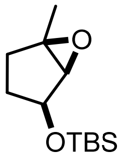

7

^{1}H NMR (500 MHz, $CDCl_3$)

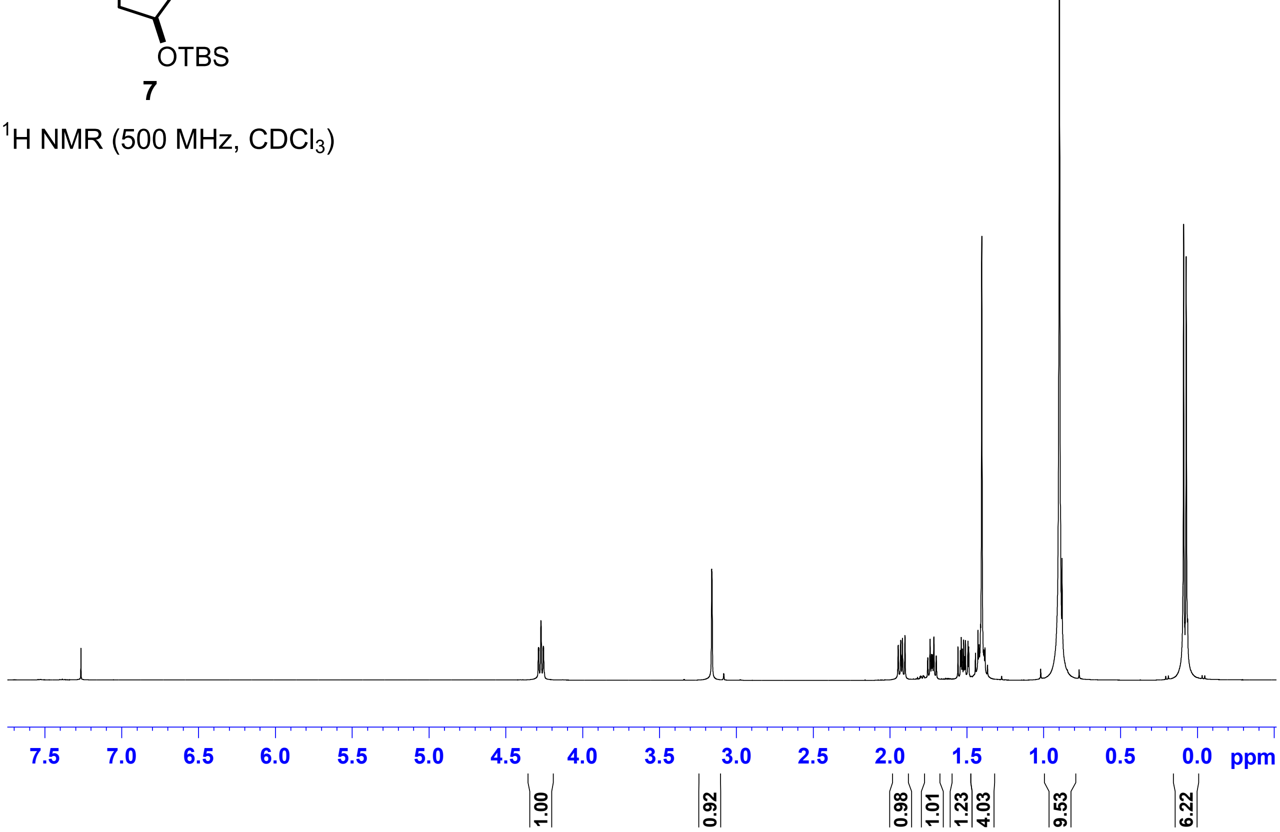

^{13}C NMR (125 MHz, $CDCl_3$)

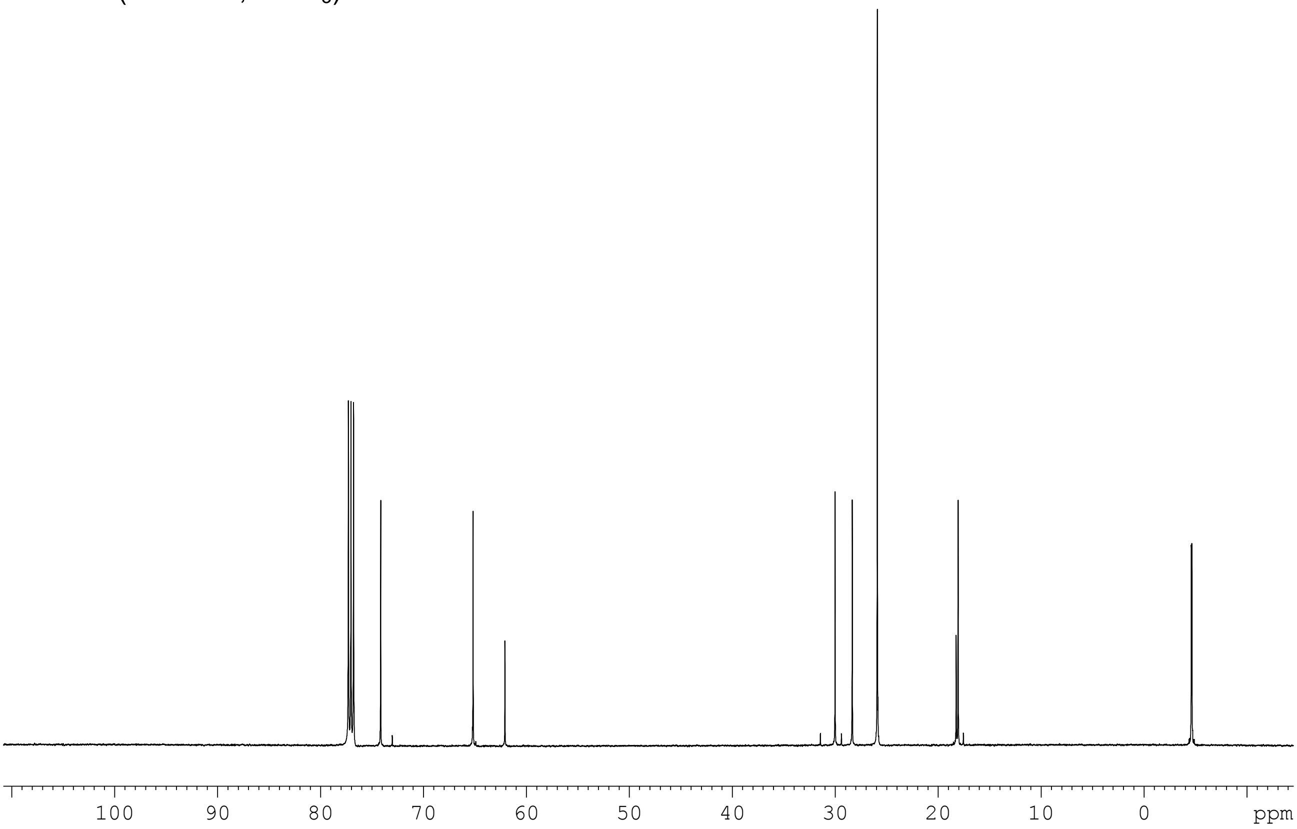

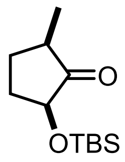

8

^{1}H NMR (500 MHz, $CDCl_3$)

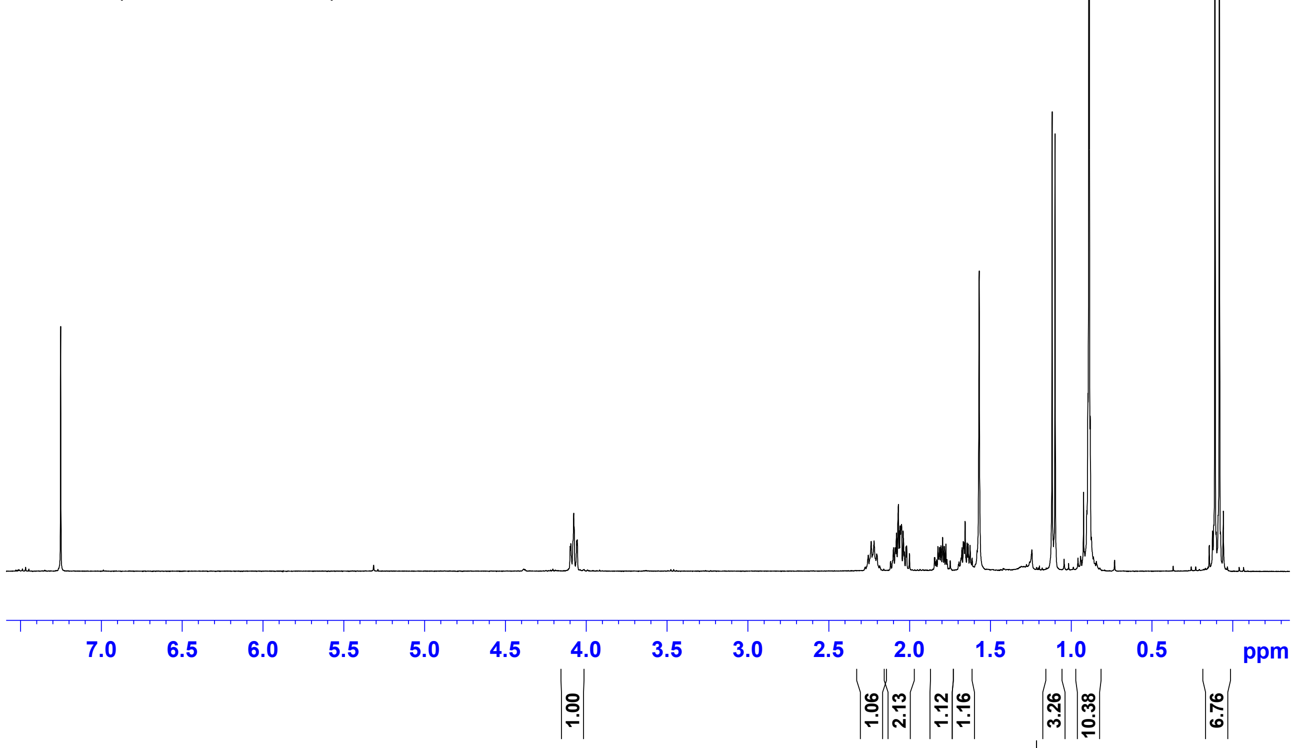

^{13}C NMR (125 MHz, $CDCl_3$)

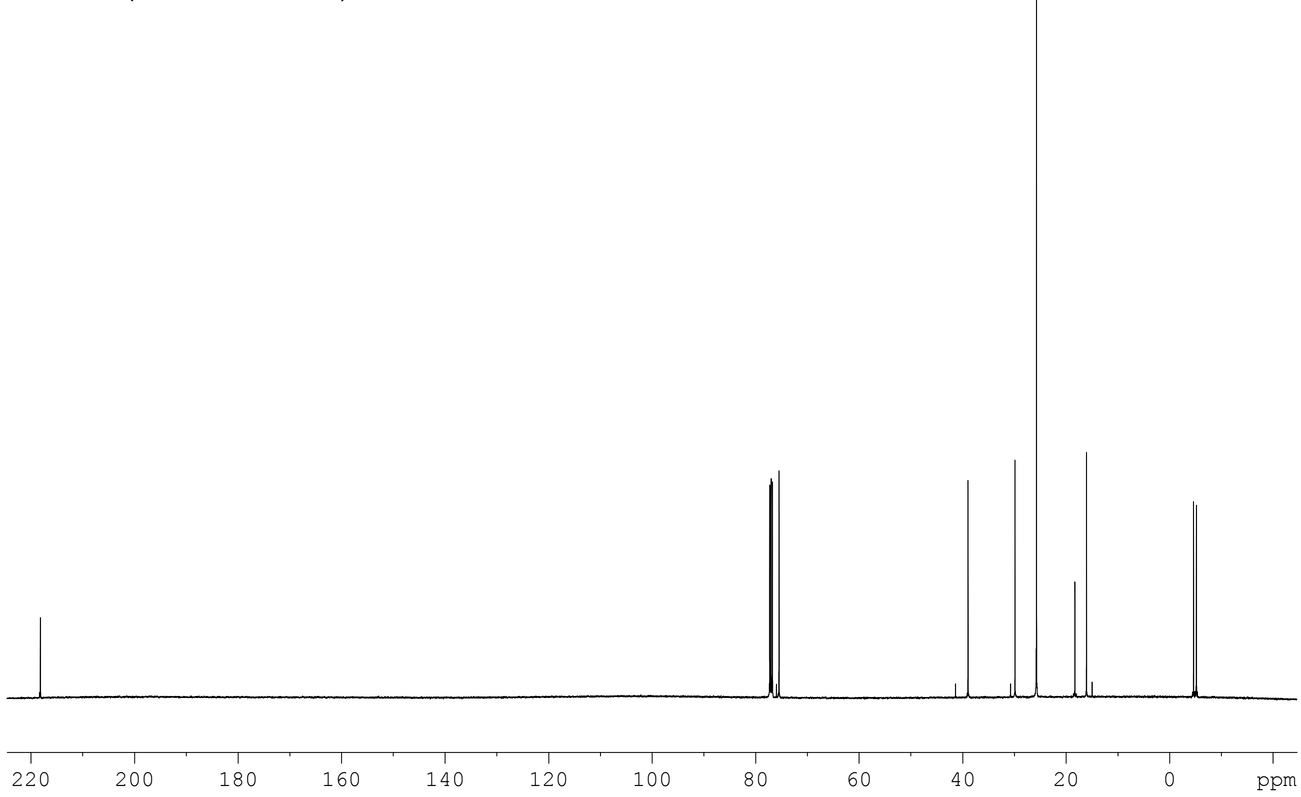

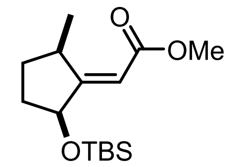

10

^{1}H NMR (500 MHz, $CDCl_3$)

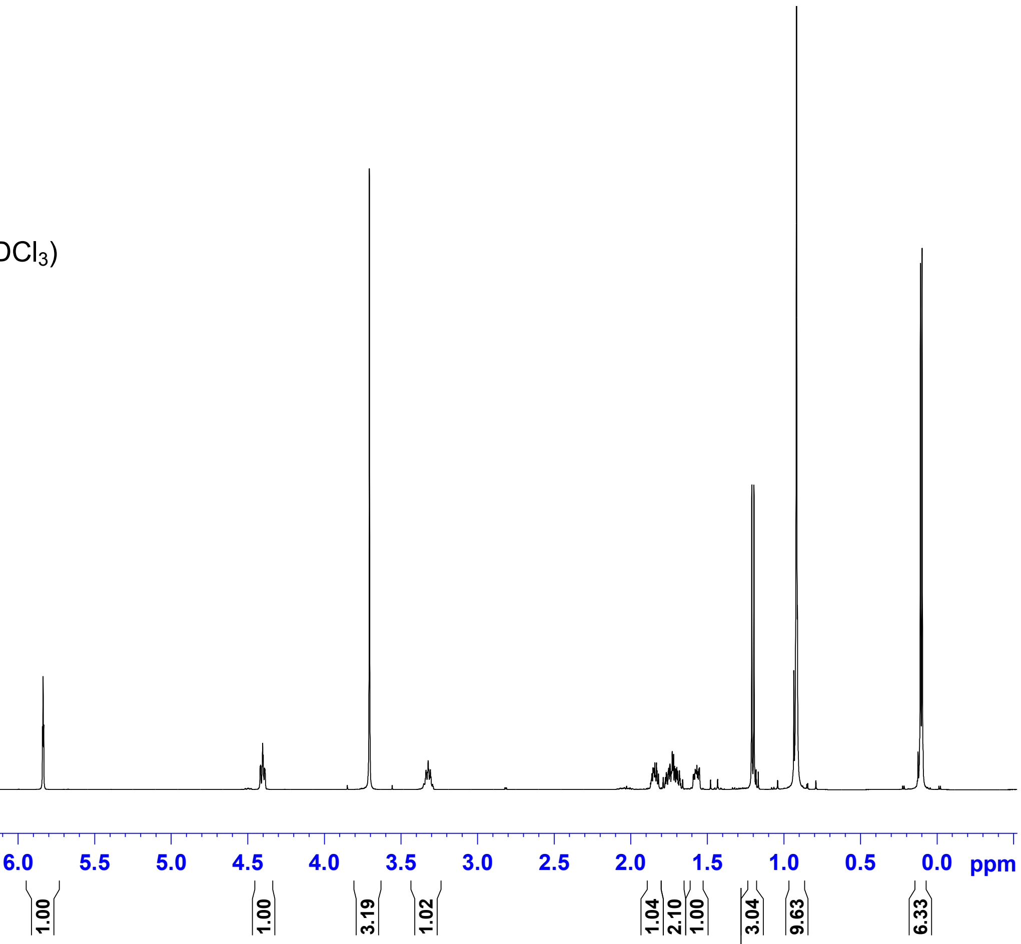

^{13}C NMR (125 MHz, $CDCl_3$)

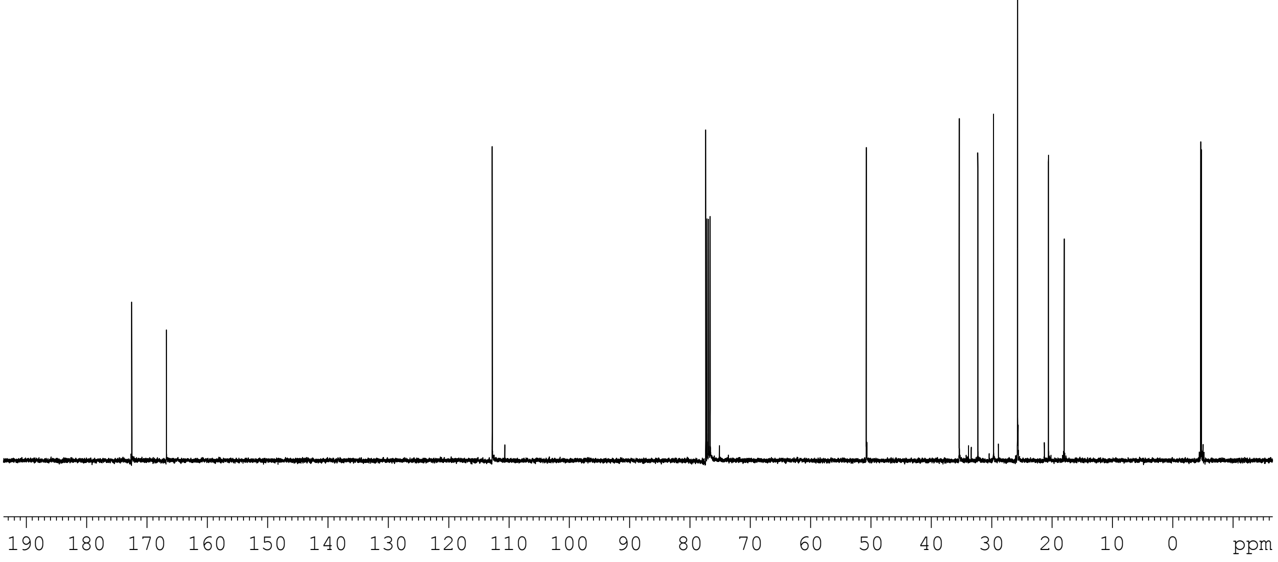

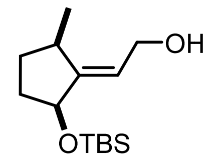

10a

^{1}H NMR (500 MHz, $CDCl_3$)

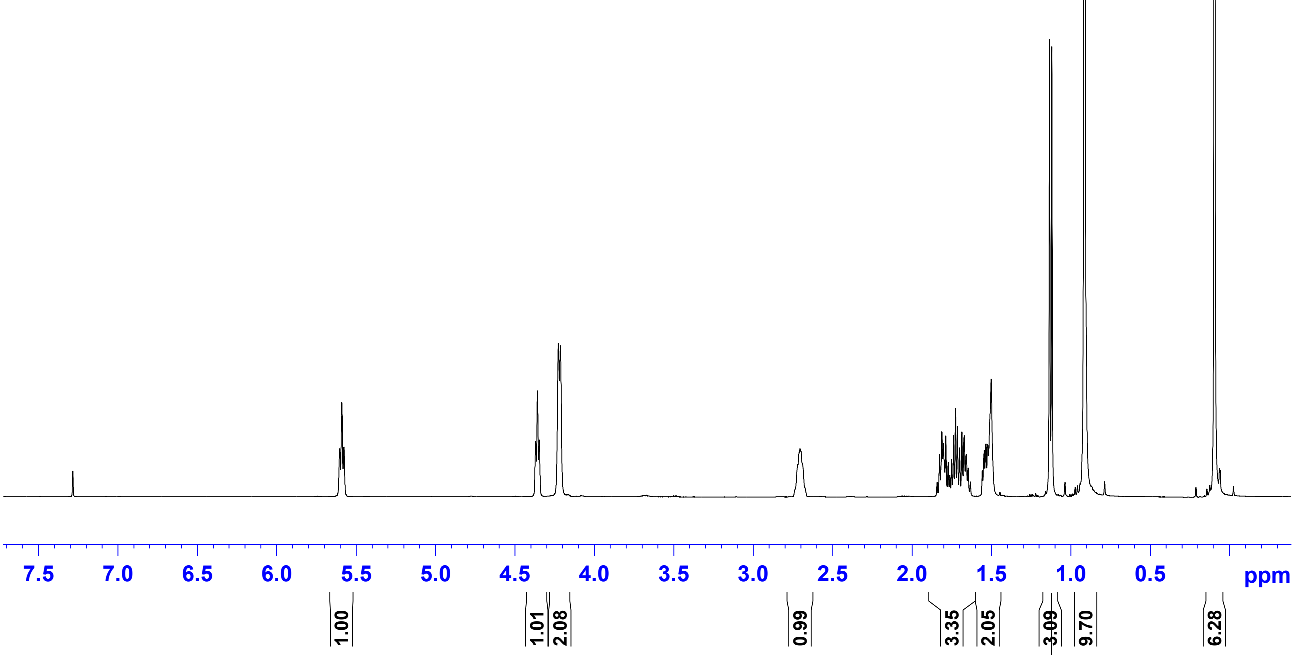

^{13}C NMR (125 MHz, $CDCl_3$)

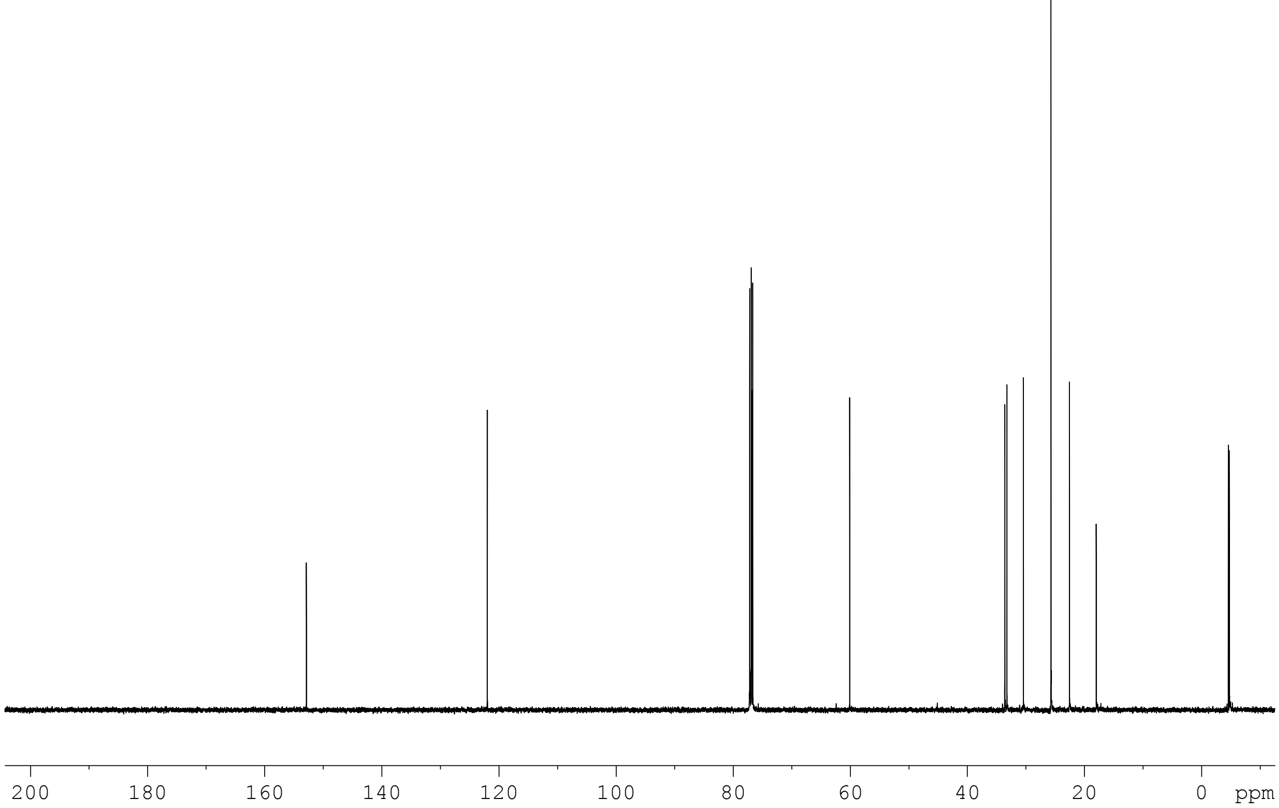

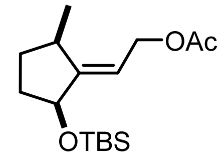

11

^{1}H NMR (500 MHz, $CDCl_3$)

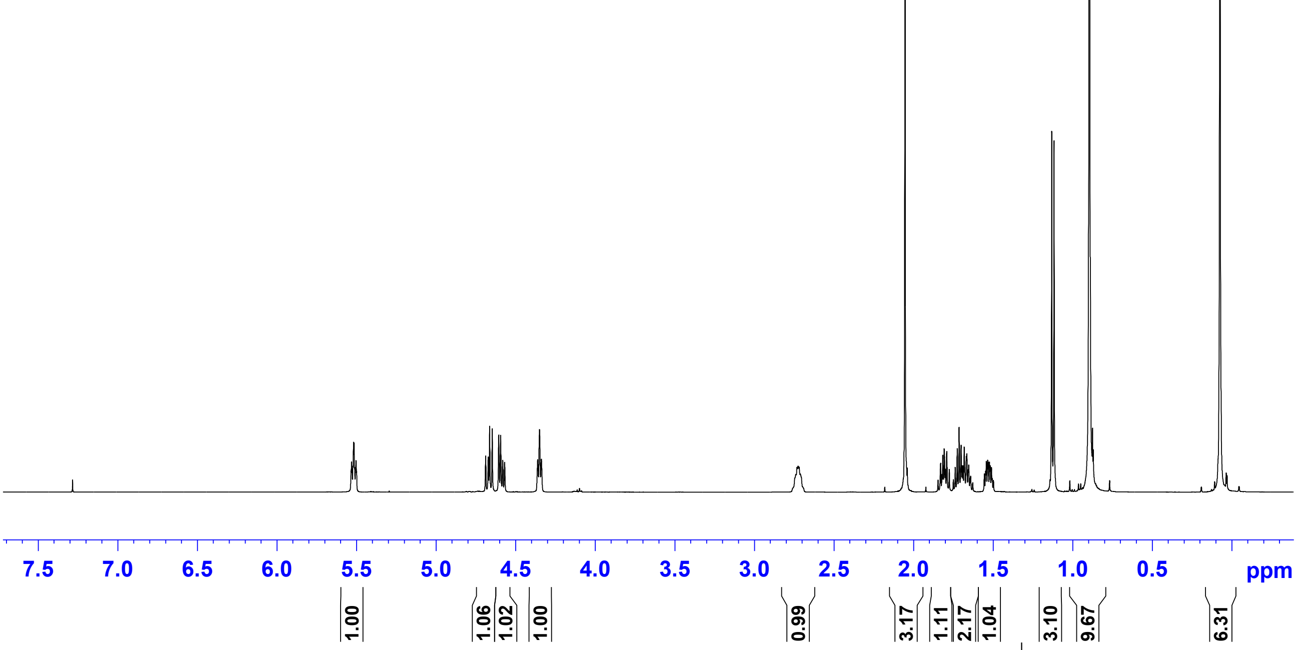

^{13}C NMR (125 MHz, $CDCl_3$)

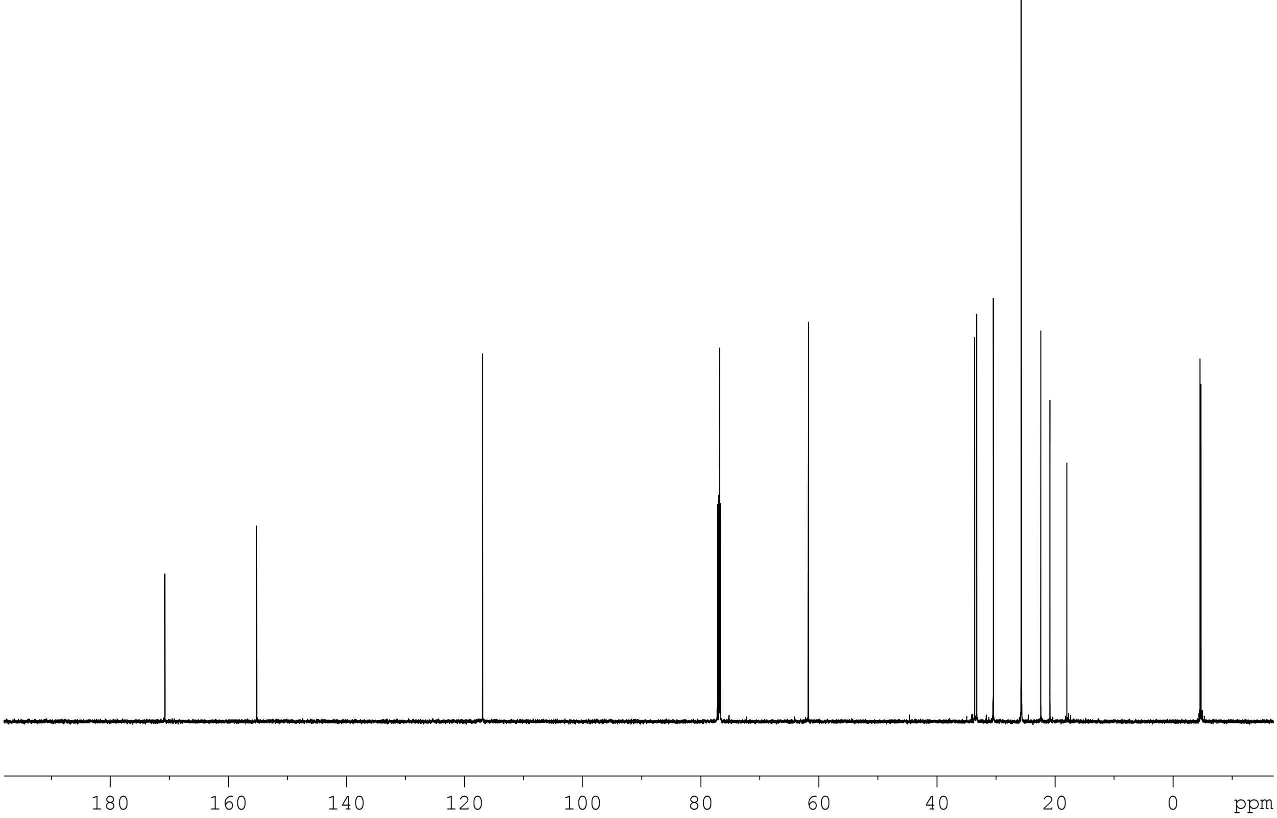

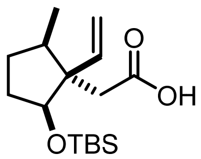

11c

^{1}H NMR (500 MHz, $CDCl_3$)

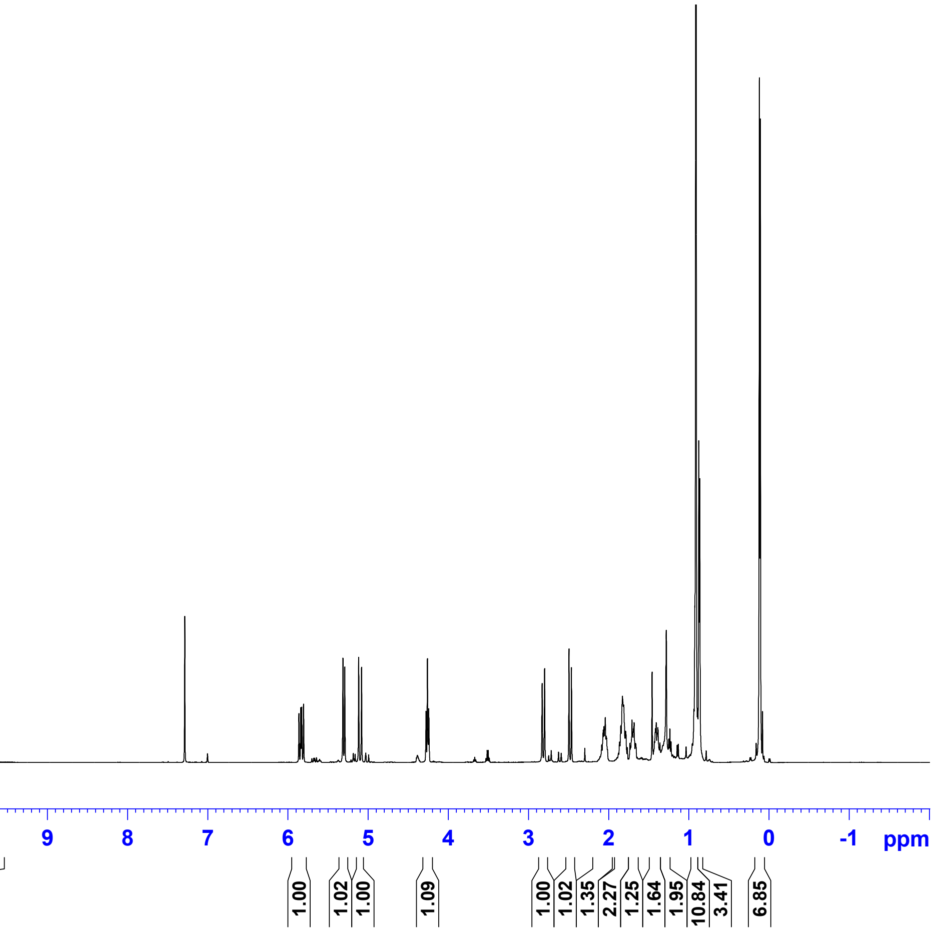

^{13}C NMR (125 MHz, $CDCl_3$)

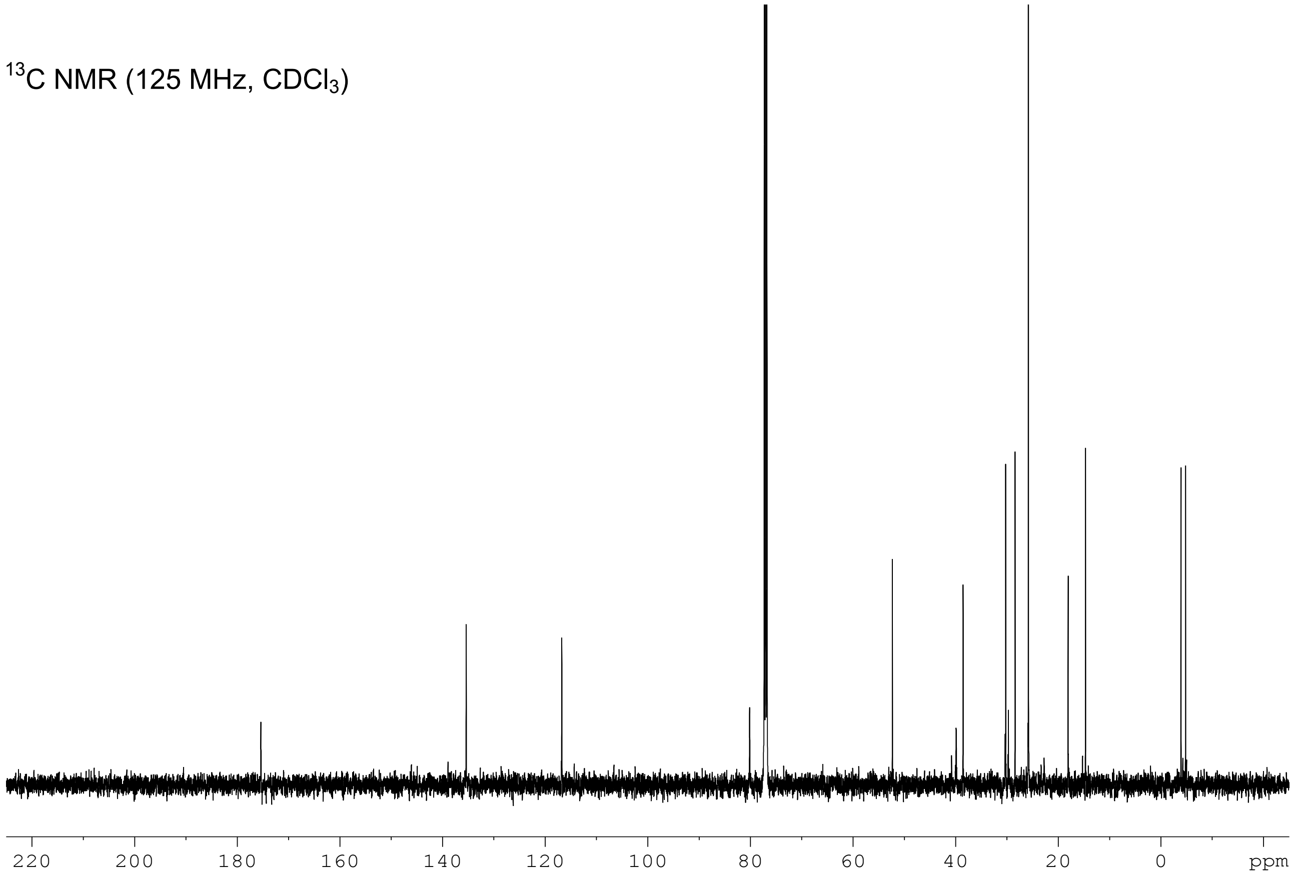

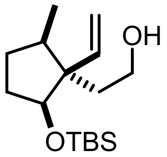

12

^{1}H NMR (500 MHz, $CDCl_3$)

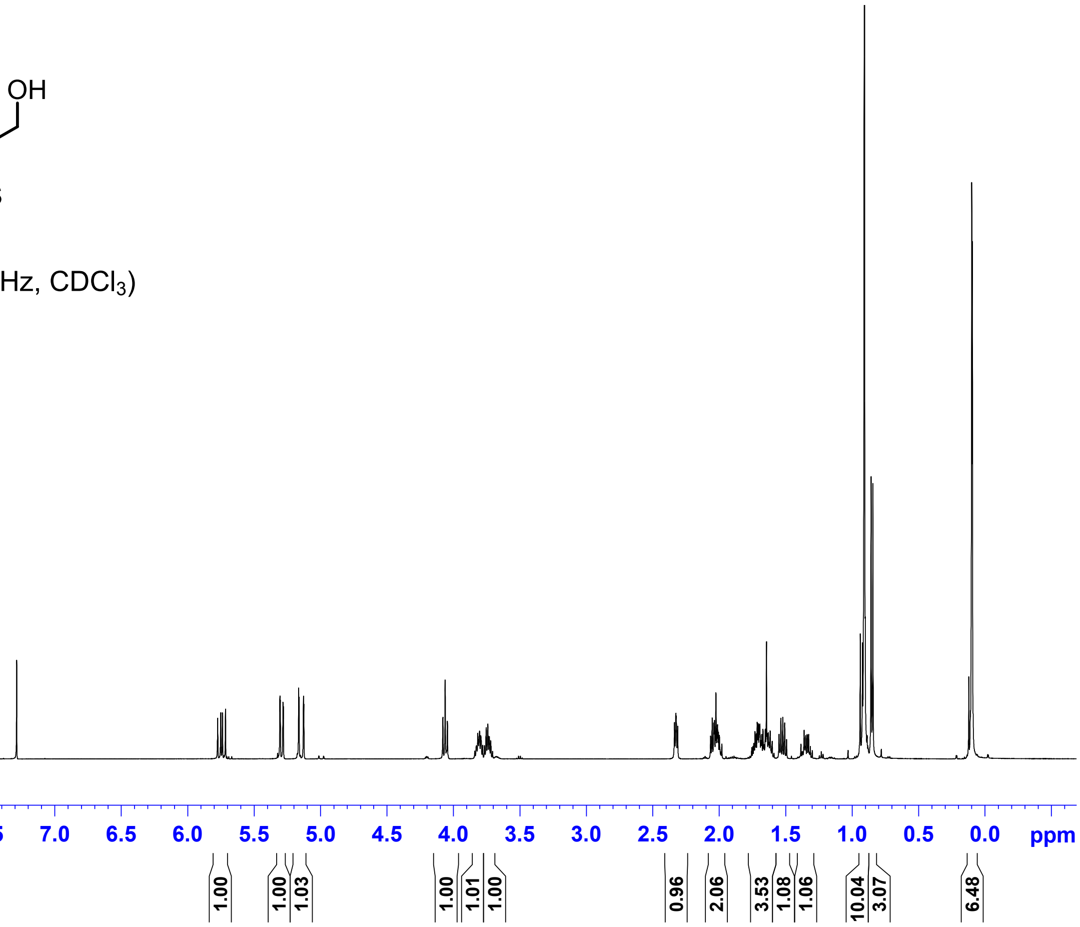

^{13}C NMR (125 MHz, $CDCl_3$)

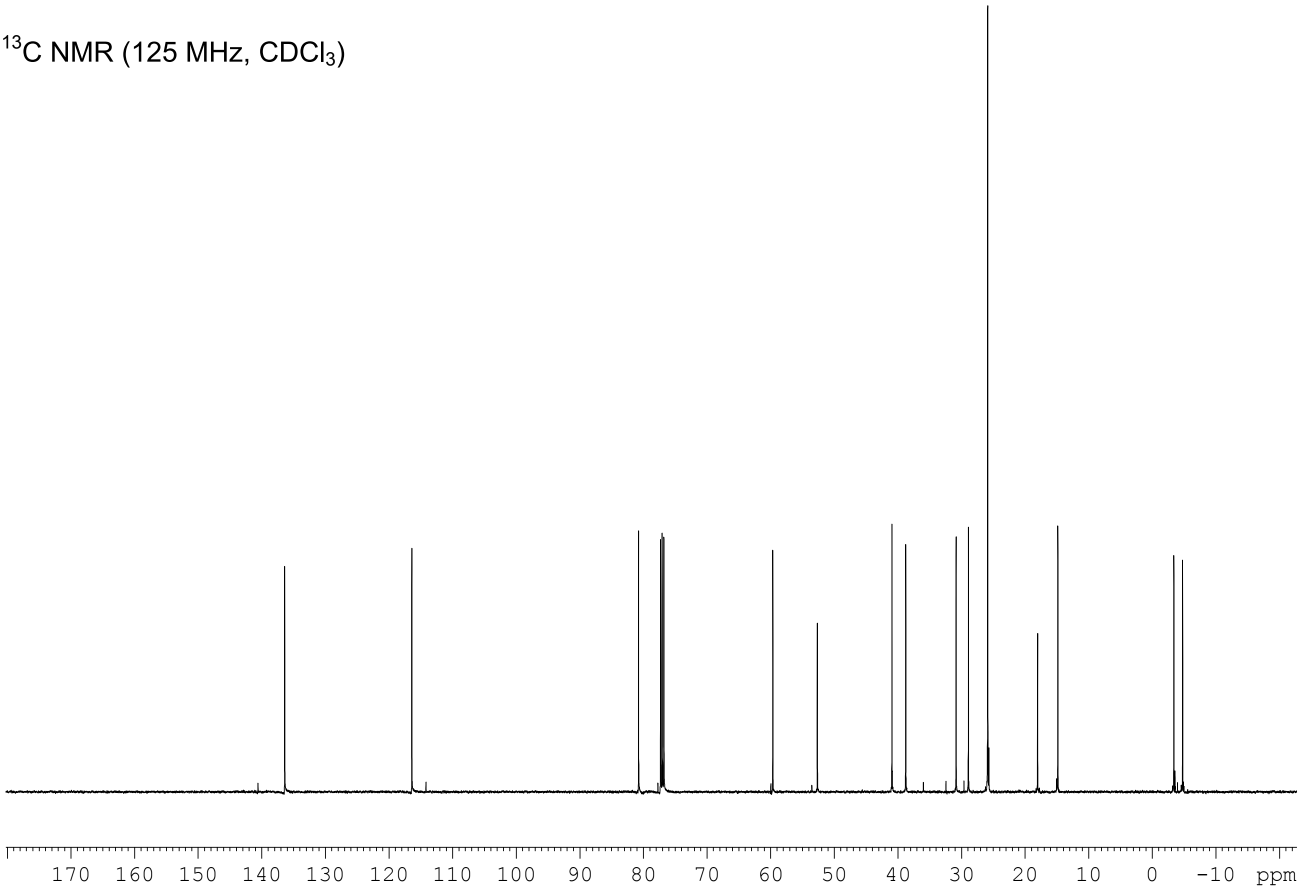

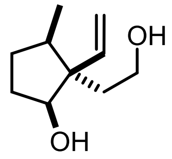

12a

^{1}H NMR (500 MHz, $CDCl_3$)

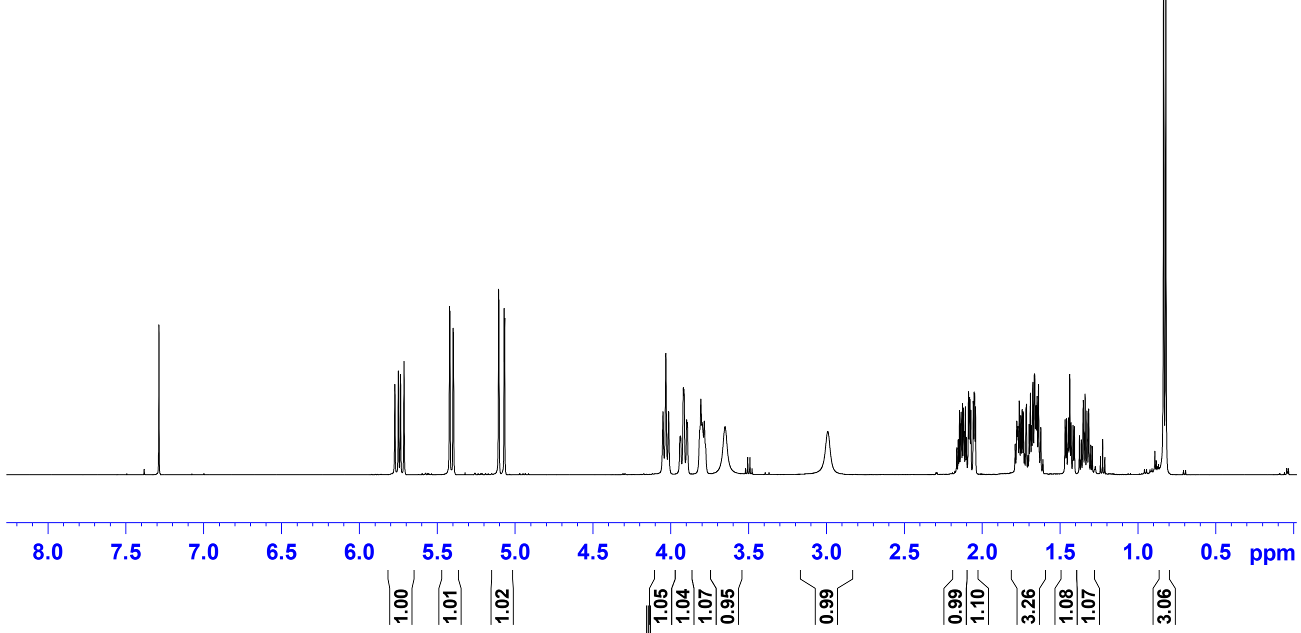

^{13}C NMR (125 MHz, $CDCl_3$)

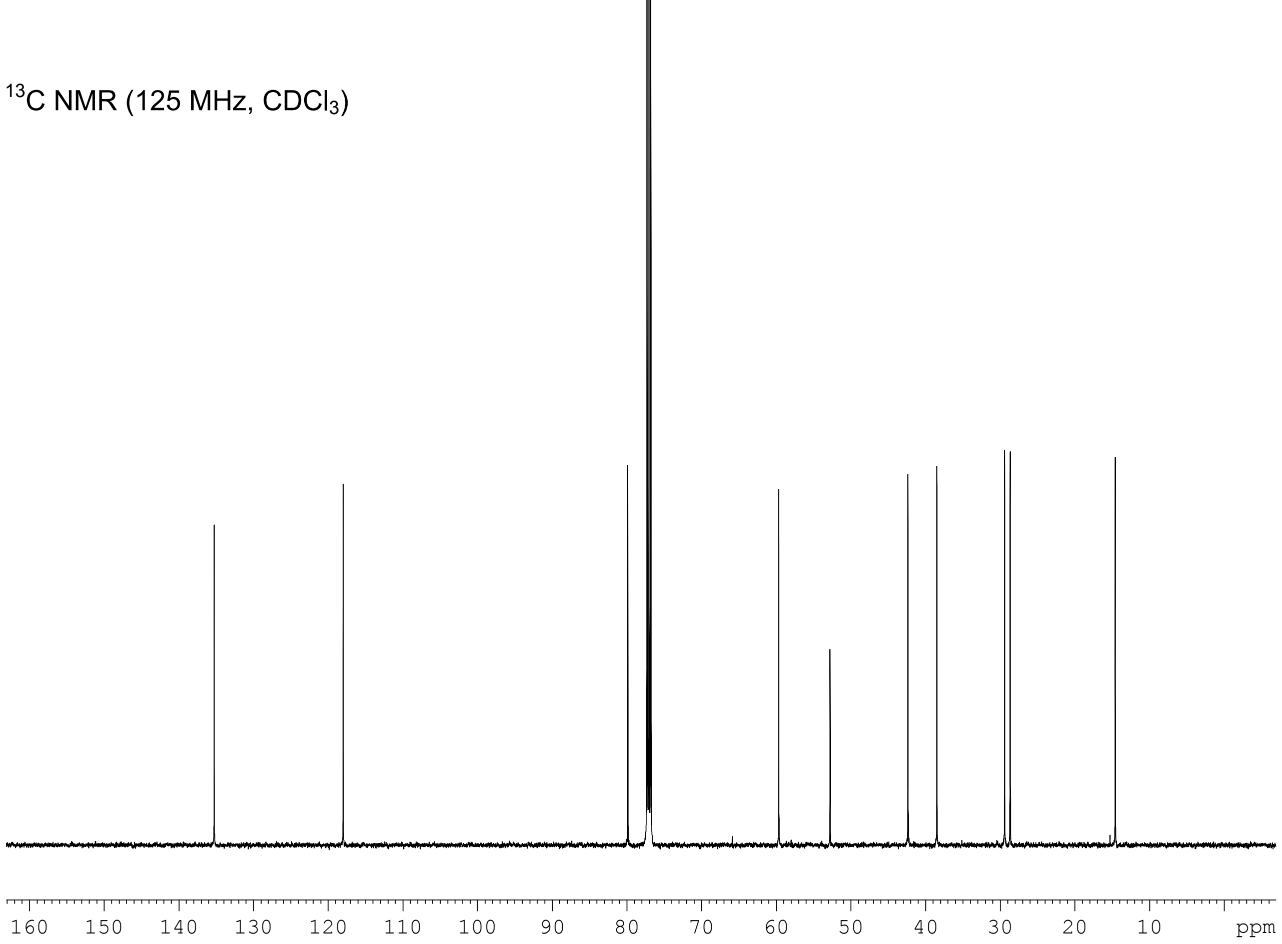

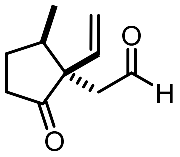

4

^{1}H NMR (500 MHz, $CDCl_3$)

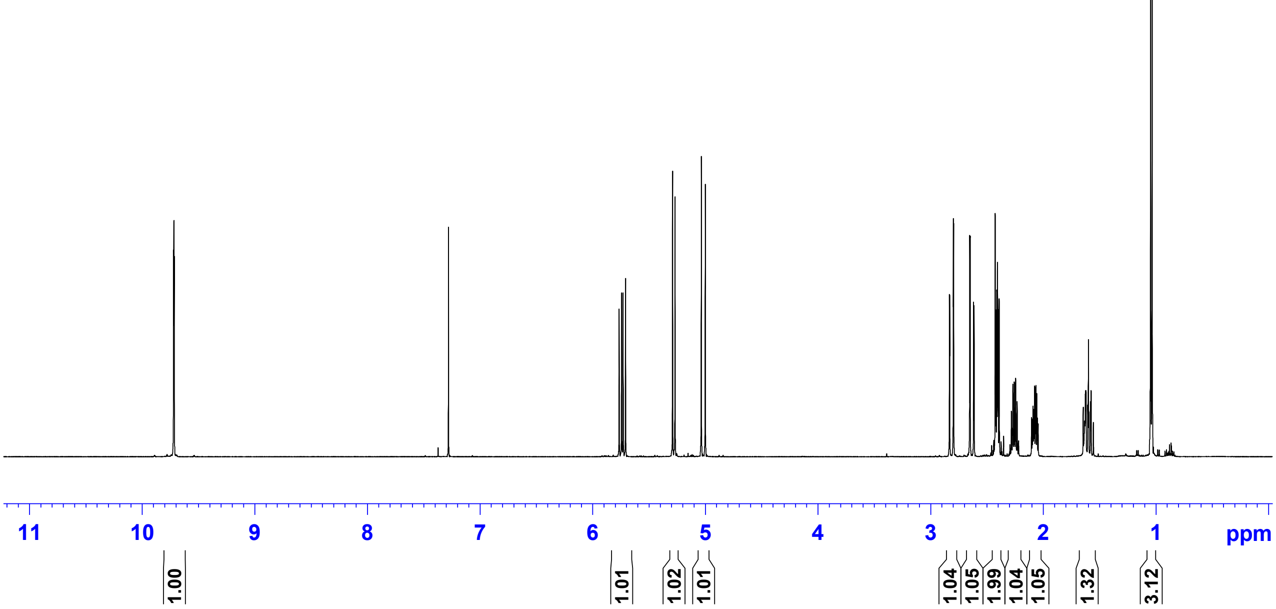

^{13}C NMR (125 MHz, $CDCl_3$)

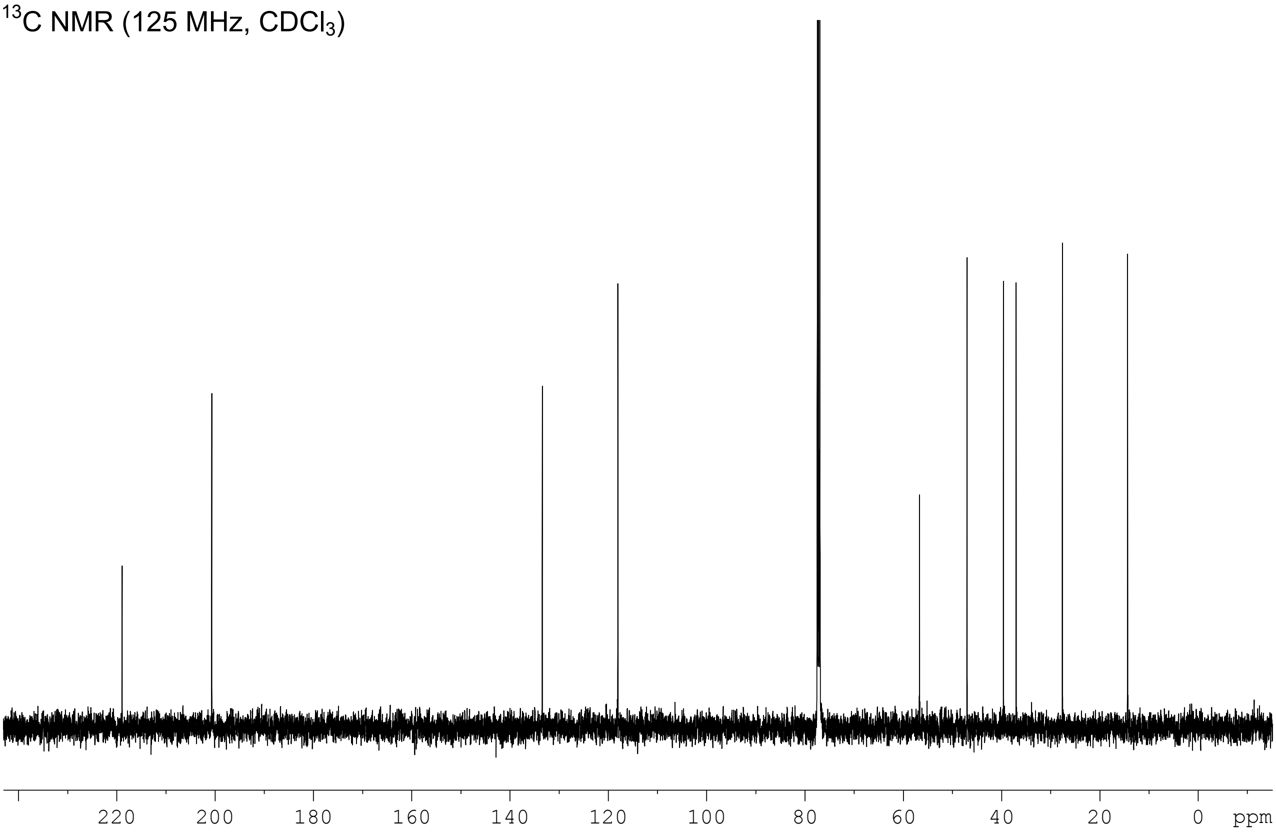

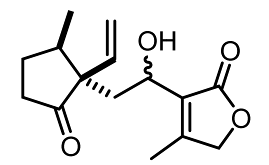

13

^{1}H NMR (500 MHz, $CDCl_3$)

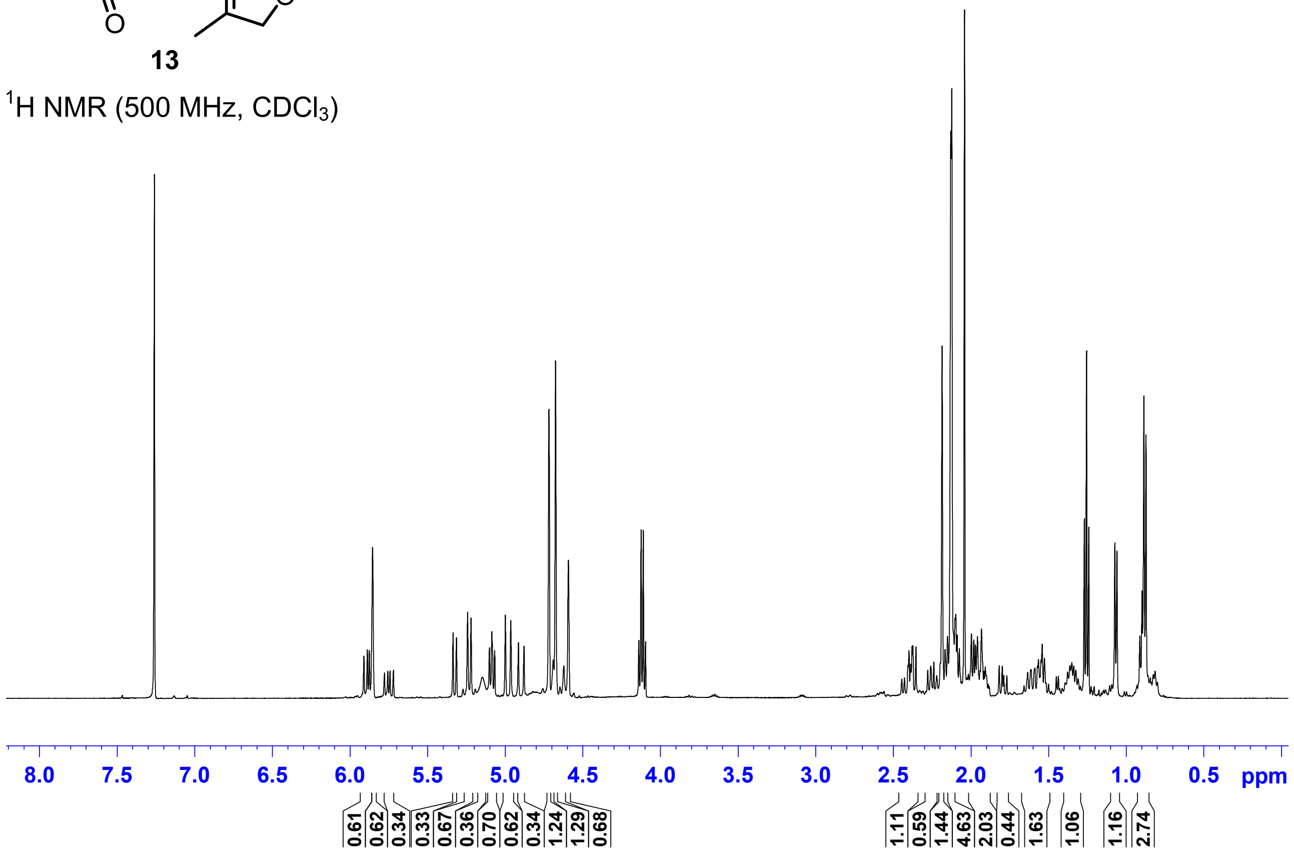

^{13}C NMR (125 MHz, $CDCl_3$)

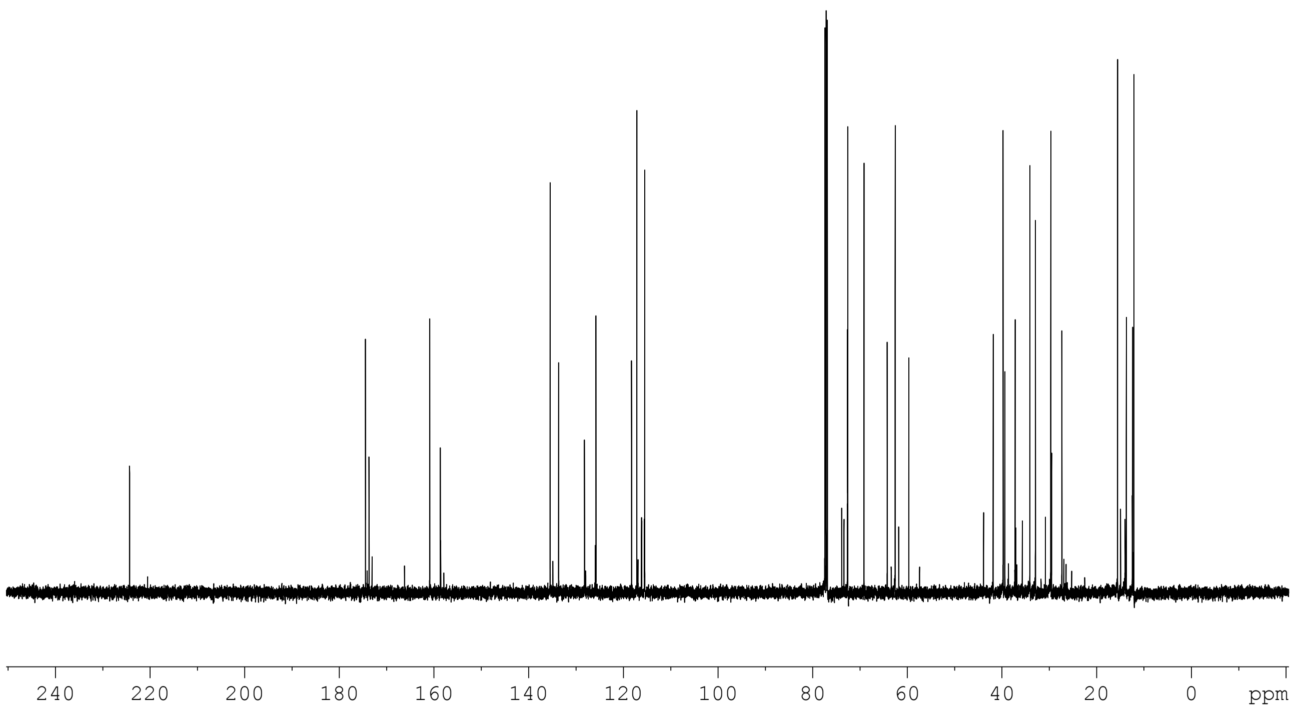

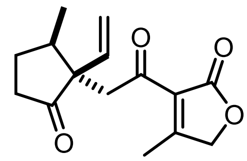

14

^{1}H NMR (500 MHz, $CDCl_3$)

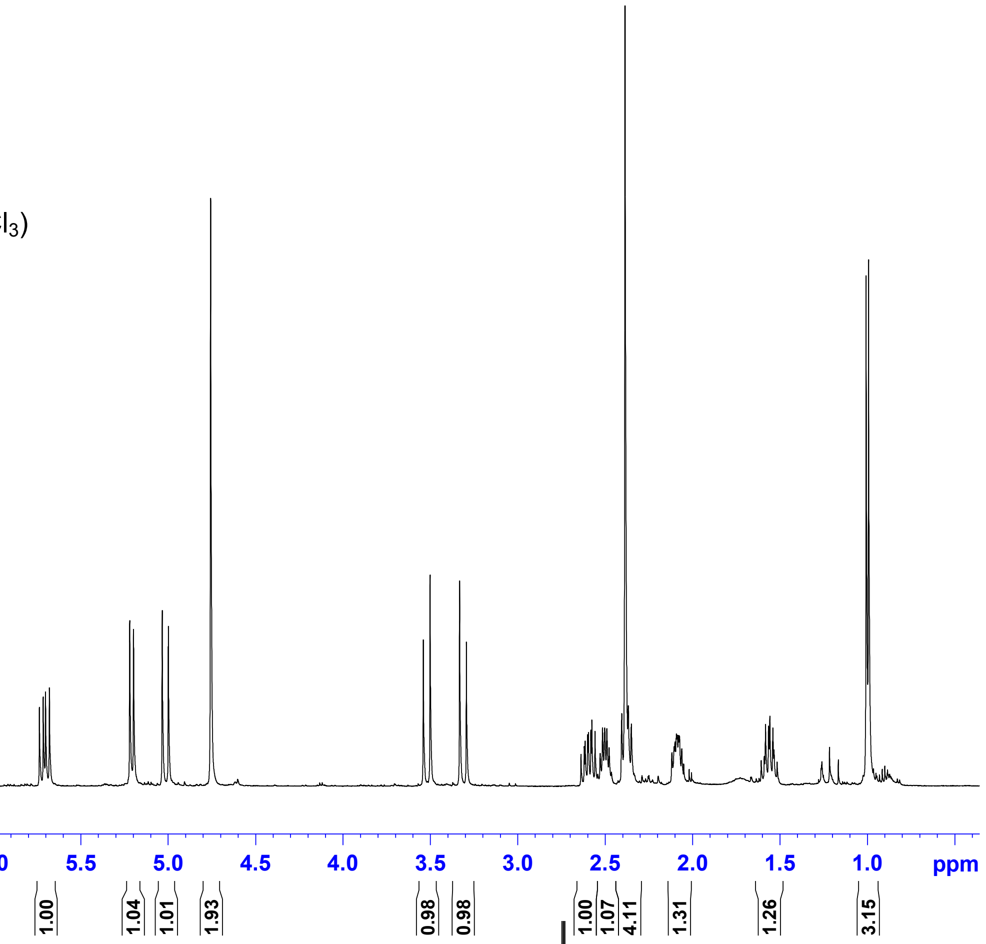

^{13}C NMR (125 MHz, $CDCl_3$)

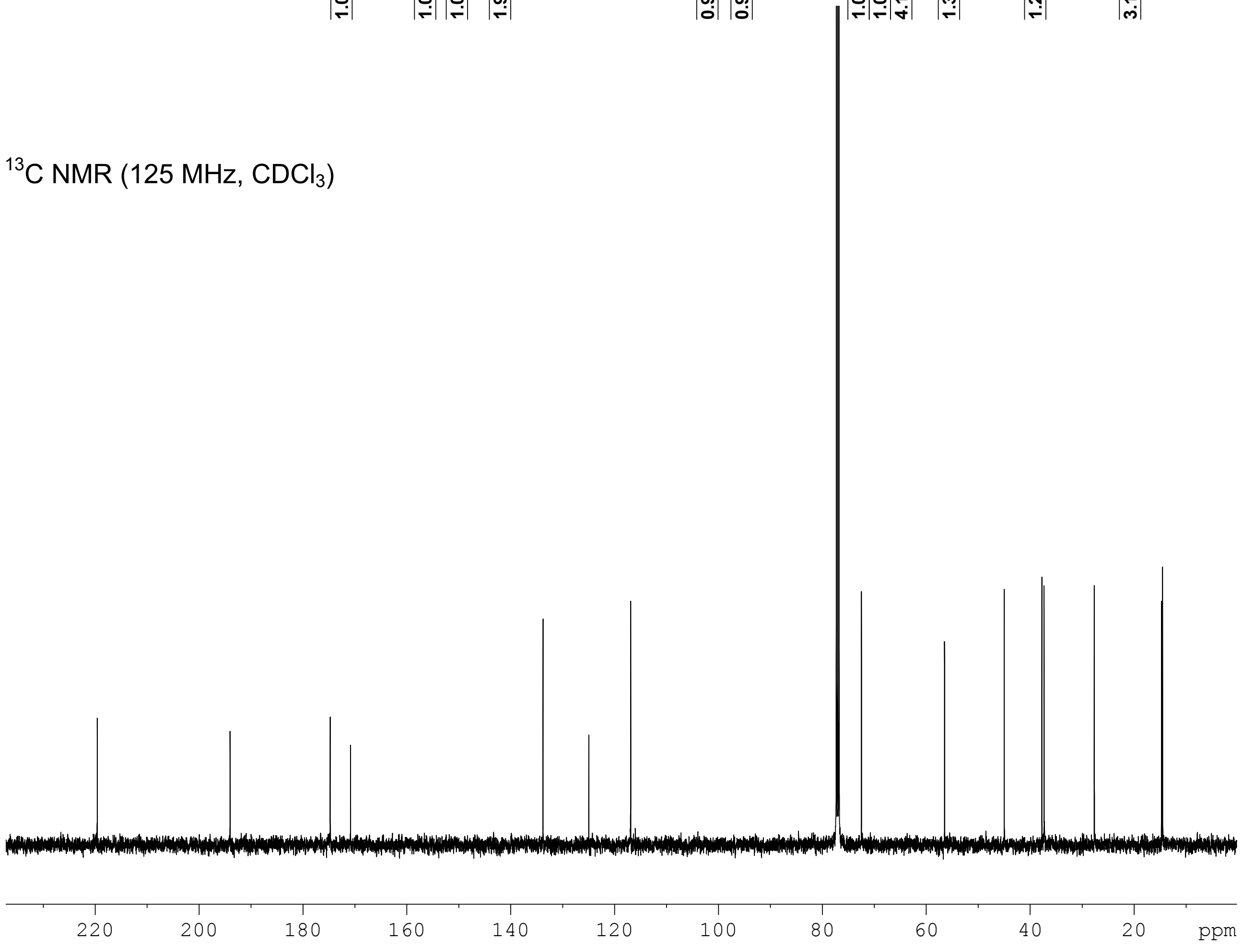

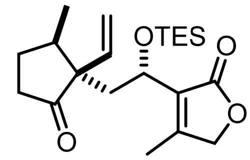

15

^{1}H NMR (500 MHz, $CDCl_3$)

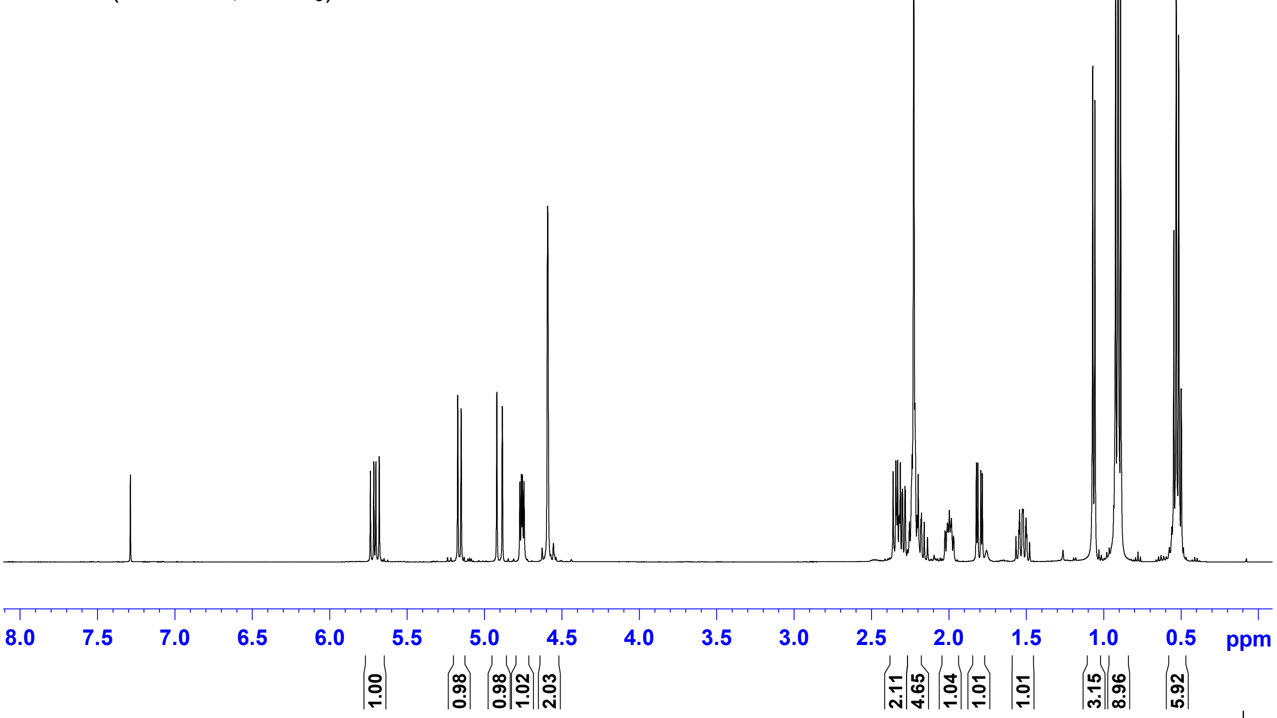

^{13}C NMR (125 MHz, $CDCl_3$)

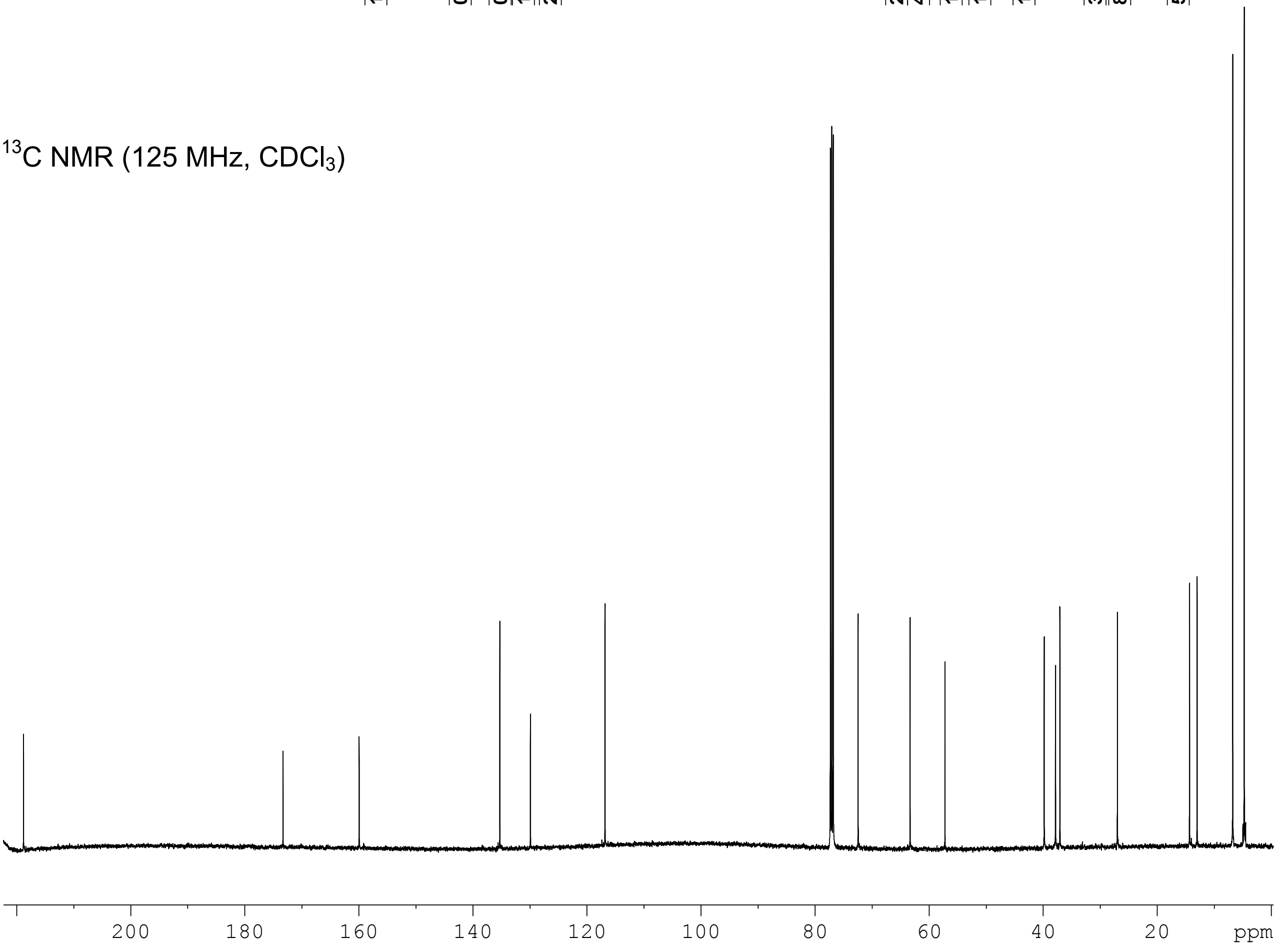

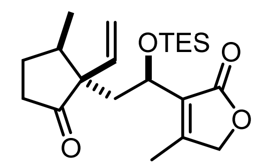

11-*epi*-**15**

^{1}H NMR (500 MHz, $CDCl_3$)

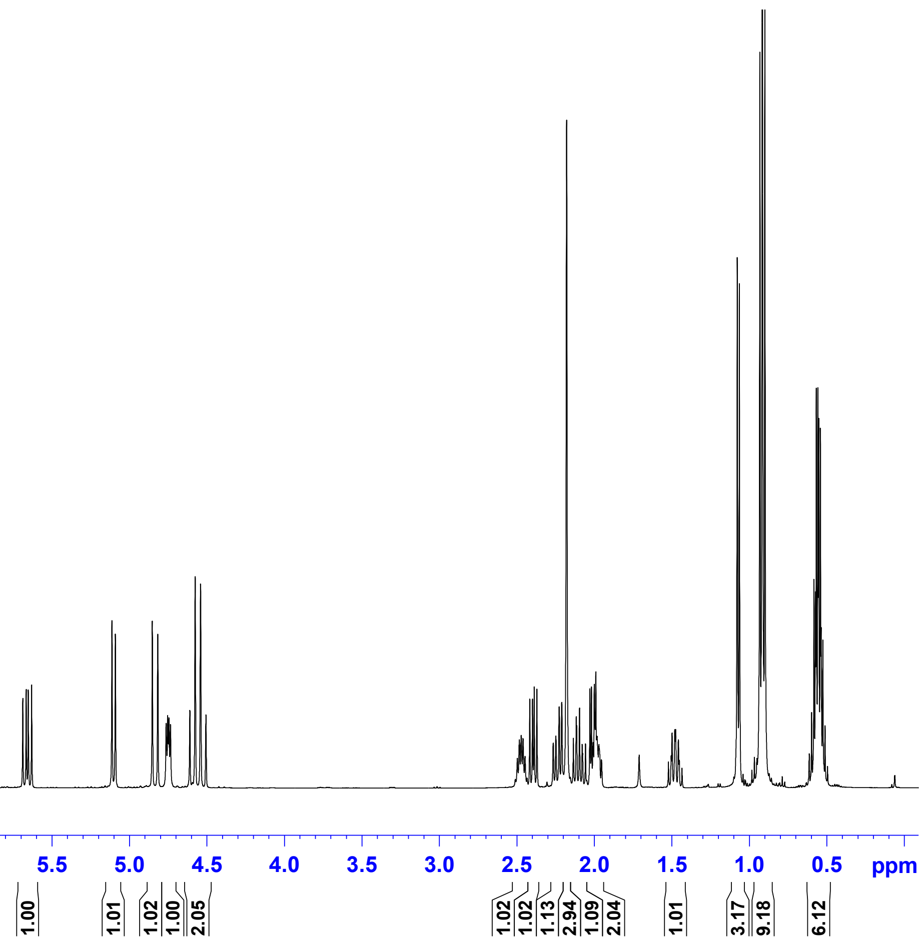

^{13}C NMR (125 MHz, $CDCl_3$)

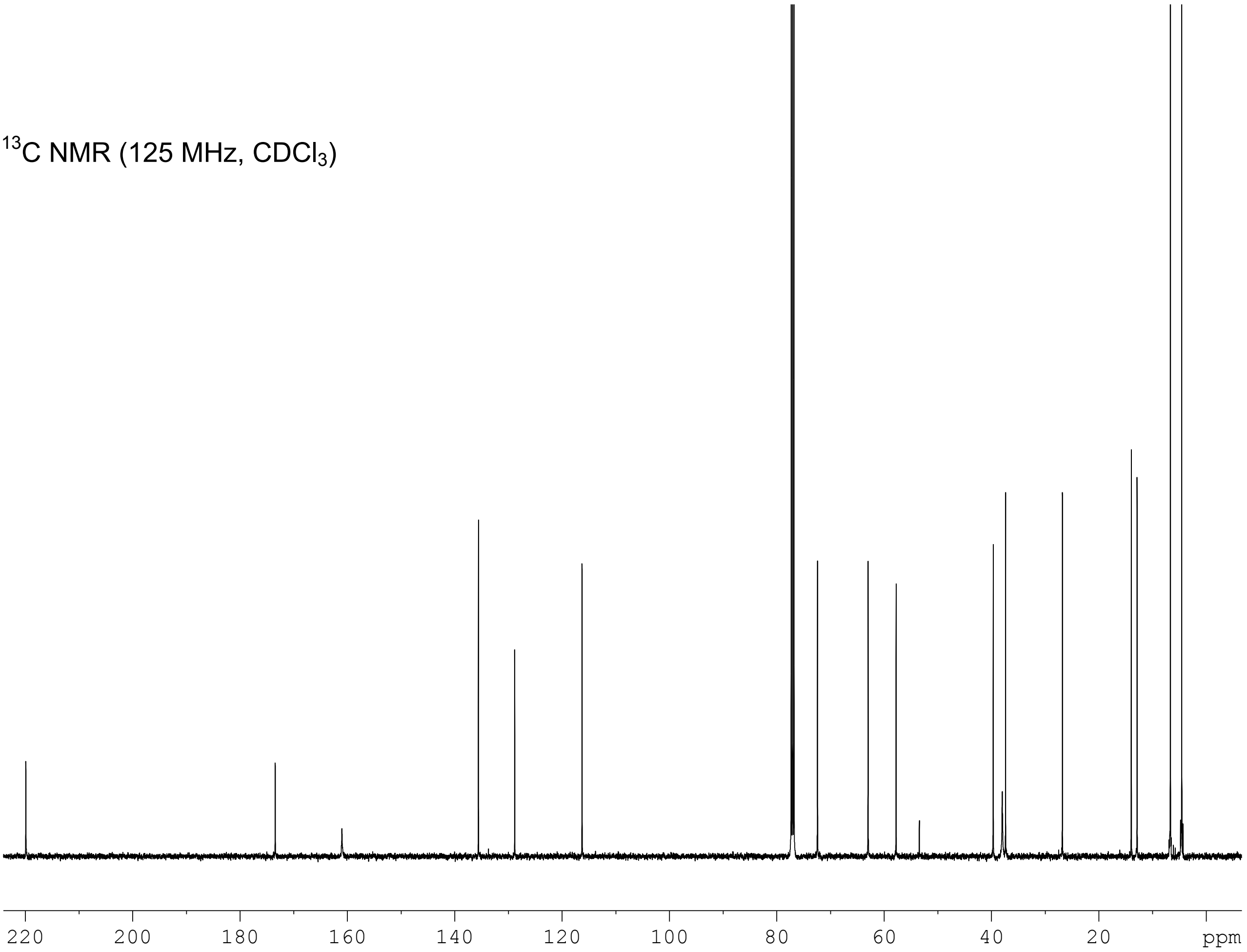

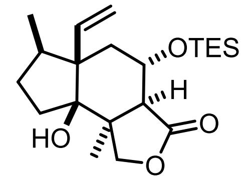

16

^{1}H NMR (500 MHz, $CDCl_3$)

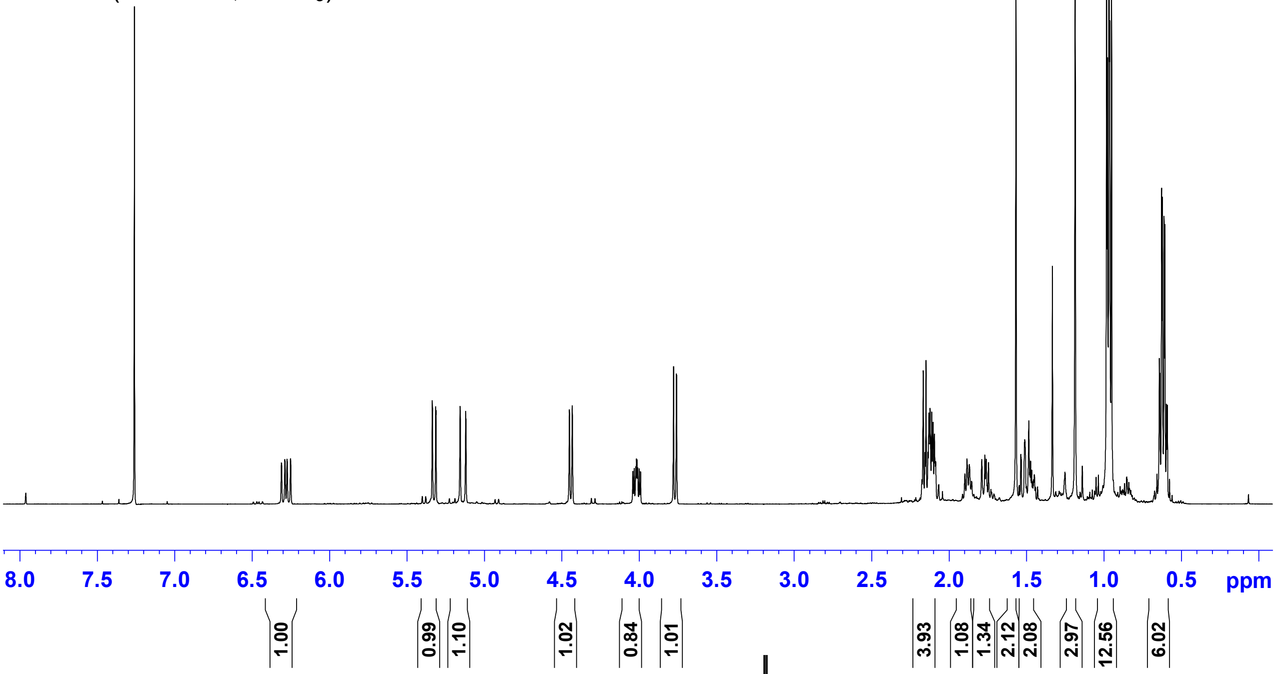

^{13}C NMR (125 MHz, $CDCl_3$)

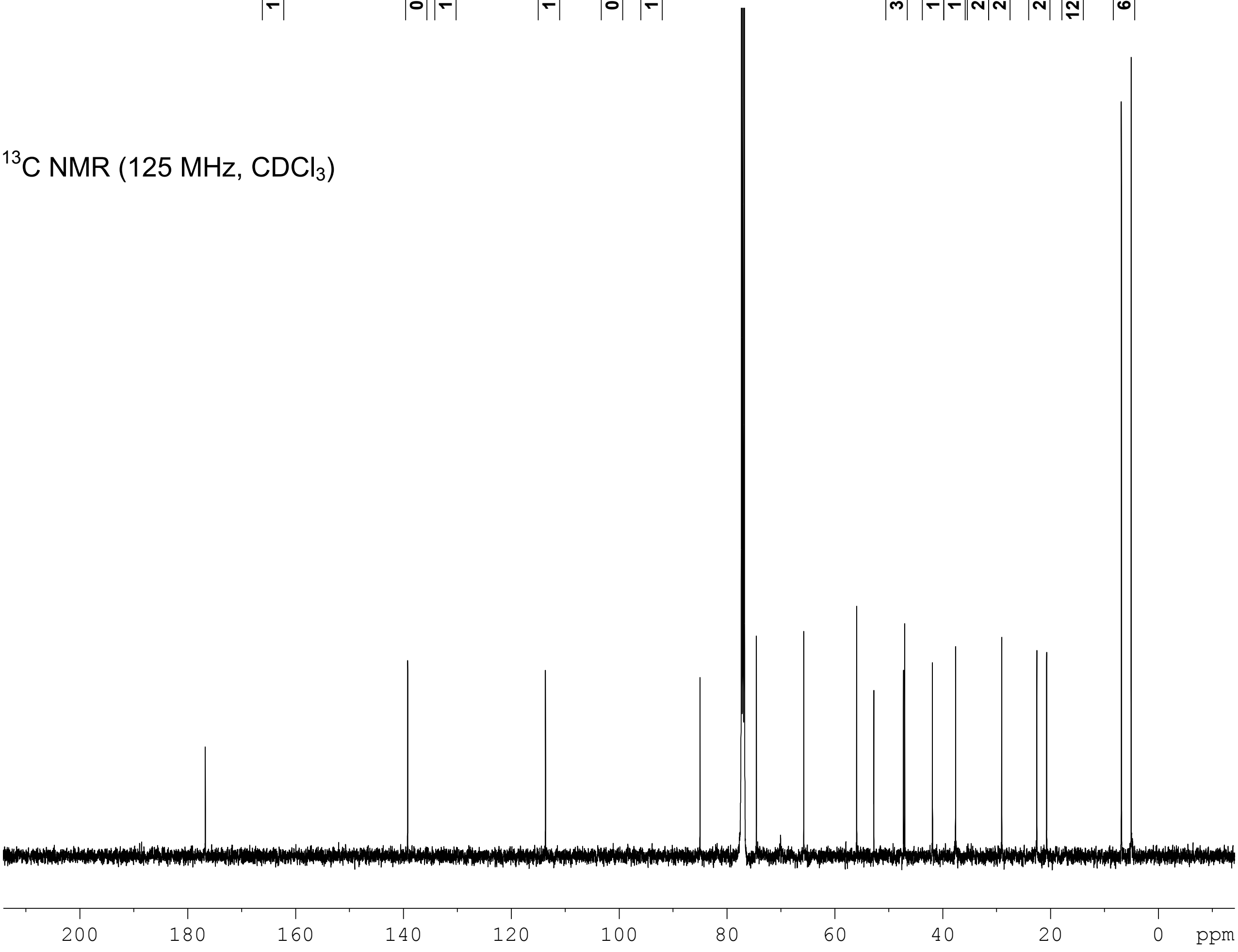

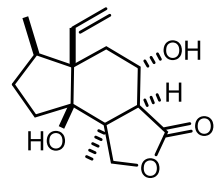

17

^{1}H NMR (500 MHz, $CDCl_3$)

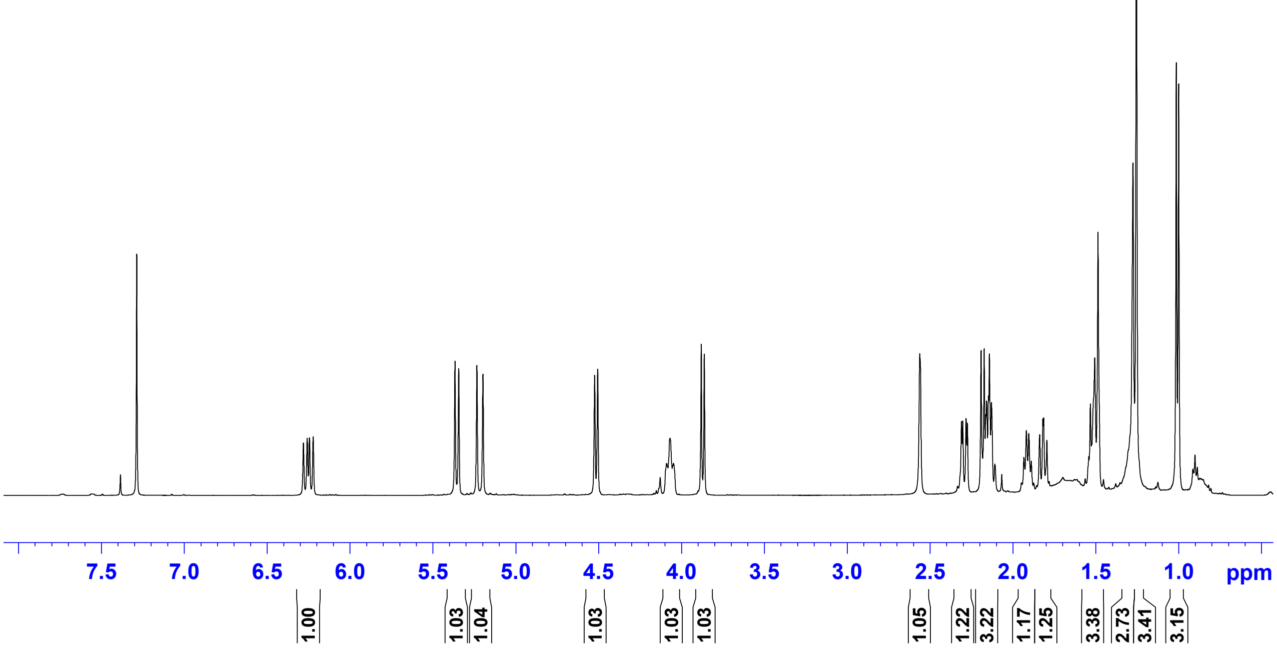

^{13}C NMR (125 MHz, $CDCl_3$)

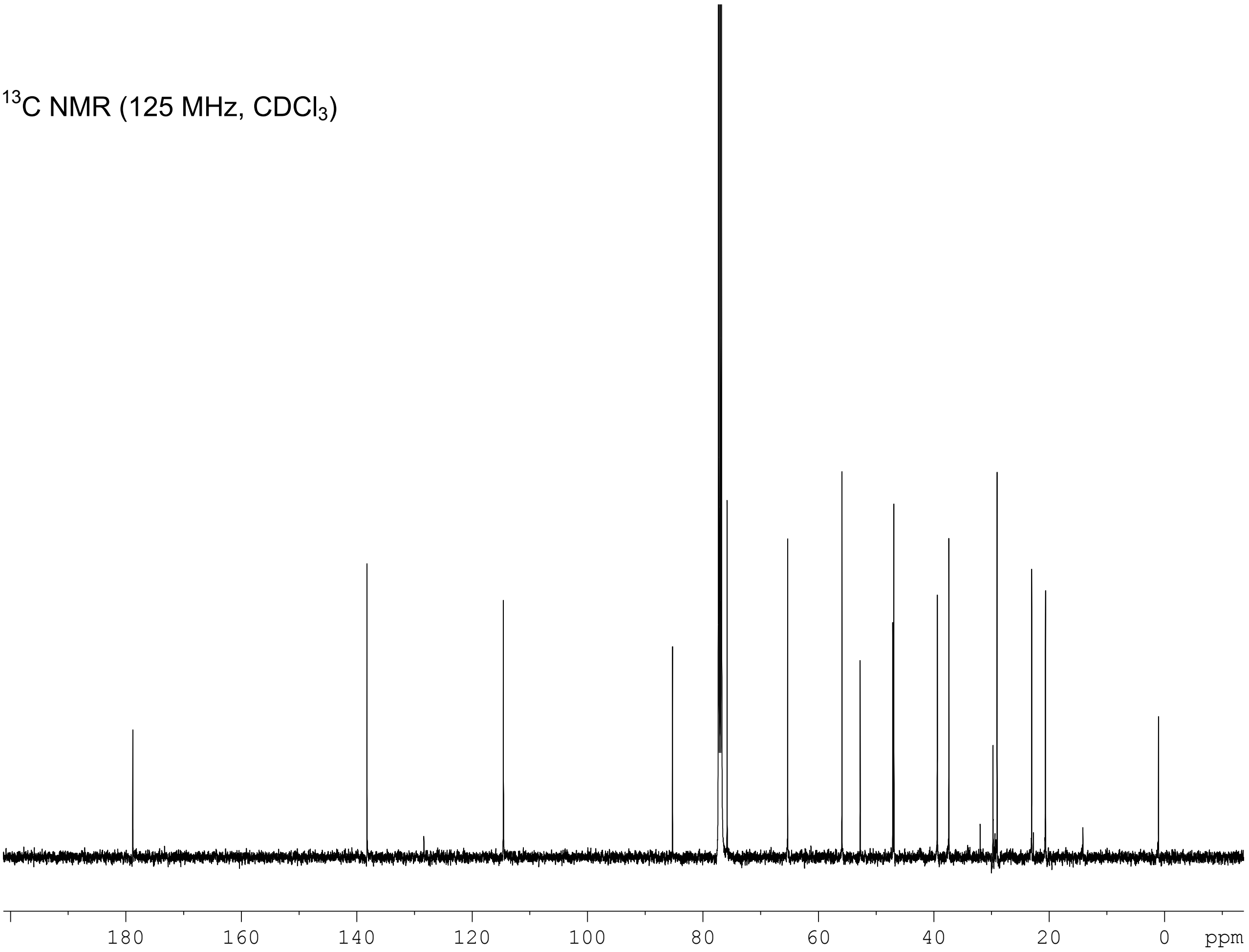

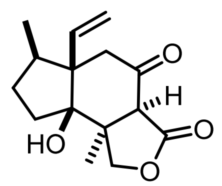

2

^{1}H NMR (500 MHz, $CDCl_3$)

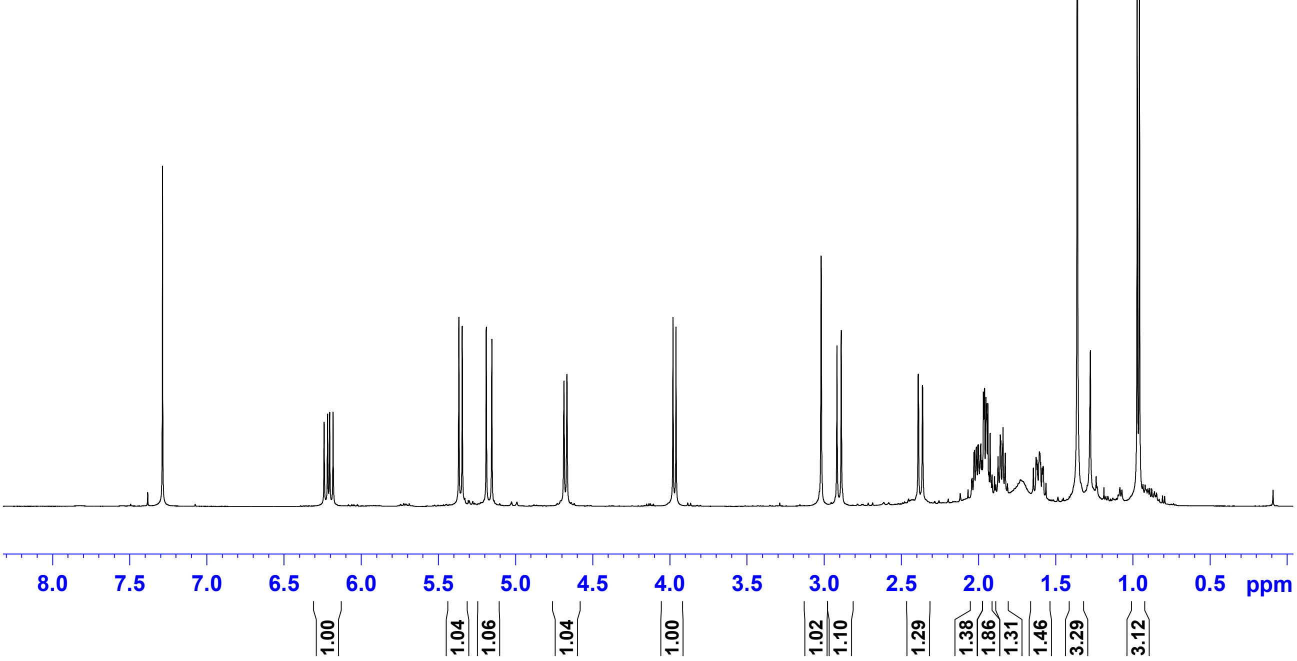

^{13}C NMR (125 MHz, $CDCl_3$)

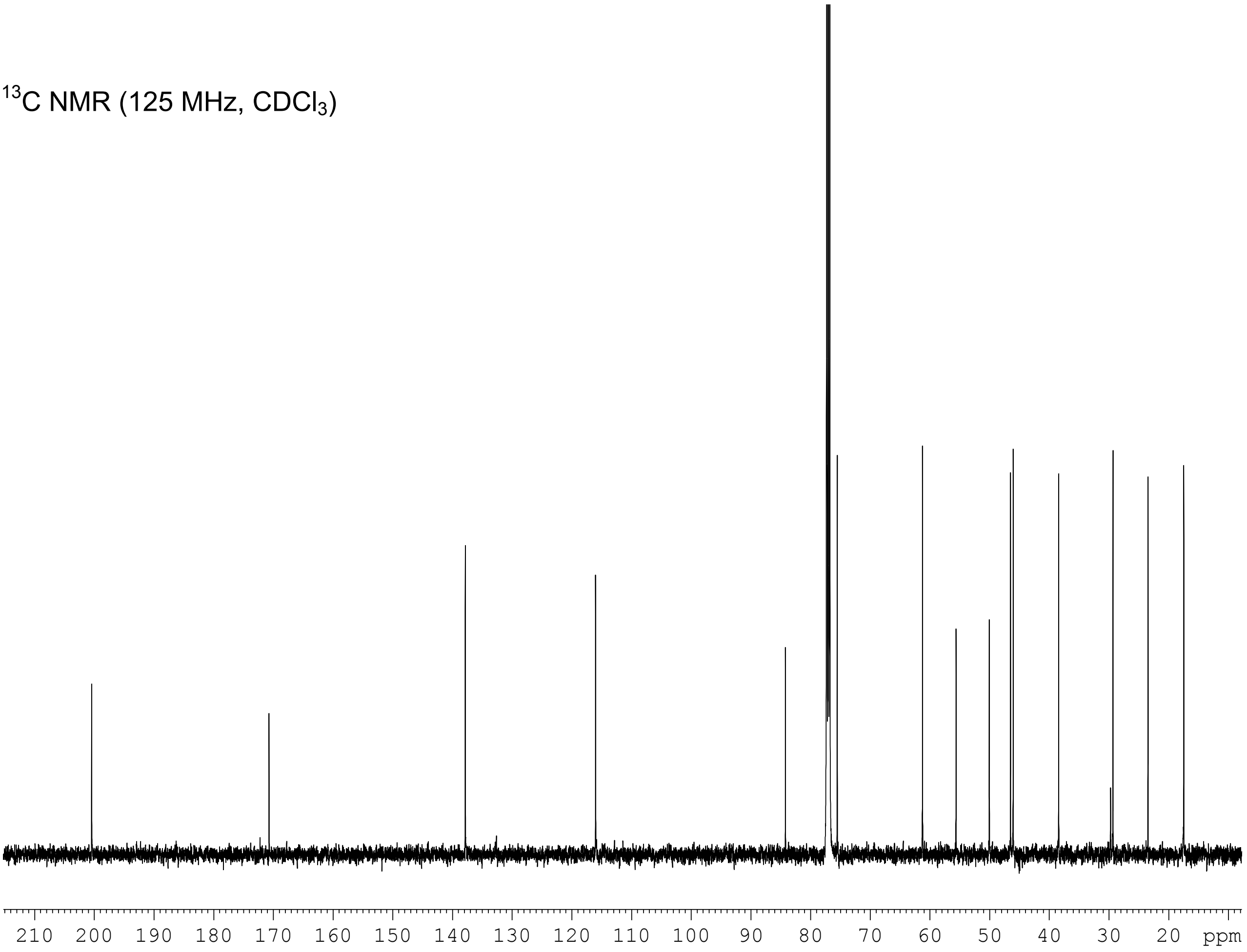

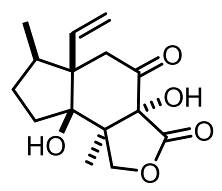

18

^{1}H NMR (500 MHz, $CDCl_3$)

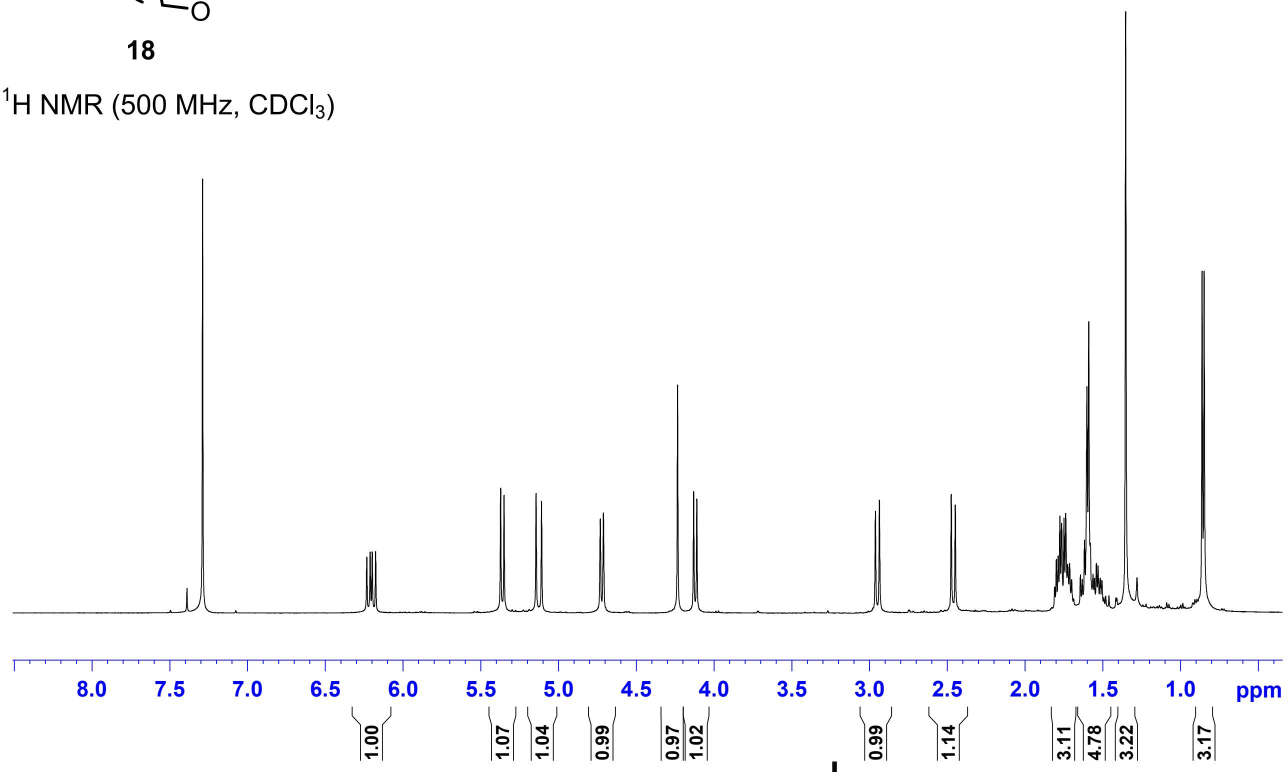

^{13}C NMR (125 MHz, $CDCl_3$)

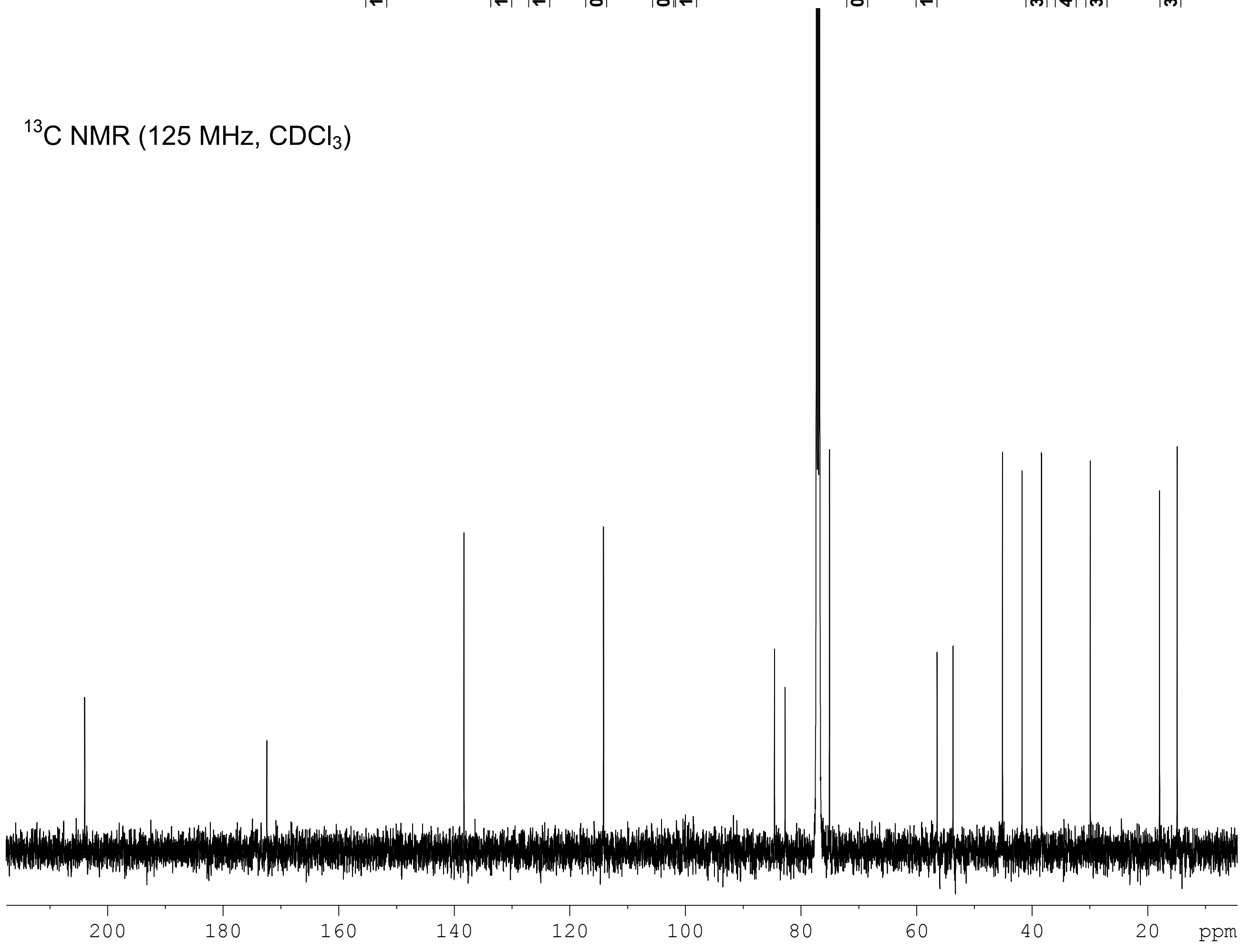

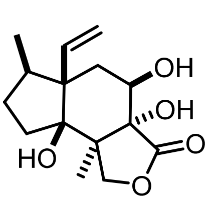

18a

^{1}H NMR (500 MHz, $CDCl_3$)

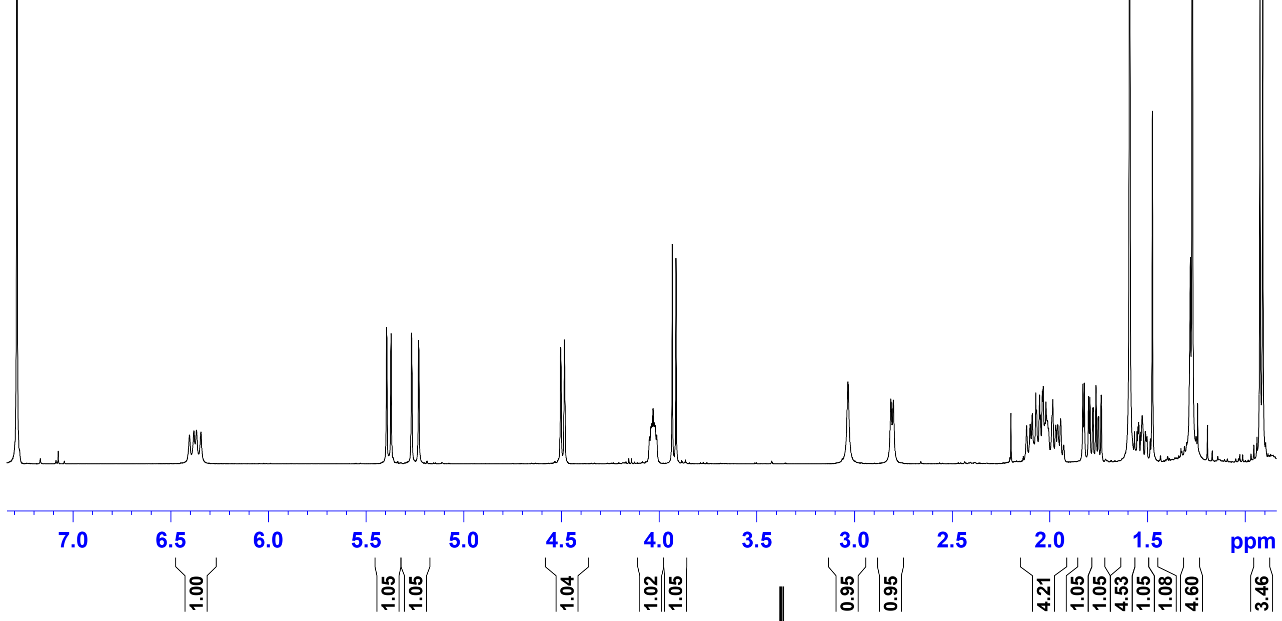

^{13}C NMR (125 MHz, $CDCl_3$)

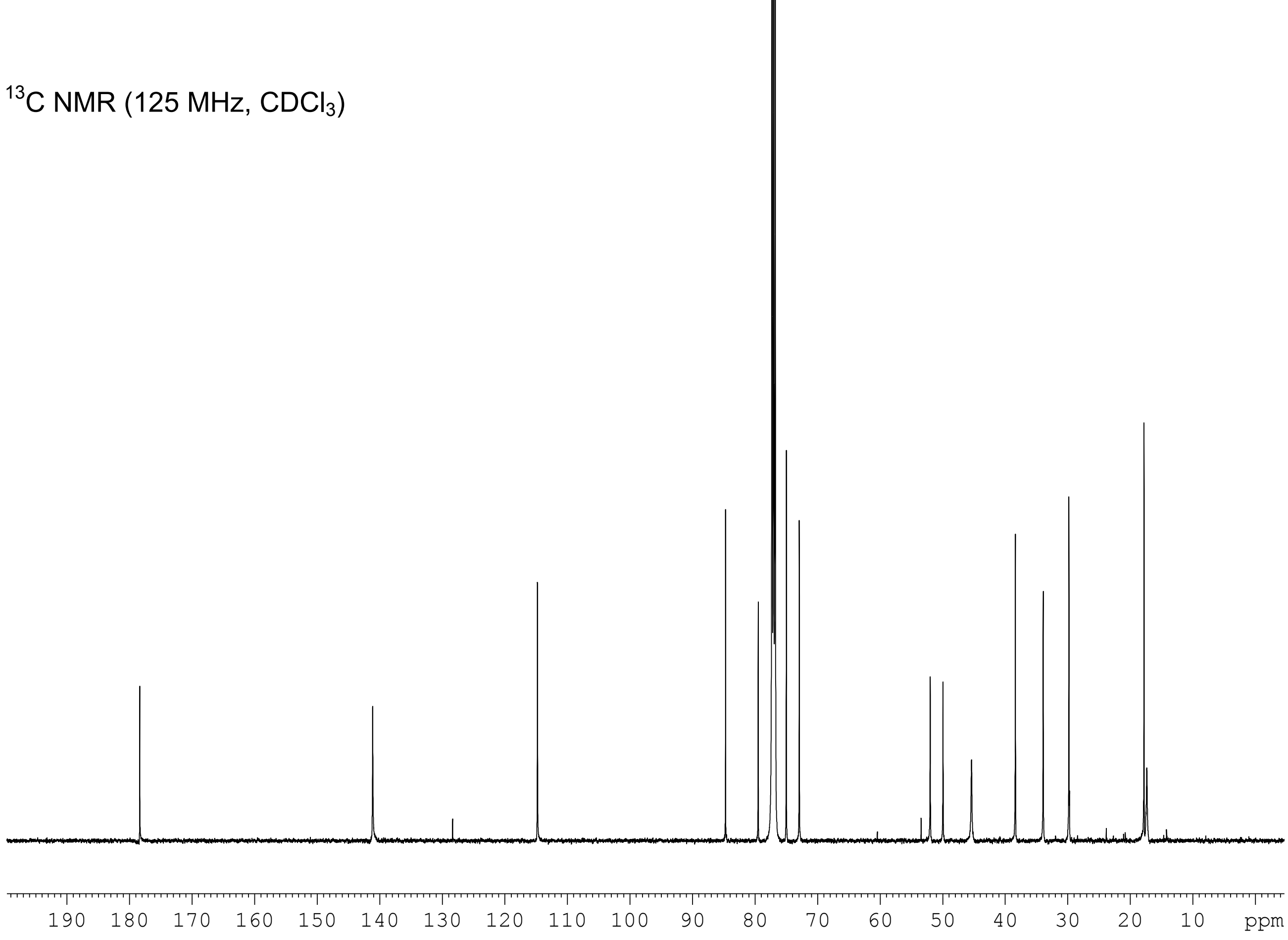

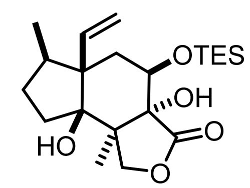

20

^{1}H NMR (500 MHz, $CDCl_3$)

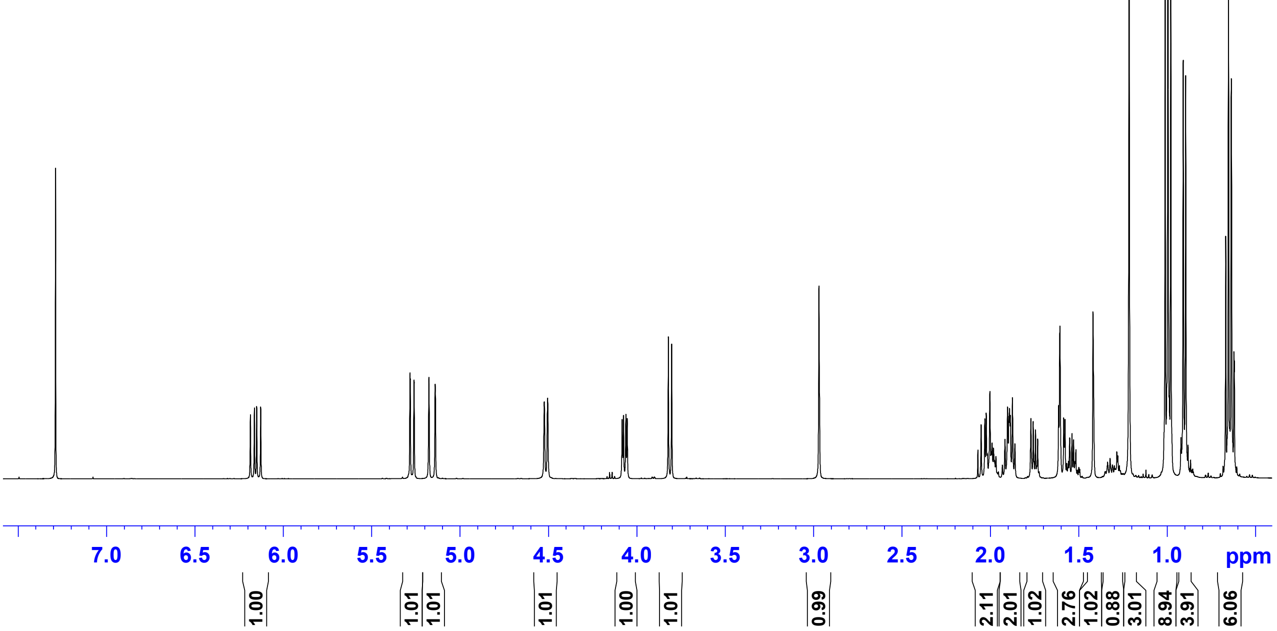

^{13}C NMR (125 MHz, $CDCl_3$)

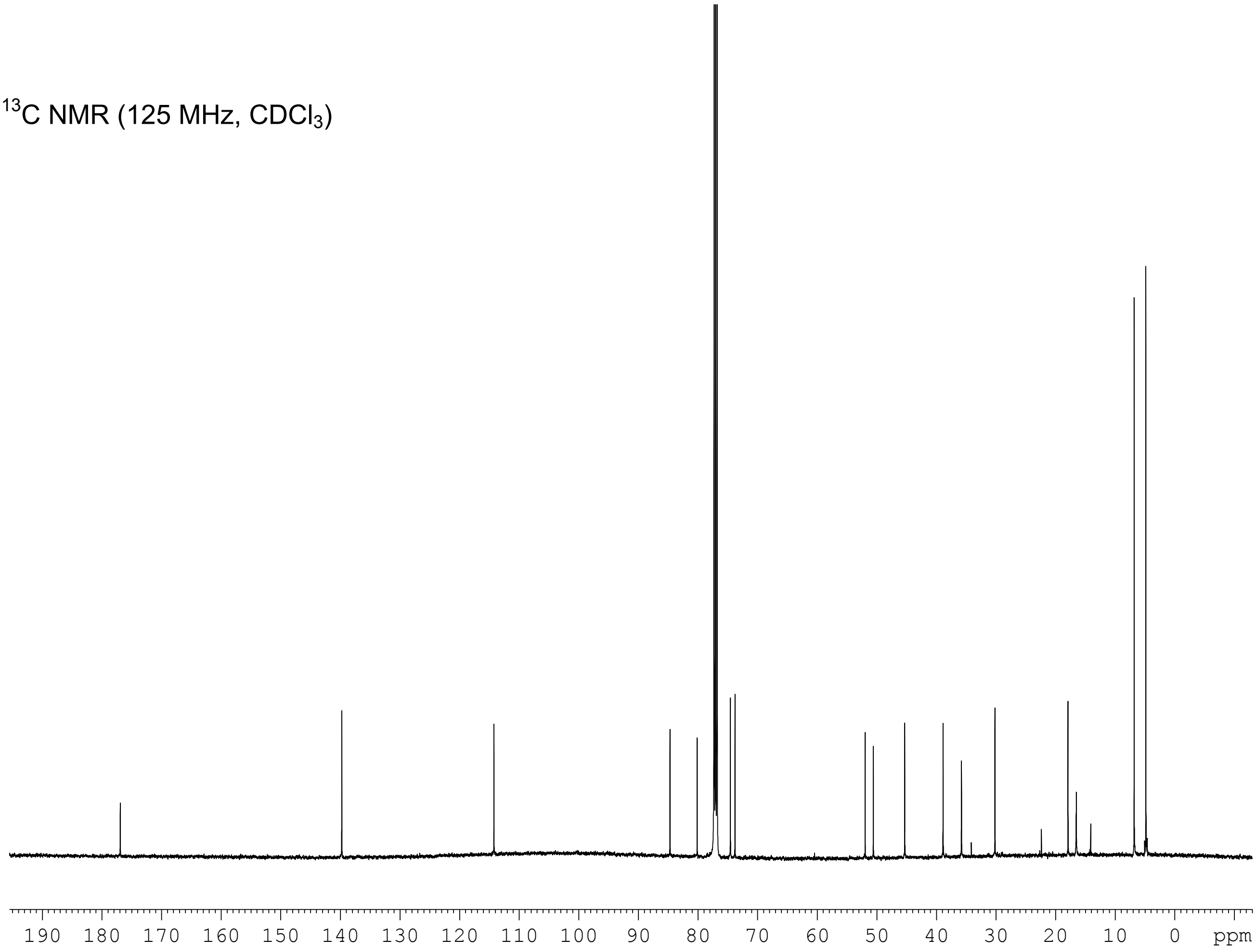

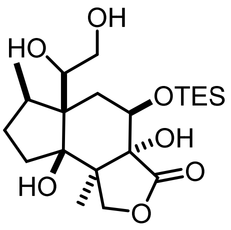

20a

^{1}H NMR (500 MHz, $CDCl_3$)

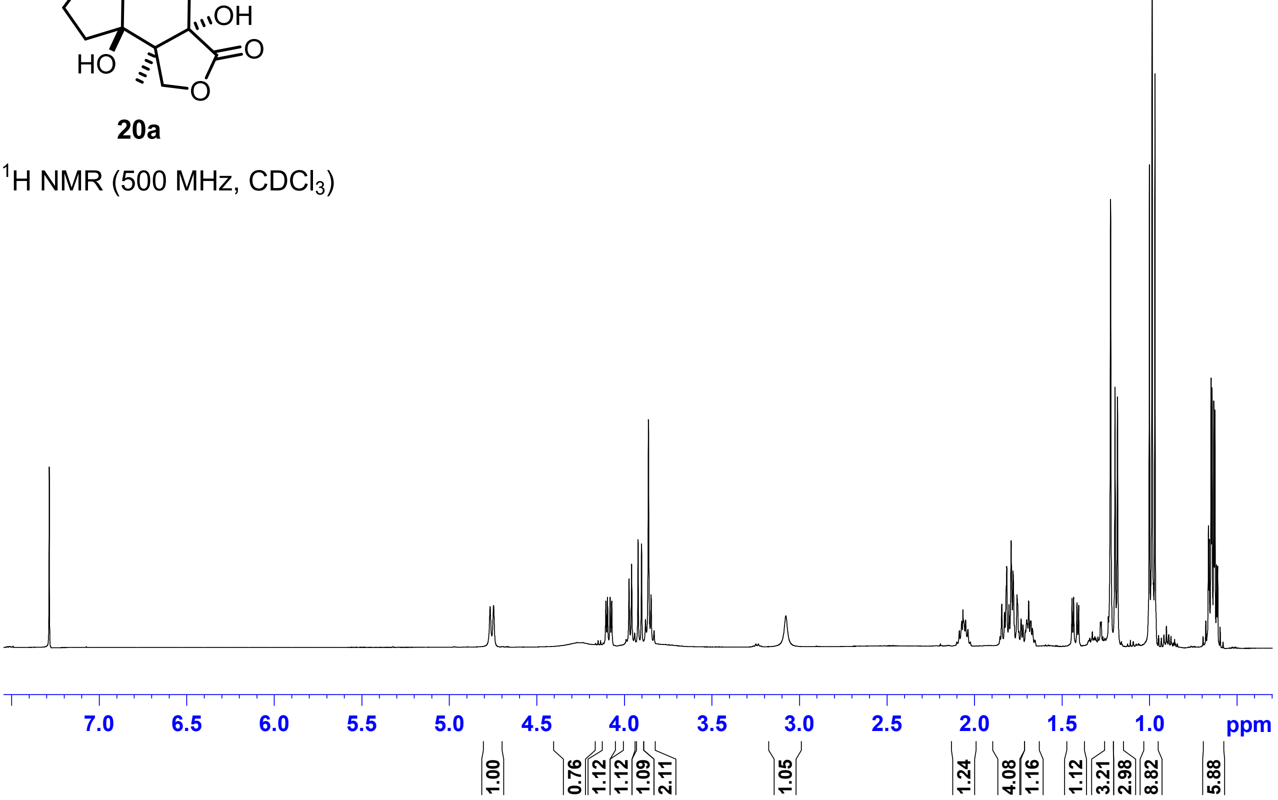

^{13}C NMR (125 MHz, $CDCl_3$)

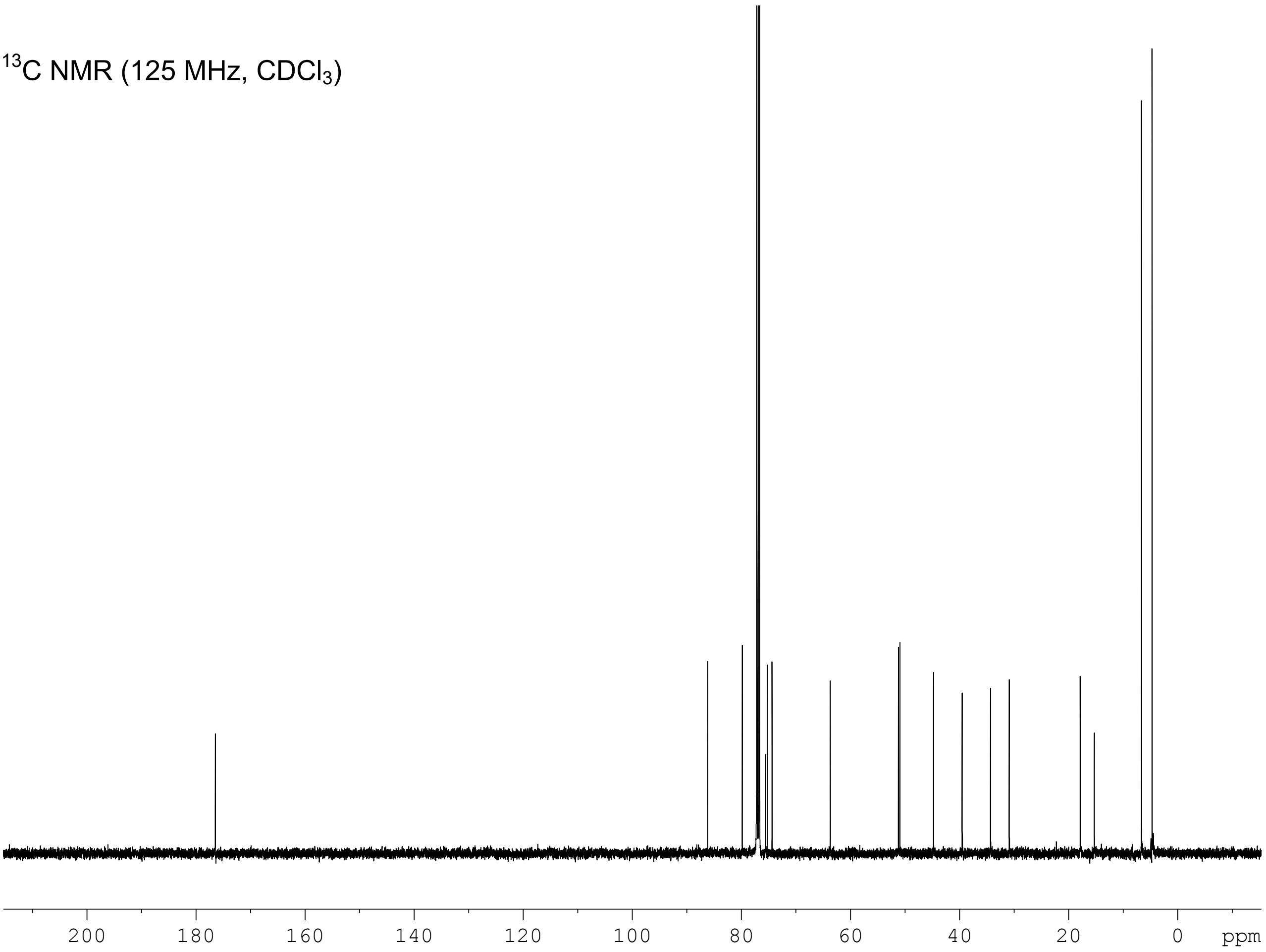

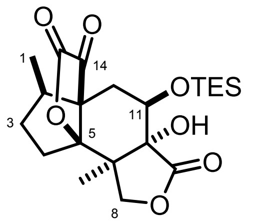

20b

^{1}H NMR (500 MHz, $CDCl_3$)

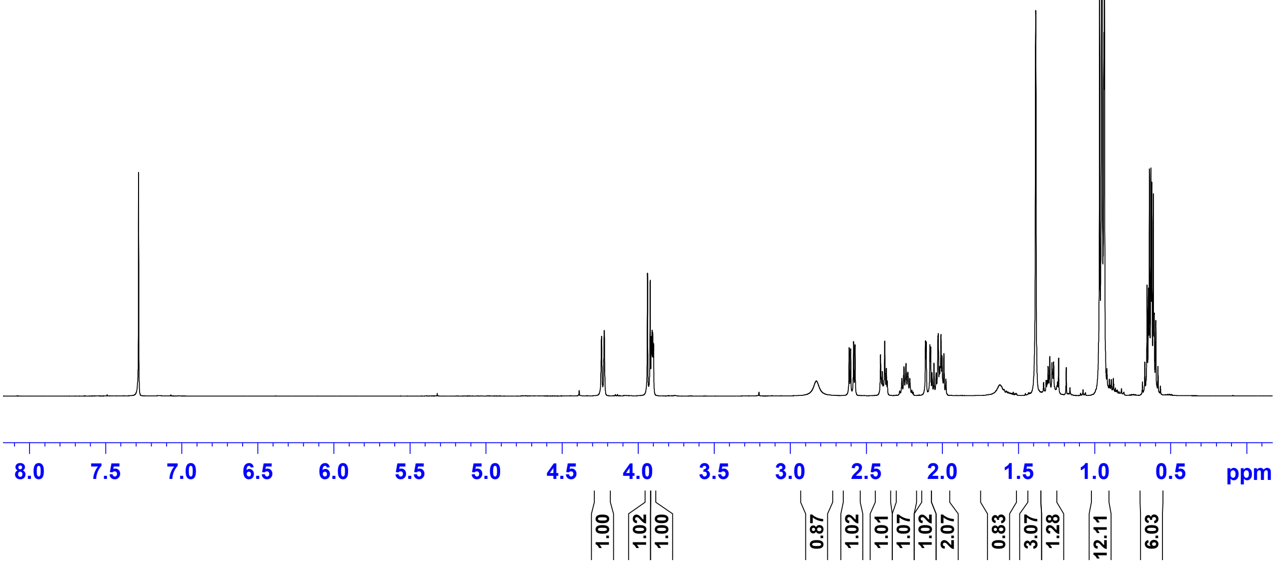

^{13}C NMR (125 MHz, $CDCl_3$)

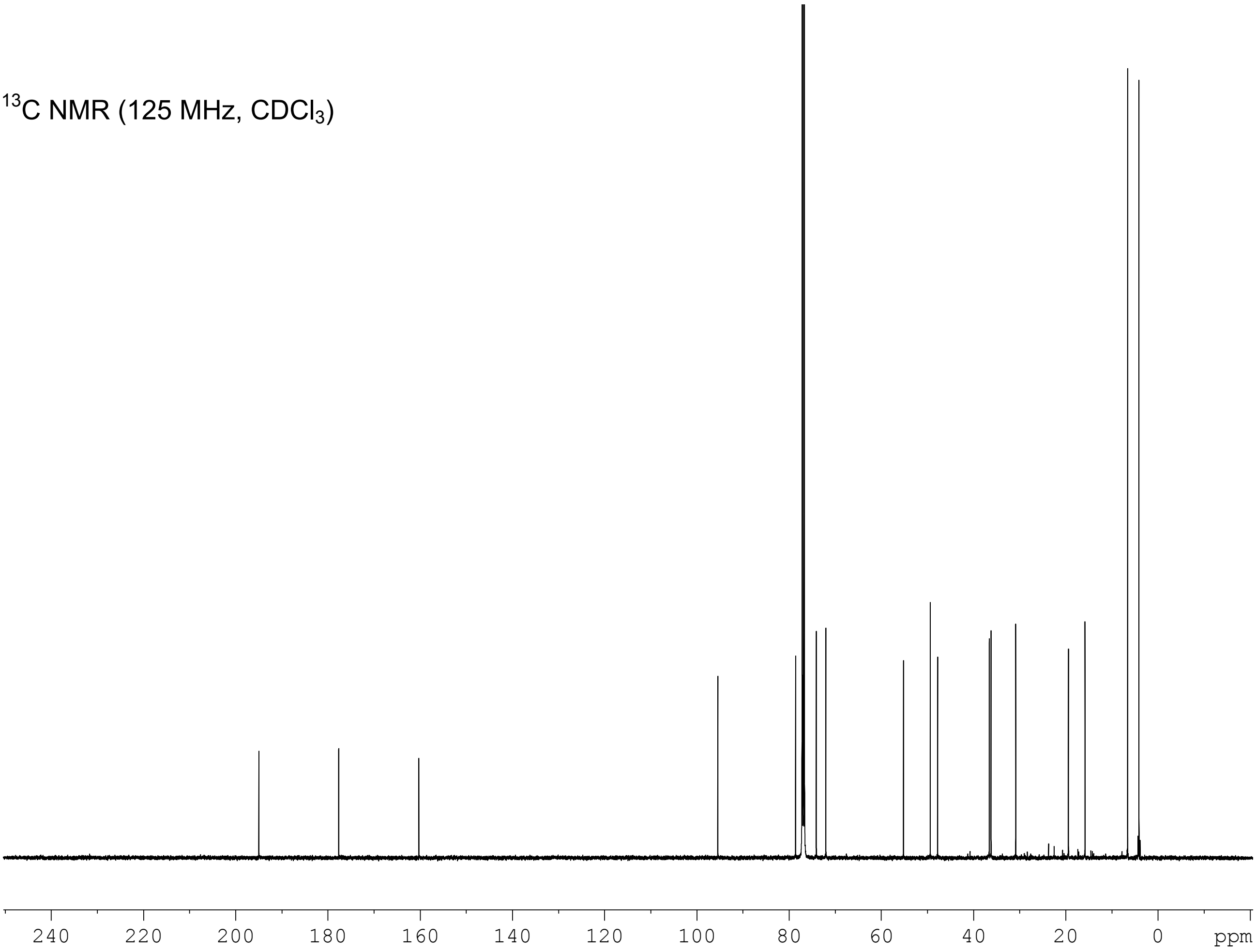

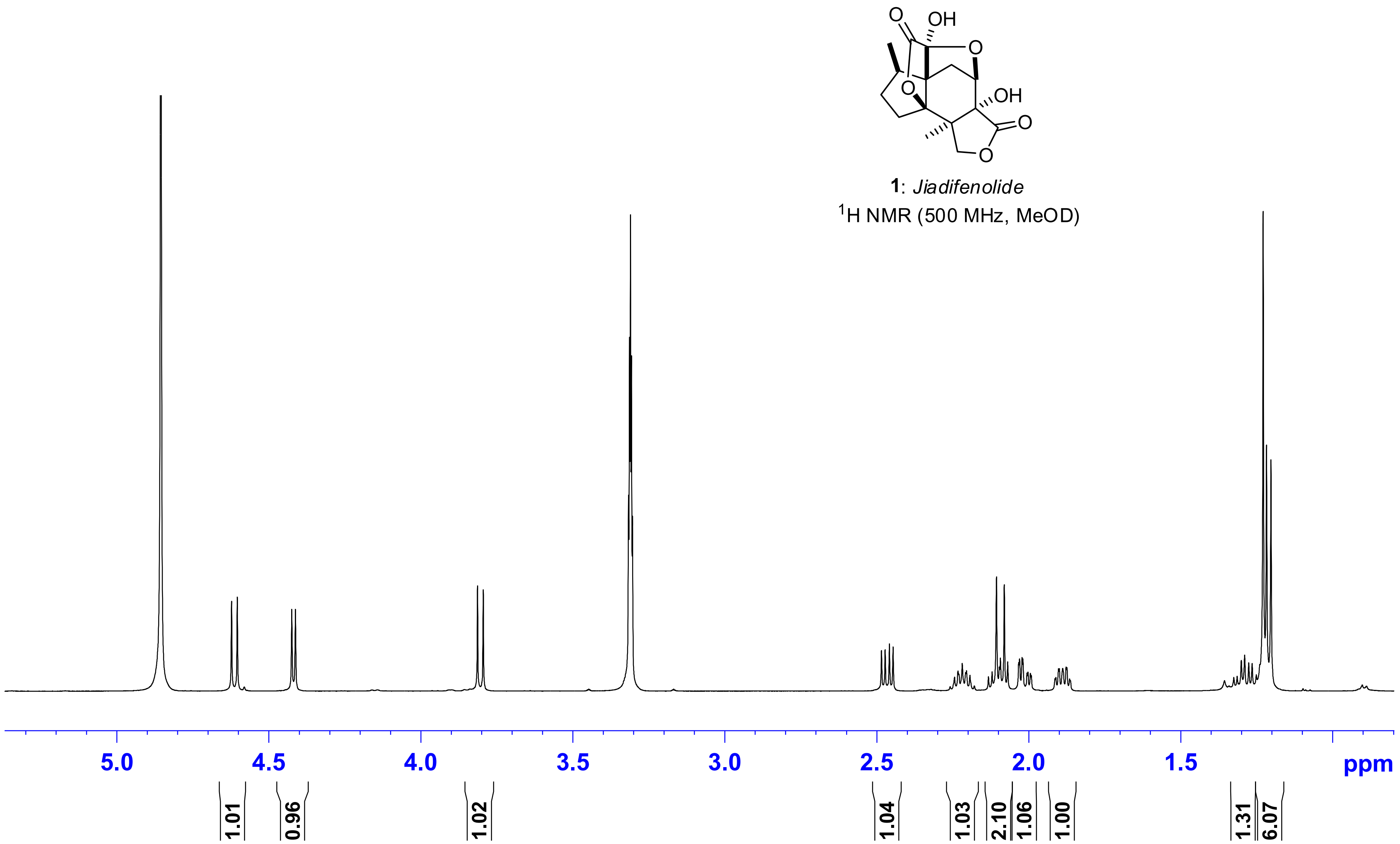

1: *Jiadifenolide*

^{1}H NMR (500 MHz, MeOD)

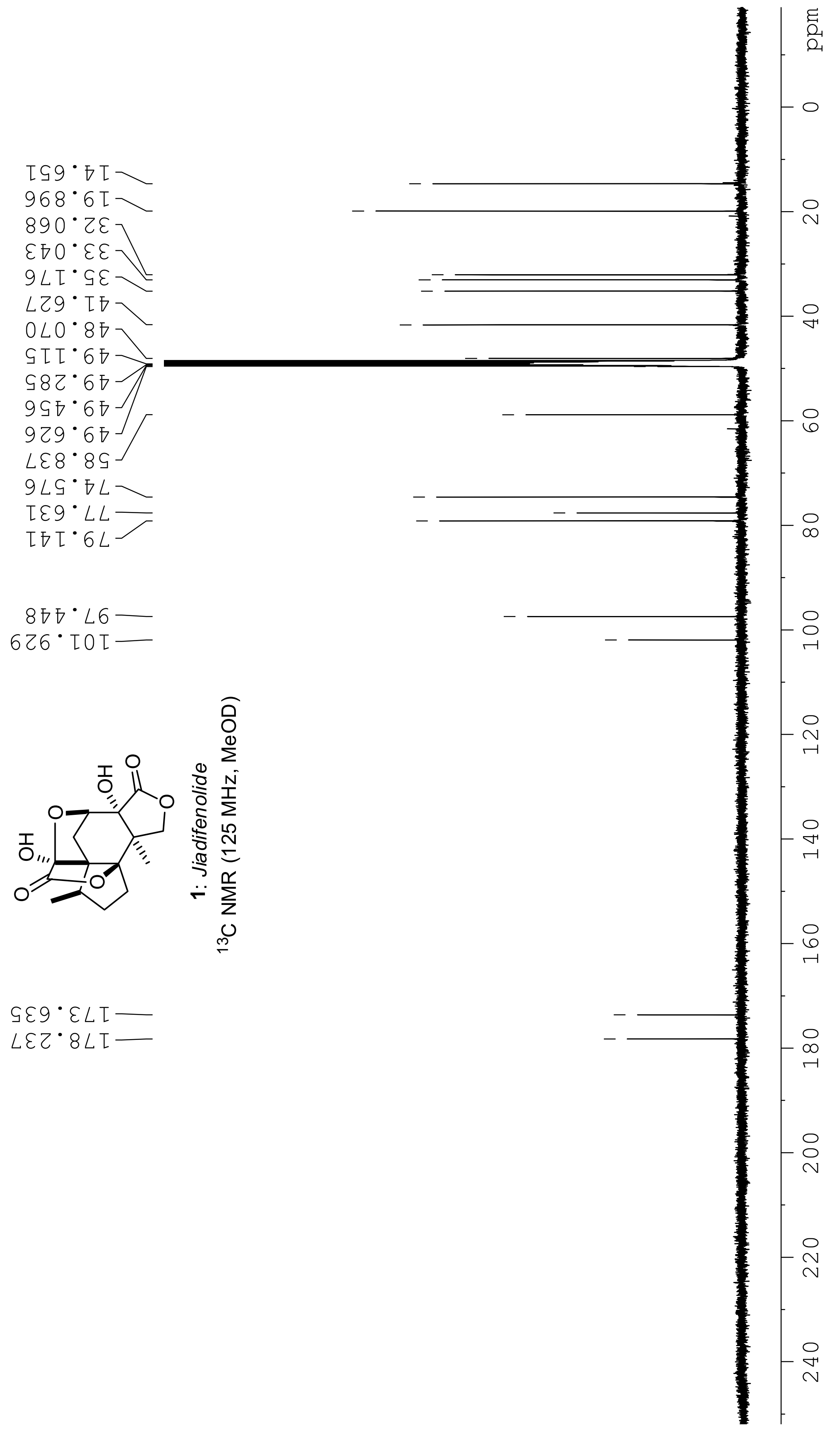

1: *Jiadifenolide*
^{13}C NMR (125 MHz, MeOD)

COSY spectrum of jiadifenolide (500 MHz, MeOD)

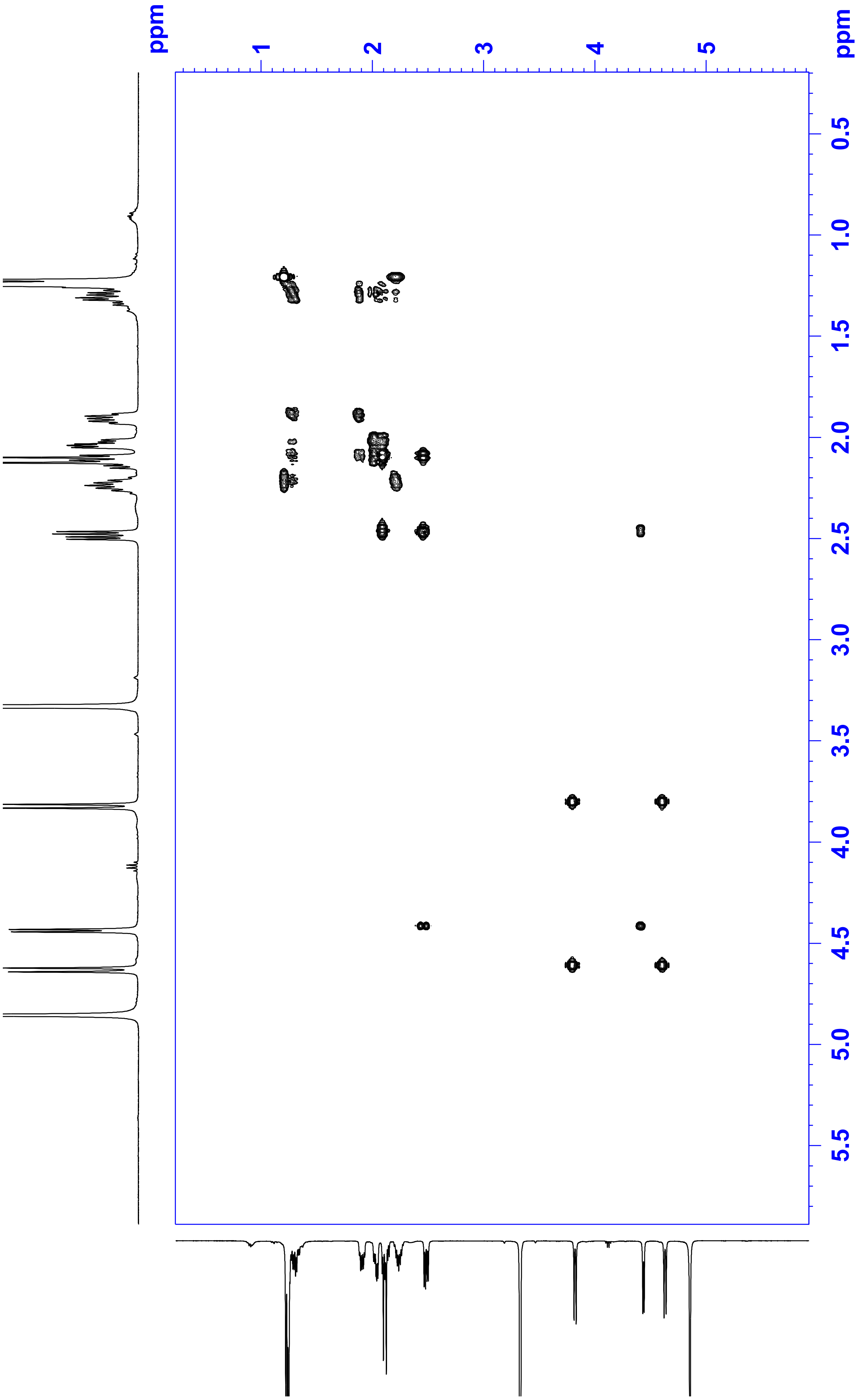

HMBC spectrum of synthetic jiadifenolide (500 MHz, MeOD)

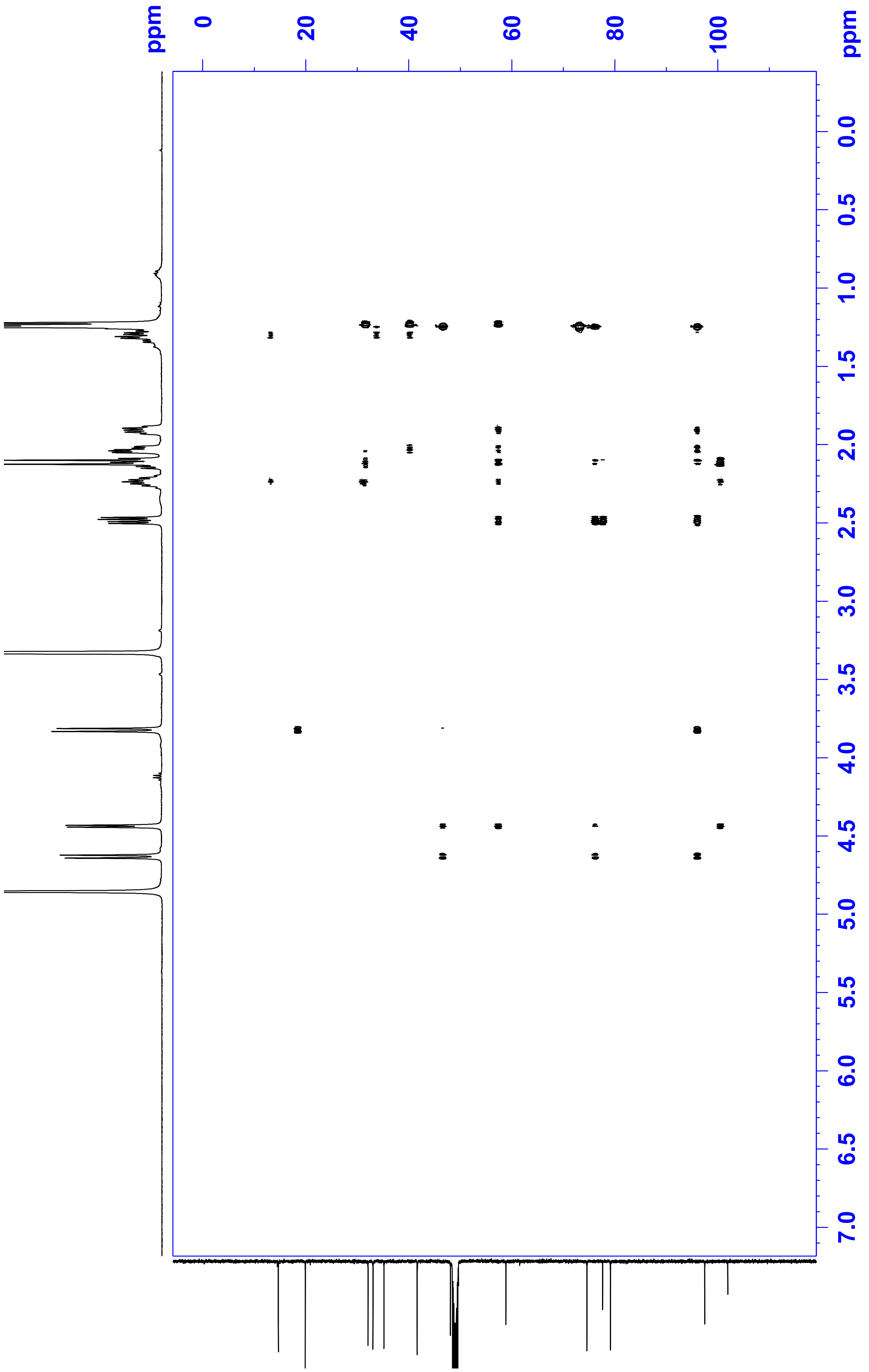

HSQC spectrum of jiadifenolide (500 MHz, MeOD)

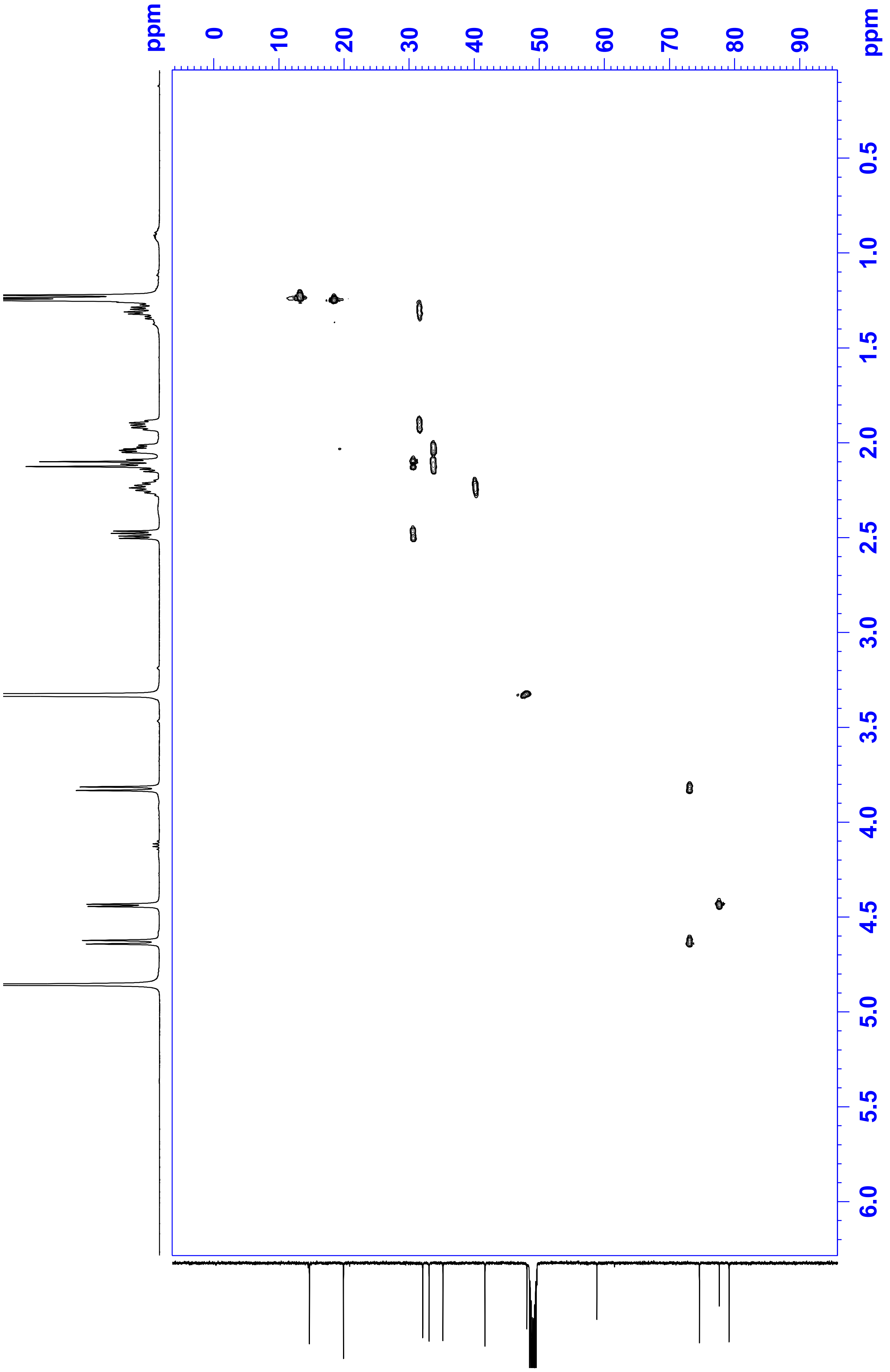

Supplement: Supplementary file 1 [file anie0053-7286-sd1.pdf]
